# Supplementary material for: Biological diversification linked to environmental stabilization following the Sturtian Snowball glaciation
Source: Sci Adv. 2023 Aug 25;9(34):eadf9999. doi: 10.1126/sciadv.adf9999 (PMC10456883; doi:10.1126/sciadv.adf9999)
Supplement: 20230825-1 [file sciadv.adf9999.v1.pdf]

GEOCHEMISTRY

Copyright © 2023 The Authors, some rights reserved; exclusive licensee American Association for the Advancement of Science. No claim to original U.S. Government Works. Distributed under a Creative Commons Attribution License 4.0 (CC BY).

Biological diversification linked to environmental stabilization following the Sturtian Snowball glaciation

Fred T. Bowyer[1,2]†*, Alexander J. Krause[2,3]†, Yafang Song[2], Kang-Jun Huang[4], Yong Fu[5], Bing Shen[6], Jin Li[7], Xiang-Kun Zhu[7], Michael A. Kipp[8], Lennart M. van Maldegem[9], Jochen J. Brocks[9], Graham A. Shields[3], Guillaume Le Hir[10], Benjamin J. W. Mills[2], Simon W. Poulton[2]

The body fossil and biomarker records hint at an increase in biotic complexity between the two Cryogenian Snowball Earth episodes (ca. 661 million to ≤650 million years ago). Oxygen and nutrient availability can promote biotic complexity, but nutrient (particularly phosphorus) and redox dynamics across this interval remain poorly understood. Here, we present high-resolution paleoredox and phosphorus phase association data from multiple globally distributed drill core records through the non-glacial interval. These data are first correlated regionally by litho- and chemostratigraphy, and then calibrated within a series of global chronostratigraphic frameworks. The combined data show that regional differences in postglacial redox stabilization were partly controlled by the intensity of phosphorus recycling from marine sediments. The apparent increase in biotic complexity followed a global transition to more stable and less reducing conditions in shallow to mid-depth marine environments and occurred within a tolerable climatic window during progressive cooling after post-Snowball super-greenhouse conditions.

INTRODUCTION

The end of the Sturtian cryochron [ca. 661 million years (Ma) ago], during which global mean temperature increased from Snowball to super-greenhouse conditions, documents one of the most profound paleoclimatic shifts in Earth's history (*1*). In the subsequent Cryogenian non-glacial interval (ca. 661 Ma to ≤650 Ma ago), molecular biomarker data record an increase in sterane diversity and abundance inferred to represent the transition from dominant bacterial to dominant green algal primary productivity. A proposed trigger for this fundamental ecological revolution is an increase in weathering-derived P availability following Sturtian deglaciation, although the appearance of mesophilic marine algae may have been delayed at tropical latitudes as a direct consequence of post-Snowball super-greenhouse conditions (*2*, *3*). The radiation of green algae may then have expanded benthic habitats and created more efficient food sources for the subsequent appearance of larger, metabolically active organisms (*2*).

While intuitive, this hypothesized coevolution of biotic and geochemical change has remained difficult to test empirically, for two main reasons. The first is the absence of a unified global chronostratigraphic framework with which to correlate all non-glacial sedimentary successions. While this interval benefits from numerous high-resolution regional lithostratigraphic, sequence stratigraphic, and chemostratigraphic studies [e.g., see (*4*–*15*)], no fully integrated global correlation framework or age model exists to explore the potential regional versus global trends in data and biotic records of coeval carbonate and siliciclastic successions (*4*). The second limitation concerns the absence of essential geochemical information for this interval with which to resolve coupled paleoredox and P dynamics. Therefore, despite considerable effort, the evolution of regional versus global paleoenvironmental redox conditions remains poorly understood, and attendant changes in the supply and recycling of nutrients (particularly P) remain unconstrained.

To address these challenges, we first provide a series of possible global lithostratigraphic and chemostratigraphic age models for the Cryogenian non-glacial interval, to fully calibrate inter-regional geochemical and fossil data, and to explore ongoing uncertainties. We then present the first high-resolution multiproxy paleoredox and phosphorus dataset for this interval, from five drill core profiles that document marine sedimentary successions in Australia and South China. For each core, we interpret regional redox dynamics using integrated records of Fe speciation, trace metal concentrations, and pyrite sulfur isotopes ($\delta^{34}S_{py}$), before assessing associated changes in the degree of bioavailable P recycling via P phase association analyses. Last, we calibrate our data alongside pertinent published geochemical data within each global age framework to provide a series of possible timelines for the coevolution of the biosphere and changes to the regional and global geochemical environment throughout this interval. Trends in the time-calibrated data compilations are considered relative to the modeled duration of post-Sturtian climatic recovery associated with the silicate weathering feedback. This enables a fully integrated assessment of the relative timing of geochemical and climatic stabilization, allowing links

[1]School of GeoSciences, University of Edinburgh, James Hutton Road, Edinburgh EH9 3FE, UK. [2]School of Earth and Environment, University of Leeds, Leeds LS2 9JT, UK. [3]Department of Earth Sciences, University College London, London WC1E 6BT, UK. [4]Department of Geology, Northwest University, 229 North Taibai Road, Xi'an 710069, Shaanxi Province, China. [5]College of Resource and Environmental Engineering, Key Laboratory of Karst Georesources and Environment, Ministry of Education, Guizhou University, Guiyang 550025, China. [6]Ministry of Education Key Laboratory of Orogenic Belts and Crustal Evolution, School of Earth and Space Sciences, Peking University, Beijing 100871, China. [7]MNR Key Laboratory of Isotope Geology, MNR Key Laboratory of Deep-Earth Dynamics, Institute of Geology, Chinese Academy of Geological Sciences, Beijing 100037, China. [8]Division of Geological and Planetary Sciences, California Institute of Technology, 1200 East California Boulevard, Pasadena, CA 91125, USA. [9]Research School of Earth Sciences, The Australian National University, Canberra, ACT 2601, Australia. [10]Université Paris, Institut de Physique du Globe de Paris, CNRS, 1 rue Jussieu, 75005 Paris, France.
*Corresponding author. Email: fred.bowyer@ed.ac.uk
†These authors contributed equally to this work.

to changes in the biotic record to be interrogated through the non-glacial interval.

RESULTS

Global correlation of Cryogenian non-glacial successions

The timings of Sturtian glaciation (ca. 717 Ma ago) and deglaciation (ca. 661 Ma ago) are relatively well calibrated by U-Pb and Re-Os geochronology on multiple continents (Fig. 1) (*16*–*18*). However, the precise timing for the onset of reglaciation associated with the Marinoan cryochron remains uncertain (*4*, *17*, *19*). Current best estimates from U-Pb geochronology suggest that the onset of Marinoan glaciation occurred after 651.69 ± 0.64 Ma in Laurentia (*19*), after 651.2 ± 3.3 Ma in South China (*20*), and before 639.29 ± 0.26 Ma on the Congo Craton (*21*). These radiometric ages permit possible non-glacial durations of between ca. 10 Ma and 21 Ma. Therefore, we present a series of age models that encompass the full range of possible non-glacial durations (models A to D; fig. S1 and table S1). Model A is displayed throughout the main text, wherein the non-glacial duration is set at ca. 11 Ma (see the Supplementary Materials). However, the relative trends in geochemical data and patterns of fossil occurrence are consistent between each model (set by the global correlation of regional lithostratigraphic composite profiles; Fig. 1F). The age models presented assume globally synchronous timings for glaciation and deglaciation, which are currently justified on the basis of available geochronological constraints and associated uncertainties, and modeled estimates of Snowball Earth dynamics (see Discussion, and Materials and Methods).

Trends in $\delta^{13}C_{carb}$ and $^{87}Sr/^{86}Sr$ during the Cryogenian non-glacial are well established and described from carbonate-dominated successions globally [e.g., see (*4*–*6*, *8*, *11*, *22*, *23*)]. These trends are reproduced in our age models and summarized briefly to aid reference. Each named $\delta^{13}C_{carb}$ excursion recorded throughout the Cryogenian non-glacial interval corresponds to a positive or negative deviation from 0‰, and the absolute durations of each excursion remain uncertain (see below and Materials and Methods). The non-glacial record begins in strata deposited atop Sturtian diamictites with the Rasthof negative $\delta^{13}C_{carb}$ anomaly [Fig. 1B; note that this is renamed the "Twitya carbon isotope excursion (CIE)" or Cryogenian CIE "Cn1" by Hoffman *et al.* (*4*)]. This excursion is globally reproducible but variable in magnitude with minimum values exhibited in mixed siliciclastic-carbonate successions of Australia and Laurentia [see below; (*7*, *13*)]. The duration of recovery from the Rasthof anomaly toward positive $\delta^{13}C_{carb}$ values ["Cn2" of (*4*)] is poorly constrained because of a dearth of available radiometric ages in carbonate successions (Fig. 1B). Positive $\delta^{13}C_{carb}$ values are subsequently interrupted by the negative Taishir anomaly ("Cn3" of (*4*)]. The magnitude of the Taishir anomaly is highly variable with the most negative $\delta^{13}C_{carb}$ values exhibited at the base of Taishir Unit 3 in the carbonate-dominated Zavkhan terrane, Mongolia (*8*). The Taishir anomaly appears to coincide with a transition from highstand to transgressive deposition in multiple regions (e.g., Arctic Alaska, Congo craton, and Zavkhan Terrane; Fig. 1F). The subsequent interval of positive $\delta^{13}C_{carb}$, termed the "Keele Peak" ["Cn4" of (*4*)], also exhibits highly variable magnitude, ranging from 0 to 1‰ to >11‰. A maximum depositional age of 651.69 ± 0.64 Ma (zircon U-Pb chemical abrasion isotope dilution thermal ionization mass spectrometry) constrains $\delta^{13}C_{carb}$ values that may be associated with the Keele Peak in the upper Kingston Peak Formation of Laurentia [see the Supplementary Materials; (*13*, *19*)].

Variability in the magnitude of the Taishir anomaly and Keele Peak between sections has previously been examined in detail within the context of regional lithostratigraphy and facies of the Otavi Group of the Congo craton, northern Namibia (*4*, *12*), but is clearly demonstrated on other cratons. The differences in magnitude but general consistency of trends in $\delta^{13}C_{carb}$ have been suggested to reflect facies-related differential diagenesis (*4*, *12*, *13*, *23*). Following the Keele Peak, numerous global successions record a prominent downturn in $\delta^{13}C_{carb}$, termed the "Trezona" anomaly ["Cn5" of (*4*)], which reaches a nadir approaching −12‰ in Laurentia and Australia (Fig. 1B). As discussed in numerous studies, Marinoan glacial erosion is a likely cause for the variable continuity of carbonate successions (e.g., truncated in Mongolia) and thus the completeness of the preserved Trezona anomaly, in the aftermath of the Keele Peak (*4*, *8*).

The $\delta^{13}C_{carb}$ age framework directly anchors $^{87}Sr/^{86}Sr$ data in carbonate samples from the Congo craton, Laurentia, Zavkhan terrane, and Arctic Alaska (*6*, *8*, *11*, *16*). The resulting $^{87}Sr/^{86}Sr$ profile is consistent with previous compilations (*6*, *8*, *16*, *22*) and shows initially low values of ~0.7067 immediately following Sturtian deglaciation with a gradual increase to ~0.7074 before the onset of Marinoan glaciation (Fig. 1C).

Cryogenian paleotemperature

The initiation of global deglaciation at the termination of the Sturtian and Marinoan cryochrons required super-greenhouse conditions driven by the long-term build-up of atmospheric CO_2 (*1*). Model estimates of the subsequent decline in atmospheric CO_2 concentrations in the aftermath of Cryogenian Snowball glaciations have accounted for a range of possible rates of CO_2 drawdown via the silicate weathering feedback, associated with differences in weathering profiles and runoff (Fig. 1D) (*24*). Using these pCO_2 values in the ocean-atmosphere general circulation climate model FOAM (*25*) yields an envelope of minimum and maximum global mean surface temperature change associated with decreasing atmospheric pCO_2 throughout the Cryogenian non-glacial interval (Fig. 1D and table S1). While associated uncertainty envelopes are large, these model runs reveal initially high global average temperatures (≥48°C), associated with post-Sturtian super-greenhouse conditions, to lower temperatures (~6° to 27°C) in the prelude to Marinoan reglaciation (Fig. 1D). The lower bound of this uncertainty envelope (solid black line in Fig. 1D) represents the pCO_2 evolution most consistent with the conditions required to initiate the Marinoan Snowball Earth glaciation and corresponds to the presence of a highly reactive surface (due to glacial rock flour) in the upper layers of the soil (*24*).

Cryogenian biostratigraphy

Figure 1E shows a schematic depiction of the age ranges of key biomarker data, microfossil assemblages (including problematica), and problematic macrofossil occurrences throughout the Cryogenian non-glacial interval that result from the global lithostratigraphic and chemostratigraphic correlation. We do not attempt to interrogate the biological affinity of any putative fossils but simply show the relative ages of each reported specimen/assemblage (table S1). The non-glacial fossil record is dominated by a low-diversity microfossil assemblage, largely constrained to the Cn1 to Cn3 interval

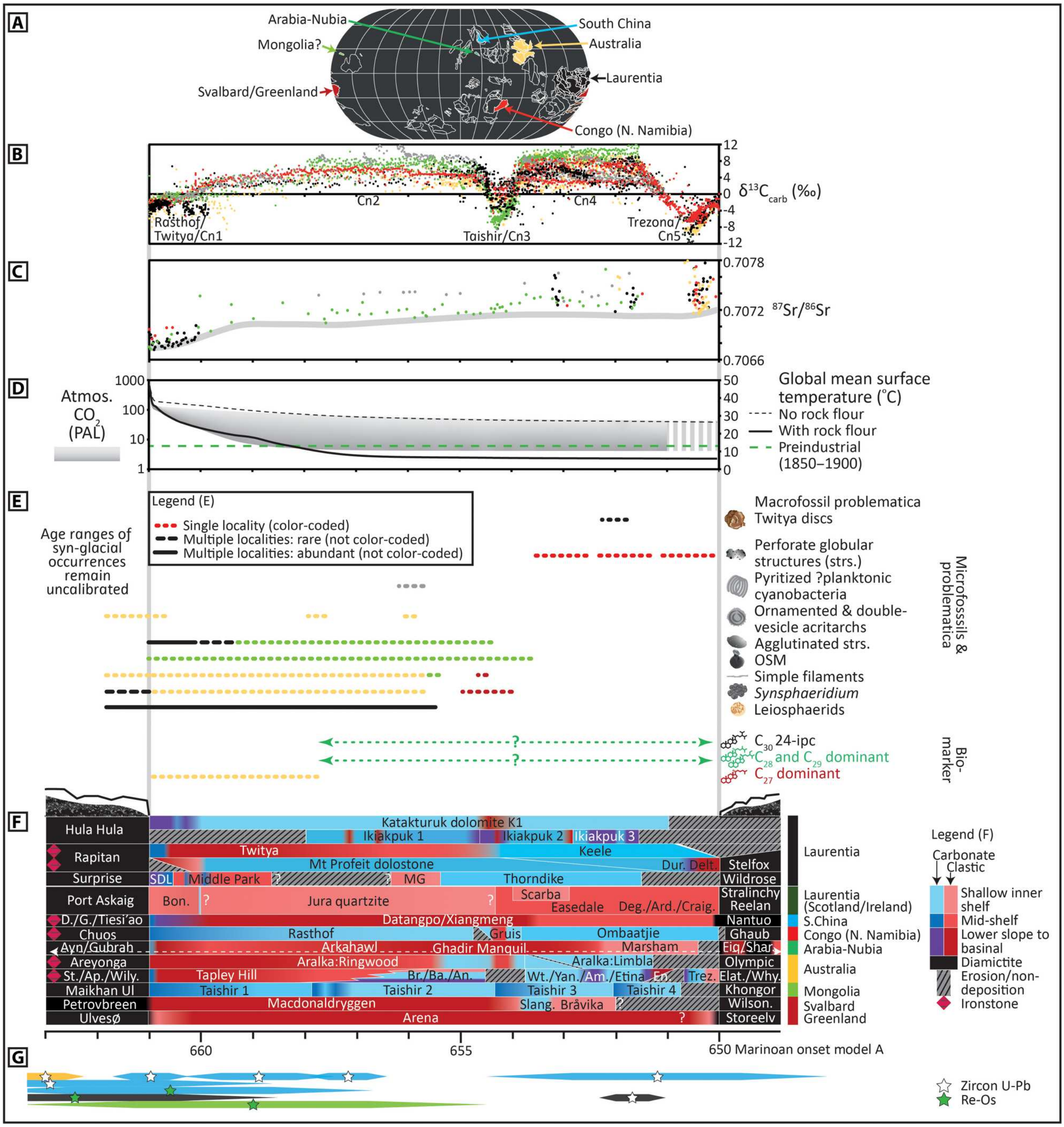

Fig. 1. Integrated chronology and biostratigraphy of globally distributed Cryogenian non-glacial successions (model A; table S1). (**A**) Paleogeography after (*77*), but note ongoing uncertainties associated with precise positions of some cratons (especially South China and the Congo craton) (*1*, *4*). All subsequent data are color-coded by provenance (region/craton) as indicated in (A) and by colored bars to the right of (F). (**B**) Global composite $\delta^{13}C_{carb}$ profile based on regional lithostratigraphic and chemostratigraphic correlation, followed by subsequent best-fit to data trends exhibited in the most continuous globally distributed successions. (**C**) Global $^{87}Sr/^{86}Sr$ chemostratigraphy calibrated directly within the $\delta^{13}C_{carb}$ age model. (**D**) Range in possible atmospheric CO_2 concentration after (*24*), shaded to indicate likelihood based on corresponding temperature relationship. Expected changes in global mean surface temperature based on FOAM (fast ocean-atmosphere model) CO_2-temperature relationship. The solid black line represents the CO_2-temperature scenario most consistent with conditions required to initiate the Marinoan Snowball glaciation and corresponds to the presence of a highly reactive surface of glacial rock flour [see (*24*) for detailed methods]. (**E**) Fossil occurrences, including putative body fossils, microfossils, problematica, and biomarkers (full references and model ages in table S1). The maximum age for the first appearance of biomarker data from a shale package 150 m below (or within) a Marinoan-age diamictite of the Ghadir Manqil Formation, Oman, remains poorly constrained (*29*). OSM, organic spore-like microfossils (*28*). (**F**) Regional composite lithostratigraphic correlation, color-coded by dominant lithology and paleodepth interpretation (see legend). Colored bars to the right show the color coding of data and information in (A) to (C), (E), and (G). (**G**) Radiometric geochronology (U-Pb and Re-Os) used to calibrate the timing of global Sturtian deglaciation and anchor magnitudes and trends in $\delta^{13}C_{carb}$ in this age model (table S3). See Materials and Methods for details of age model construction and tables S1 and S3 for compiled data and full references.

(*26*–*28*). Sediments that immediately overlie Sturtian glacial deposits contain biomarker data that are restricted to traces of cholestanes (C_{27} steranes) possibly indicating minimal red algal activity (*2*). The subsequent first appearances of green algal (C_{28} and C_{29}) and putative sponge (C_{30}) steranes in pre-Marinoan to Marinoan-equivalent shales of Oman [(*29*) but see (*30*)] are poorly constrained relative to the $\delta^{13}C_{carb}$ profile but are likely younger than Cn1 based on their absence from postglacial deposits of the lower Aralka Formation (*2*). Reported occurrences of putative macrofossils, including aspidellamorph "Twitya discs" in the lower Ice Brook Formation of Laurentia [e.g., see (*31*)], are restricted to the latter half of the Cryogenian non-glacial interval, from Cn3 to Cn5 (table S1).

Geological and stratigraphic context of sampled drill cores

We report geochemical data from 295 drill core samples that span the Cryogenian non-glacial interval, recovered from five siliciclastic-dominated sections deposited on two paleocontinents (fig. S2). The analyzed material includes samples of the Aralka Formation of the Amadeus Basin (Centralian Superbasin) and Tapley Hill Formation of the Adelaide Superbasin, Australia, and the Datangpo and Xiangmeng formations of the Nanhua Basin, South China. Full details of all analyzed drill cores are presented in the Supplementary Materials and are briefly outlined below. Additional published data compiled in Figs. 2 to 5 are presented in table S1.

The Amadeus Basin and Adelaide Superbasin, Australia

Three drill cores were investigated from the Amadeus Basin of the Centralian Superbasin (BR05-DD01 and Wallara-1) and the Stuart Shelf of the Adelaide Superbasin (SCYW-79-1A). In the central-western Amadeus Basin, core BR05-DD01 comprises Sturtian glacially influenced diamictites of the Areyonga Formation, overlain by ~331 m of mid-outer ramp dolomitic shale and siltstone, with rare stromatolitic carbonate interbeds of the Aralka Formation (*9*). The lower ca. 243 m of the Aralka Formation in core BR05-DD01 host low sterane/hopane ratios (≤0.012), where eukaryotic steranes are limited to cholestane (*2*). Red siltstones that dominate the uppermost ~100 m of the sampled interval may represent progressive shallowing of the upper Aralka Formation during the non-glacial or may correlate with the Ediacaran-Cambrian Arumbera sandstone (see the Supplementary Materials). Sediments of the Wallara-1 core were deposited in the Amadeus Basin to the east of BR05-DD01 and constitute a similar but condensed lithological profile, comprising diamictite of the Areyonga Formation overlain by dolomitic shale of the lowermost Aralka Formation.

Core SCYW-79-1A recovers strata of the northwest Adelaide Superbasin, along the northeastern Stuart Shelf. In this core, Sturtian glaciogenic deposits of the lower Umberatana Group (Yudnamutana Subgroup) are represented by the Appila tillite (*32*, *33*) and are conformably overlain by non-glacial carbonaceous shales and siltstones of the Tapley Hill Formation (Nepouie Subgroup). The Tapley Hill Formation was deposited time-equivalent to the Aralka Formation of the Amadeus basin, and correlative transgressive siliciclastic deposits throughout the Centralian Superbasin (*33*). In addition to the high-resolution record obtained for SCYW-79-1A, we also provide data for the Tapley Hill Formation from a second core recovered from the Adelaide Superbasin (SR/17-2). Because of low sampling resolution, we do not discuss this core in detail, but the results are provided in the supplementary dataset (tables S4 and S5) and the trends in all associated datasets mirror those obtained from SCYW-79-1A.

The Nanhua Basin, South China

The Nanhua Basin developed along the southeast margin of the Yangtze Block, South China, as a consequence of rifting during the Tonian break-up of Rodinia (*34*). The most complete Cryogenian sections of the Nanhua Basin were deposited within the Hunan-Guangxi sub-basin in eastern Guizhou Province and western Hunan Province. Here, the succession includes syn-glacial deposits of the Sturtian (including the Gucheng, Tiesi′ao, Dongshanfeng, Chang′an, and Fulu formations) and Marinoan (Nantuo Formation) cryochrons, separated by the non-glacial Datangpo and Xiangmeng formations (*14*, *17*, *34*).

We present data from two drill core profiles that comprise continuous siliciclastic deposits of the Datangpo and Xiangmeng formations. Core ZK102 records outer shelf-slope shales and siltstones of the Datangpo Formation, while core ZK3603 records deeper, slope-basin shales of the Xiangmeng Formation. In both cores, the contacts between the Datangpo and Xiangmeng formations and the underlying and overlying diamictites appear to be conformable (*14*).

Geochemical results

Geochemical techniques are outlined in Materials and Methods, and further discussion, including a detailed evaluation of the robustness of the applied multiproxy approach, is provided in the Supplementary Materials. Sturtian-age deposits have highly reactive to total iron (Fe_{HR}/Fe_T) ratios of >0.22 and pyrite to highly reactive (Fe_{py}/Fe_{HR}) ratios of <0.60 (Cores BR05-DD01, Wallara-1, and SCYW-79-1A; Figs. 2 and 3). At the lithological transition between glacial and non-glacial deposits, all studied cores record prominent and synchronous increases in Fe_{HR}/Fe_T and Fe_{py}/Fe_{HR} ratios, and total organic carbon (TOC) and authigenic molybdenum (Mo_{auth}) concentrations, which represent Mo enrichment relative to the detrital background flux (Figs. 2 to 4; see Supplementary Materials for methods). Fe_{py}/Fe_{HR} ratios peak in the lowermost siliciclastic-dominated intervals of each non-glacial succession, culminating in values that are ubiquitously >0.60. This interval is also associated with a peak in $\delta^{34}S_{py}$ and a negative $\delta^{13}C_{org}$ excursion and is time-equivalent to the Rasthof anomaly based on its position immediately overlying Sturtian diamictite (Figs. 1, 2, and 4). Following this initial peak, the most continuous cores deposited in shelf settings (BR05-DD01, SCYW-79-1A, and ZK102) exhibit progressive decreases in Fe_{HR}/Fe_T ratios that approach values of <0.38, accompanied by decreasing Fe_{py}/Fe_{HR} (<0.60) and decreasing Mo_{auth} (Figs. 2 to 4). By contrast, all samples from the most distal core (ZK3603) continue to record Fe_{HR}/Fe_T > 0.38. In this core, Fe_{py}/Fe_{HR} ratios oscillate from <0.60 to >0.60 with low Mo_{auth} throughout.

Australian cores record total phosphorus (P_{Tot}) concentrations that approximate, or slightly exceed, the average Phanerozoic shale (AS) value [Amadeus Basin mean = 684 parts per million (ppm), Adelaide Superbasin mean = 951 ppm; AS = 700 ppm; (*35*)], but normalization to Al suggests that the majority of samples are elevated relative to average shale (Amadeus Basin mean = 0.013, Adelaide Superbasin mean = 0.017; AS = 0.008; Figs. 2, G and Q, and 3G) (*35*). By contrast, cores from South China are depleted in P_{Tot} relative to average shale, both in absolute

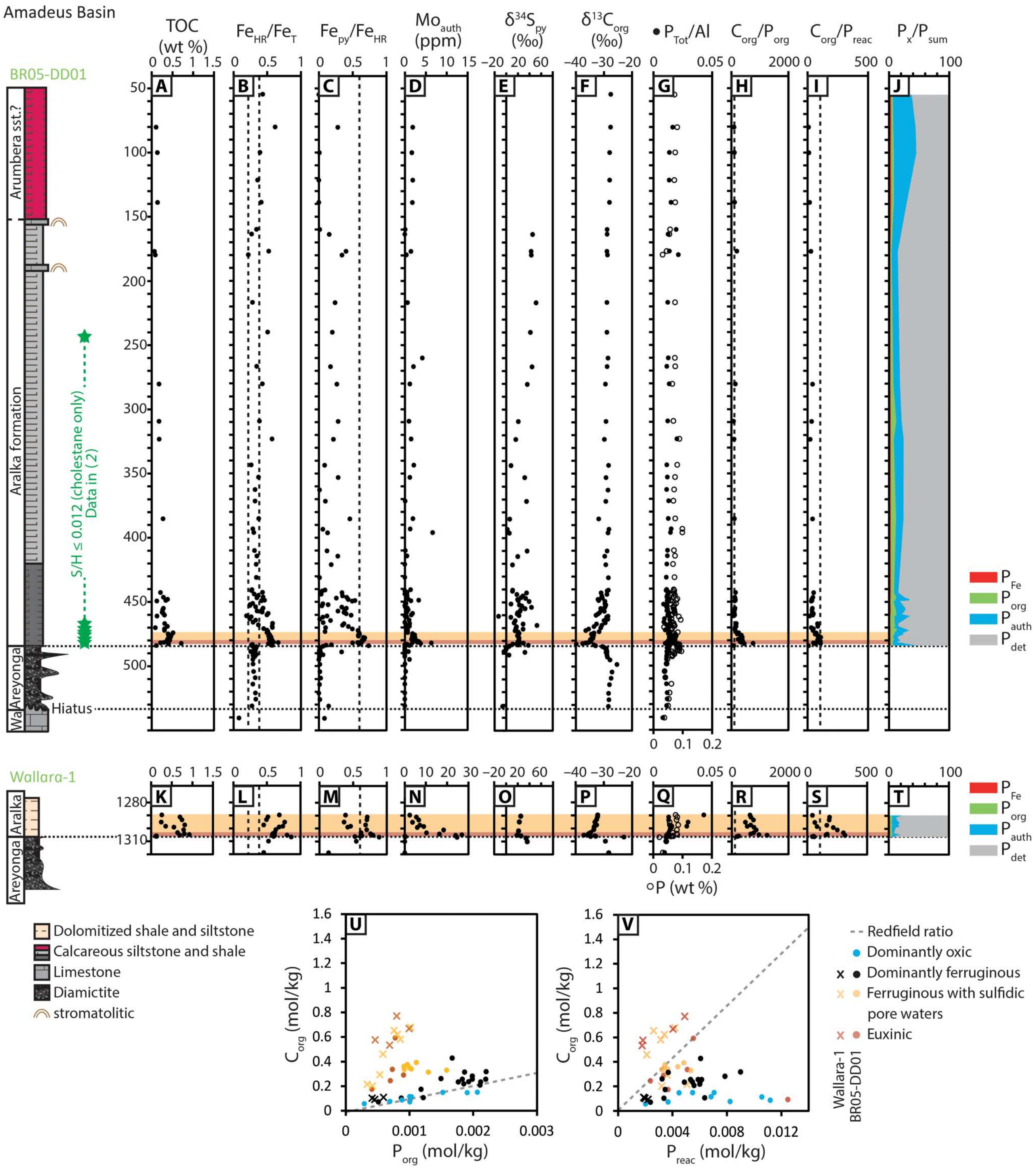

Fig. 2. Stratigraphy and selected geochemical data for the Aralka Formation of the Amadeus Basin recovered by drill cores BR05-DD01 and Wallara-1. Data include (**A** and **K**) total organic carbon (TOC), (**B**, **C**, **L**, and **M**) Fe speciation, (**D** and **N**) authigenic Mo concentrations (Mo_{auth}), (**E** and **O**) $\delta^{34}S_{py}$, (**F** and **P**) $\delta^{13}C_{org}$, and (**G** to **J** and **Q** to **V**) P speciation. Horizontal red bands indicate the intervals of euxinic water column conditions, and horizontal yellow bands indicate the intervals of ferruginous water column conditions with sulfidic pore waters (detailed redox interpretation is provided in the Supplementary Materials and table S2). Vertical dashed lines in (B), (C), (L), and (M) correspond to calibrated threshold ratios for paleoredox interpretation by Fe speciation, detailed in Materials and Methods. Vertical dashed lines in (H), (I), (R), and (S) correspond to the Redfield ratio. Wa, Wallara Formation. Positions of samples analyzed for biomarker data in core BR05-DD01 (*2*) indicated by green stars. S/H, sterane/hopane ratio.

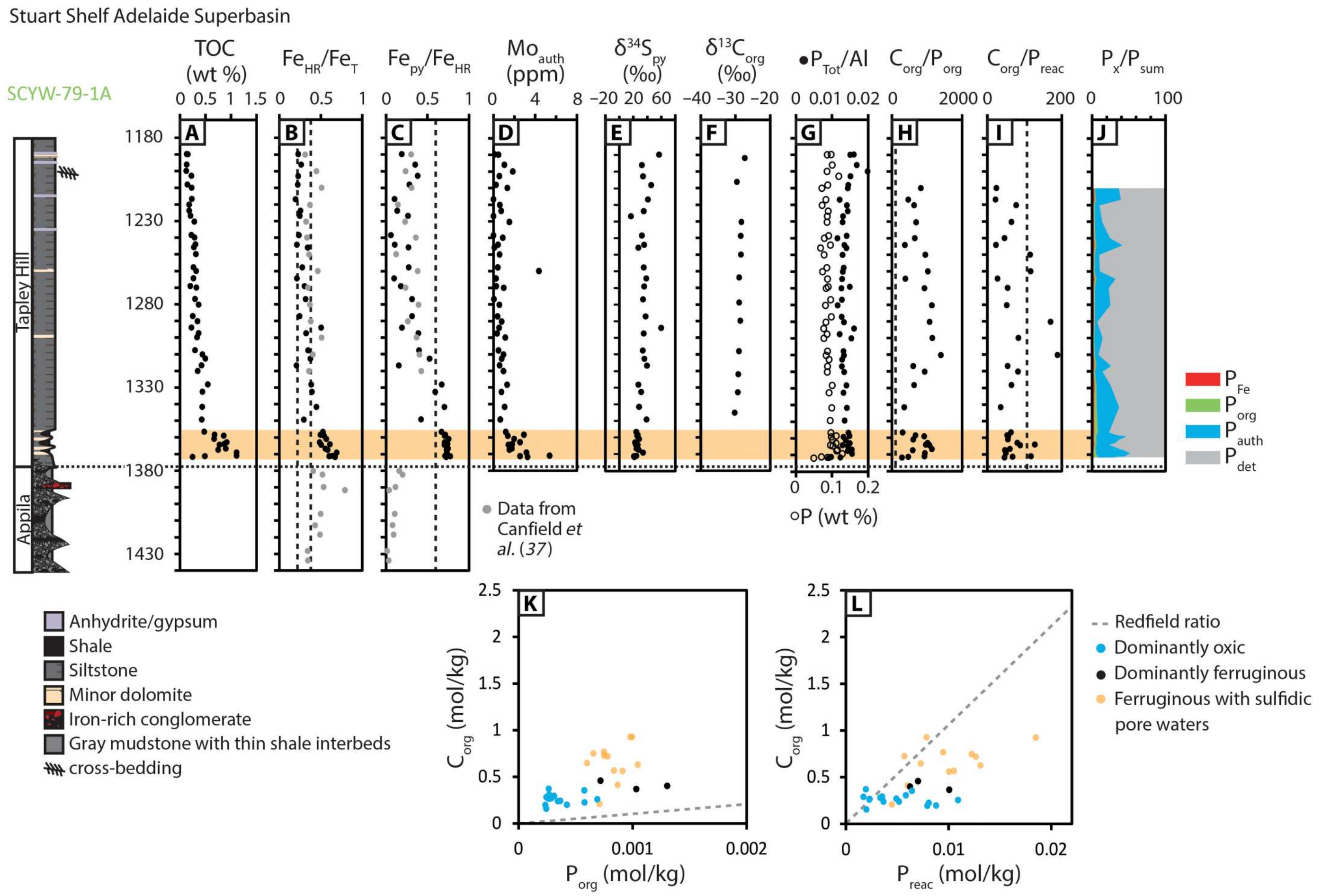

Fig. 3. Stratigraphy and selected geochemical data for the Tapley Hill Formation recovered by drill core SCYW-79-1A. Data include (**A**) TOC, (**B** and **C**) Fe speciation, (**D**) Mo_{auth}, (**E**) $\delta^{34}S_{py}$, (**F**) $\delta^{13}C_{org}$, and (**G** to **L**) P speciation. Additional Fe speciation data are from Canfield *et al.* (*37*) and $\delta^{34}S_{py}$ are from Gorjan *et al.* (*15*). Horizontal red bands indicate the intervals of euxinic water column conditions, and horizontal yellow bands indicate the intervals of ferruginous water column conditions with sulfidic pore waters (detailed redox interpretation is provided in the Supplementary Materials and table S2). Vertical dashed lines in (B) and (C) correspond to calibrated threshold ratios for paleoredox interpretation by Fe speciation, detailed in Materials and Methods. Vertical dashed lines in (H) and (I) correspond to the Redfield ratio.

concentration (mean = 454 ppm) and after normalization to Al (mean = 0.005) (Fig. 4, G and Q). The available samples in each environment record minor increases in P_{Tot} and P_{Tot}/Al across the transitionary interval from Sturtian-aged glacial deposits to overlying non-glacial shales (Figs. 2 to 4). In South China, this is represented by a short-lived positive excursion at the conformable boundary between the Tiesi'ao and Datangpo formations, followed by recovery to low values, but high values are maintained in Australian cores throughout the duration of non-glacial deposition following this initial increase.

Shelf cores (BR05-DD01, Wallara-1, SCYW-79-1A, and ZK102) show consistent stratigraphic trends in the ratios of organic carbon to organic phosphorus (C_{org}/P_{org}) and organic carbon to reactive phosphorus (C_{org}/P_{reac}), where P_{reac} equates to the sum of phosphorus bound in iron phases (P_{Fe}), authigenic carbonate fluorapatite, biogenic apatite and calcium carbonate (P_{auth}), and organic matter (P_{org}) (*36*). The observed trends in C_{org}/P_{org} and C_{org}/P_{reac} largely mirror the trends observed in paleoredox data, whereby peak values of C_{org}/P_{org} and C_{org}/P_{reac} in the lowermost non-glacial deposits are followed by decreasing values in overlying strata (Figs. 2 to 4). In core SCYW-79-1A, C_{org}/P_{org} values recover and remain scattered throughout the sampled non-glacial interval (Fig. 3H). By contrast, C_{org}/P_{org} and C_{org}/P_{reac} ratios in the most distal core (ZK3603) remain elevated throughout, with a possible decreasing trend in C_{org}/P_{org} toward the top of the non-glacial succession (Fig. 4, R and S). The analyzed cores show regional distinction in P phase associations, whereby Australian cores (BR05-DD01, Wallara-1, and SCYW-79-1A) are dominated by P_{det} and subordinate P_{auth} (Figs. 2, J and T, and 3J), while cores from South China show a lesser P_{det} contribution relative to P_{auth} (Fig. 4, J and T). The most distal core (ZK3603) also shows a substantial contribution from P_{Fe} (Fig. 4T).

DISCUSSION

Tracking the evolution of non-glacial water column and pore water redox conditions

Shelf-to-slope environments in Australia and South China display markedly similar trends in Fe speciation and redox-sensitive trace metal datasets across the transition from syn-glacial to postglacial

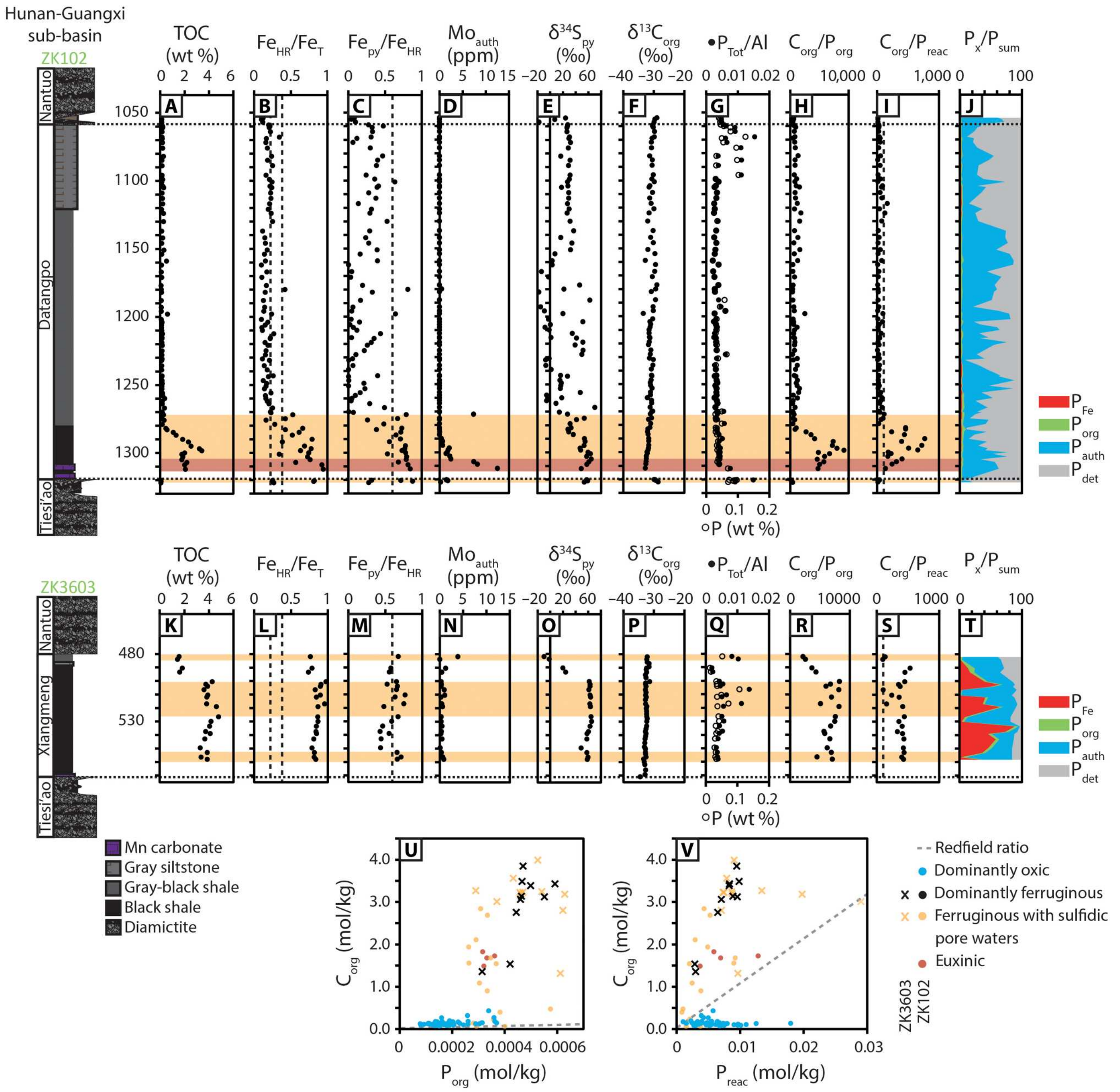

Fig. 4. Stratigraphy and selected geochemical data for the Datangpo and Xiangmeng formations recovered by drill cores ZK102 and ZK3603, respectively. Data include (**A** and **K**) TOC, (**B**, **C**, **L**, and **M**) Fe speciation, (**D** and **N**) Mo_{auth}, (**E** and **O**) $\delta^{34}S_{py}$, (**F** and **P**) $\delta^{13}C_{org}$, and (**G** to **J** and **Q** to **V**) P speciation. Additional $\delta^{13}C_{org}$ data for ZK3603 are from Peng *et al.* (*14*). Horizontal red bands indicate the intervals of euxinic water column conditions, and horizontal yellow bands indicate the intervals of ferruginous water column conditions with sulfidic pore waters (detailed redox interpretation is provided in the Supplementary Materials and table S2). Vertical dashed lines in (B), (C), (L), and (M) correspond to calibrated threshold ratios for paleoredox interpretation by Fe speciation, detailed in Materials and Methods. Vertical dashed lines in (I) and (S) correspond to the Redfield ratio.

deposition (Figs. 2 to 4 and figs. S3 to S5). Compiled Fe speciation data from limited syn-glacial shale interbedded with Sturtian-age diamictite in Australia and South China support deposition under equivocal (Fe_{HR}/Fe_T = 0.22 to 0.38) to anoxic and ferruginous (Fe_{HR}/Fe_T > 0.38, Fe_{py}/Fe_{HR} < 0.60) water column conditions (Figs. 2 to 4), consistent with previous Fe speciation studies (*37*). Dominantly ferruginous subglacial conditions are also consistent with models for the genesis of syn-glacial Fe formations (*32*, *37*, *38*). In the immediate aftermath of Sturtian deglaciation, elevated Fe_{HR}/Fe_T ratios support anoxic depositional conditions (*39*) (see the Supplementary Materials for expanded discussion). Elevated Fe_{py}/Fe_{HR} ratios and redox-sensitive trace element enrichments support sulfidic pore water conditions in all cores (Figs. 2 to 4 and figs. S4 and S5), and moderate to high Mo_{auth} concentrations, which require appreciable free sulfide, suggest short-lived intervals of euxinia in some cores (BR05-DD01, Wallara-1, and ZK102; Figs.

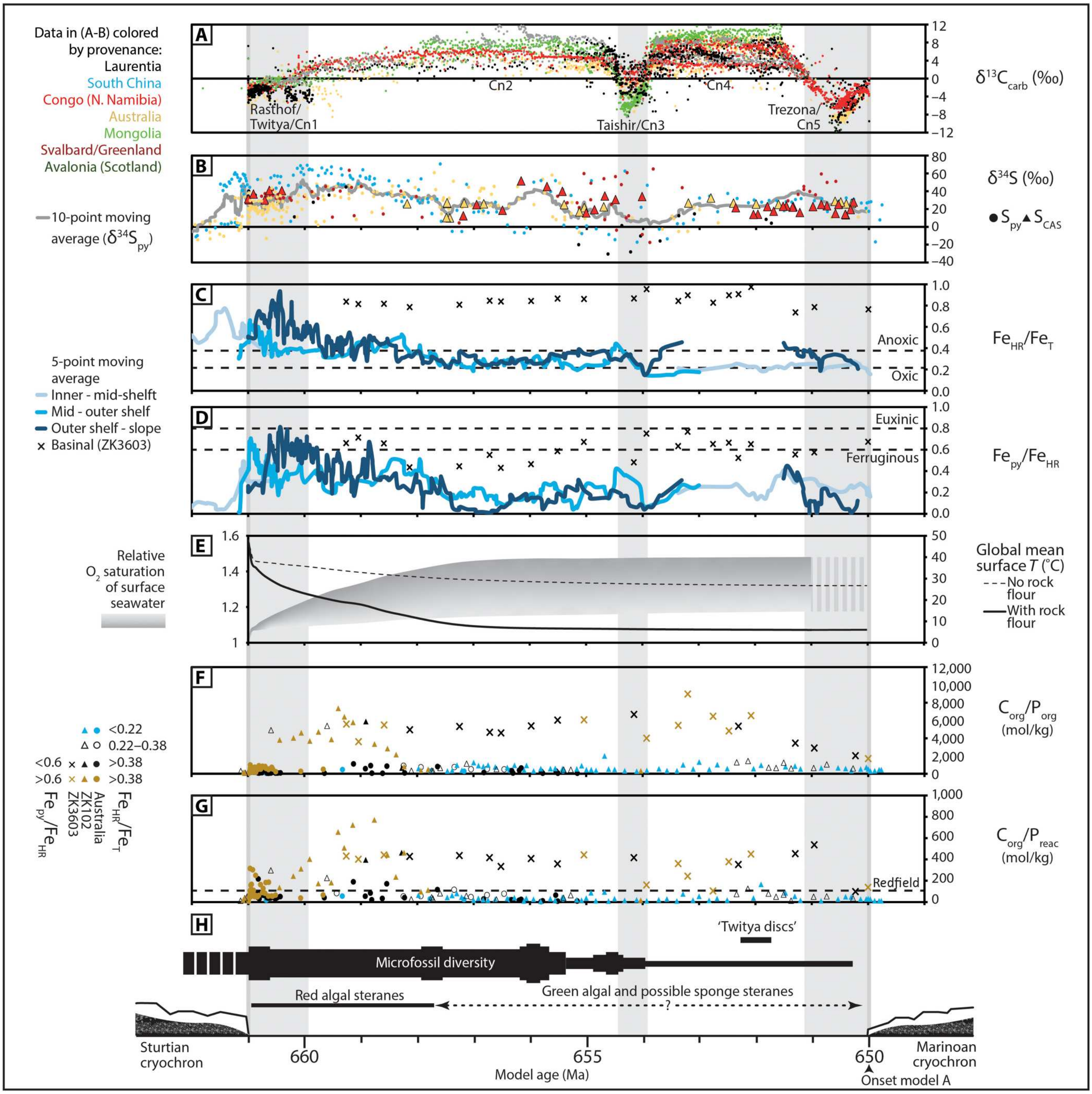

Fig. 5. High-resolution global age model compilation of selected geochemical proxy data (published and herein), modeling results, and fossil occurrences (following model A). (**A**) $\delta^{13}C_{carb}$, (**B**) $\delta^{34}S_{py}$, (**C**) Fe_{HR}/Fe_T, (**D**) Fe_{py}/Fe_{HR}, (**E**) global mean surface temperature and calculated relative O_2 saturation of surface seawater (gray envelope) based on FOAM CO_2-temperature relationship, (**F**) C_{org}/P_{org}, (**G**) C_{org}/P_{reac}, and (**H**) fossil occurrences summarized from Fig. 1 and references therein (occurrences calibrated directly within each age model; table S1). Data in (A) and (B) are color-coded according to provenance (see Fig. 1). Data in (F) and (G) are color-coded according to threshold ratios of Fe speciation data in the same samples (see legend). (C) and (D) show the five-point moving averages for a global compilation of Fe speciation data, binned by depositional environment (see legend and table S1). Horizontal dashed lines in (C) and (D) represent the calibrated threshold ratios for deposition under different paleoredox conditions discussed in Materials and Methods (*39*, *74*). See table S1 for the full geochemical dataset, age model, and full references.

2 and 4). Short-lived euxinia at this level is also consistent with available information on the morphology and size distribution of framboidal pyrite in deposits of the Nanhua Basin (*40*). The close agreement between two independent paleoredox proxies (i.e., Fe speciation and Mo_{auth}) provides strong support for a robust paleoredox reconstruction (see the Supplementary Materials for expanded discussion). Where Fe_{HR}/Fe_T is >0.38 and Fe_{py}/Fe_{HR} is >0.60, but concentrations of Mo_{auth} are low to moderate, these data are conservatively interpreted to reflect the development of sulfidic pore waters beneath a ferruginous water column, where Mo enrichment was limited by sulfide availability and/or sedimentation rate (Figs. 2 to 4 and figs. S4 and S5) (*41*). However, during, and in the immediate aftermath of, Sturtian deglaciation, seawater Mo and U concentrations are highly likely to have been depleted as a consequence of long-term (ca. 56 Ma) trace metal drawdown under dominantly anoxic Snowball ocean conditions and/or intervals of semi-restriction (see fig. S3) (*42*). For this reason, some core intervals interpreted to record sulfidic pore waters within sediments deposited beneath a ferruginous water column may instead represent deposition beneath a euxinic water column (e.g., Wallara-1; Fig. 2 and fig. S4).

Following the short-lived euxinic/sulfidic interval, shelf-to-slope environments (BR05-DD01, SCYW-79-1A, and ZK102) record a gradual decrease in Fe_{HR}/Fe_T, Fe_{py}/Fe_{HR}, and Mo_{auth} that together suggest a trend toward less reducing conditions (Figs. 2 to 4). However, elevated Fe_{HR}/Fe_T (>0.38) in all samples from ZK3603 suggest that anoxia was maintained in the deepest environments throughout the non-glacial interval, with variable Fe_{py}/Fe_{HR} (0.43 to 0.77) and generally muted trace metal enrichments implying dominantly ferruginous conditions, with the occasional development of sulfidic porewaters during early diagenesis (Fig. 4 and fig. S5).

The majority of globally distributed successions record gradual shallowing throughout the Cn1 and Cn2 interval (e.g., see Fig. 1F), which may suggest that slow rates of deposition in the immediate aftermath of deglaciation promoted higher relative accumulation of organic carbon in anoxic deep waters, and this was followed by infill of accommodation space and gradual shallowing into less reducing surface to mid-depth waters. However, there are a number of lines of evidence to suggest that the trends in paleoredox data are not controlled solely by changes in a depositional rate. First, though each of our cores shows sedimentological evidence for shallowing, the trends in paleoredox data do not always correspond directly with recorded lithological shifts (see the Supplementary Materials). For example, in core ZK102, the transition from black to gray shale (ca. 1280 m) is not noted to correspond with an increase in mean grain size that may otherwise represent abrupt shallowing and associated depositional rate increase (Fig. 4). Hence, the geochemical trends recorded in underlying strata cannot simply be attributed to an increasing depositional rate. Second, diagenetic models suggest that muted Mo_{auth} would be expected in intervals of elevated sedimentation rate (*41*). However, our deepest core (ZK3603), which was likely deposited at the slowest rate when considering total core thickness, lithofacies, and deposition throughout the full non-glacial duration, exhibits persistently low Mo_{auth} despite Fe_{HR}/Fe_T > 0.38 and Fe_{py}/Fe_{HR} as high as 0.77 (e.g., see Fig. 4, M and N). If increasing sedimentation rates throughout the non-glacial were solely responsible for the observed decrease in Mo_{auth} in shelf-slope environments then moderate Mo_{auth} may be expected in deeper settings, where sedimentation rates remained low and pore waters were occasionally sulfidic (e.g., ZK3603), but this is not recorded. Last, a number of independent geochemical proxies have similarly been interpreted to record a transition toward less reducing conditions across this interval, including sedimentary selenium (Se) isotope data in successions of both Laurentia and Australia and Fe isotope data from the Nanhua Basin (*43*, *44*). In sum, trends in the reported geochemical data are most parsimoniously interpreted to represent temporal changes in the extent of reducing conditions rather than shifts associated with depositional rates.

Interrogating the importance of regional redox conditions for the evolution of the non-glacial P cycle

Phosphorus is generally accepted to be the ultimate limiting nutrient for primary productivity on geological time scales (*45*) with a principal source of phosphate to the marine system being via continental weathering. However, both persistent and transitory changes in marine redox conditions can profoundly alter the degree of syn-depositional and early diagenetic recycling of bioavailable phosphorus from sediments (*46*–*50*). The ability of bacteria and archaea to store P is reduced under anoxic conditions (*51*). Furthermore, microbial sulfate reduction under euxinic water column conditions, or within sulfidic sediment pore waters, promotes the preferential release of P from organic matter and limits the formation of unsulfidized Fe minerals to which P can readily re-adsorb (*36*, *51*, *52*). Sulfidic pore water conditions, elevated temperature, and reduced pH may also limit P uptake through inhibition of apatite authigenesis (*53*). In this way, sulfidic conditions, ocean warming, and acidification all have the potential to supplement nutrient input for primary production in surface waters by driving extensive bioavailable P recycling from sediments (*48*, *54*).

Given that the C_{org}/P_{org} of organic carbon delivered to the sediment is expected to approximate the Redfield ratio (106:1; see the Supplementary Materials), molar ratios of C_{org}/P_{org} in excess of the Redfield ratio cannot simply occur as a function of elevated organic carbon burial. Instead, molar ratios of C_{org}/P_{org} that exceed the Redfield ratio (e.g., see Figs. 2U, 3K, and 4U) indicate preferential P release during the anaerobic remineralization of organic matter, which is commonly particularly intense during microbial sulfate reduction (*51*). Phosphorus released by this process, and via reductive dissolution of Fe (oxyhydr)oxide minerals, can either be recycled to the water column or fixed in the sediment via "sink-switching" to authigenic phases, either in the form of carbonate fluorapatite, via uptake to Fe (oxyhydr)oxide minerals close to the sediment-water interface, or via precipitation of vivianite ($(Fe_3PO_4)_2.8H_2O$) (*55*–*58*). The degree to which bioavailable P is recycled to the water column relative to organic carbon is indicated by the ratio of C_{org}/P_{reac}, whereby values higher than the Redfield ratio suggest P recycling, and values close to the Redfield ratio are indicative of P retention in sediments (see the Supplementary Materials for expanded discussion). C_{org}/P_{reac} ratios below the Redfield ratio can result either from additional drawdown of P in association with Fe minerals formed under ferruginous water column conditions [e.g., see (*47*, *48*)] or via extensive aerobic oxidation of organic matter under oxic conditions followed by sequestration of a proportion of the released P in association with microbial biomass (*36*).

With the exception of some oxic and ferruginous samples from the Amadeus Basin, all C_{org}/P_{org} data are elevated relative to the

Redfield ratio (Figs. 2U, 3K, and 4U). The highest C_{org}/P_{org} values correspond to euxinic/sulfidic depositional conditions (Australia max C_{org}/P_{org} = 1,397, South China max C_{org}/P_{org} = 11,293). In Australia, C_{org}/P_{reac} ratios are commonly lower than the Redfield ratio, implying sedimentary P retention (Figs. 2V and 3L). Here, C_{org}/P_{reac} ratios greater than the Redfield ratio (max C_{org}/P_{reac} = 315) are generally restricted to sulfidic/euxinic samples from Wallara-1, which also have the highest Mo_{auth} (Fig. 2, N and V, and fig. S4), consistent with the expected higher degree of P recycling under more sulfidic conditions. Together, and consistent with contemporaneous elevated $\delta^{34}S_{py}$, this suggests that the degree of bioavailable P recycling from sediments in the Australian basins was limited by the availability of sulfate to promote both microbial sulfate reduction and subsequent Fe (oxyhydr)oxide dissolution. As a result, bioavailable P recycling in Australian sections was restricted to some localized environments that were characterized by short-lived euxinia and/or where pore water sulfide was generated close to the sediment-water interface in the immediate aftermath of Sturtian deglaciation (Wallara-1; Fig. 2V).

In contrast to the Australian record, both drill cores from South China show a clear distinction in C_{org}/P_{reac} between oxic and anoxic samples (Fig. 4V). Here, oxic samples yield C_{org}/P_{reac} ratios below the Redfield ratio, indicating efficient sedimentary P retention, whereas sulfidic/euxinic samples are dominated by C_{org}/P_{reac} ratios that greatly exceed the Redfield ratio (max C_{org}/P_{reac} = 771), implying efficient recycling of bioavailable P to the water column (Fig. 4V). While P recycling under sulfidic/euxinic conditions on the shelf-slope (ZK102) was restricted to the interval immediately following Sturtian deglaciation, sulfidic pore water conditions were sufficient to permit a degree of bioavailable P recycling in the deepest basinal environment (ZK3603) throughout the non-glacial interval (Fig. 4V).

Assessing the global response of paleo-marine redox and phosphorus to Sturtian deglaciation

A recent study based on the character of P-bearing minerals and associated thermodynamic modeling of Tonian carbonates was interpreted to reflect an interval of enhanced P concentrations (*59*). However, the broader significance of this work remains unclear, and given the intense climatic perturbations to the Earth System that occurred in the interim, the coevolution of paleo-marine redox conditions and P cycling during the critical Cryogenian interval also remains unclear and warrants study. During Sturtian deglaciation, marine phosphate concentrations may have been high due to enhanced weathering rates promoted by the high surface area of sediments generated by glacial erosion (*60*). Furthermore, active rifting associated with the break-up of Rodinia resulted in the development of paleotopography that was highly weatherable (*1*).

During, and in the immediate aftermath of, Sturtian deglaciation, the primary sources of bio-available P to Australian basinal environments would have been from the chemical weathering of glacial rock flour and exposed Tonian large igneous provinces (*61*, *62*). An additional P source was contributed via bioavailable P recycling under euxinic conditions and from sulfidic sediment pore waters. This interval of relatively efficient P recycling was short-lived in Australian basins, largely as a consequence of limited water column sulfate availability. Elevated $\delta^{34}S_{py}$ in successions of both South China and Australia approach or exceed contemporaneous $\delta^{34}S_{CAS}$ (as recorded in carbonates of the Congo Craton), which likely attest to low-sulfate concentrations in global seawater at the onset of non-glacial deposition (Fig. 5B) (*15*, *63*). The interval of globally extensive sulfidic/euxinic conditions in the immediate aftermath of Sturtian deglaciation (Fig. 5, C and D) would have further reduced oceanic sulfate concentrations via widespread pyrite burial. Dissolved P would also have been supplied to the Nanhua Basin by chemical weathering of glacial rock flour and exposed crust, but our P phase association data suggest that this P input was strongly supplemented by enhanced recycling of bioavailable P from sediments under both euxinic conditions and when sulfidic pore waters existed close to the sediment-water interface (Fig. 6A). An additional source of sulfur from local hydrothermal activity may help explain sustained sulfide availability in the deep Nanhua Basin, despite generally low global marine sulfate concentrations (*38*).

The dual source of bioavailable P from primary input and redox-dependent recycling from sediments likely resulted in extensive surface ocean primary productivity and a short-lived, but globally widespread, interval of euxinic/sulfidic depositional conditions. This interval of anoxia has been recognized in multiple regional Fe speciation datasets (full compiled dataset and references in table S1) and is also consistent with reported Mo isotope data (*42*). The resulting global episode of pyrite and organic carbon burial is evident in the geochemical records of $\delta^{34}S_{py}$, TOC, and Fe_{py}/Fe_{HR}, all of which are elevated (but to a regionally variable degree) approximately coincident with the Rasthof anomaly and the subsequent rising $\delta^{13}C_{carb}$ limb approaching Cn2 (Fig. 5, A to D).

The burial of organic carbon and pyrite permits the long-term build-up of oxygen in the atmosphere (*64*). Furthermore, the solubility of oxygen in seawater is inversely related to temperature, and an increase in the O_2 saturation of surface seawater would therefore have accompanied gradual cooling in the aftermath of initial super-greenhouse conditions (Fig. 5E). Consistent with these observations, our global compilation of Fe speciation data supports a trend toward less reducing water column conditions in shallow to mid-depth environments during gradual cooling in the aftermath of the initial postglacial productivity peak (Figs. 5, C and D, and 6B and table S1). The partial and gradual oxygenation of shallow to mid-depth environments may also be consistent with the relative timing of temporary oxygenation recorded by $\delta^{238}U_{carb}$ and rare earth element patterns in the lower Taishir Formation of Mongolia (*65*, *66*), as well as trends in Fe isotope data recorded in the Datangpo Formation (*44*), and Se isotope data recorded in successions from both Australia and Laurentia (*43*).

We further interrogate the published Fe speciation record (n > 570) by using available sedimentological and lithostratigraphic information, and published facies interpretations from each depositional environment to bin data by relative depositional depth from inner shelf to slope and basin (table S1). Data trends revealed by five-point moving averages of binned Fe speciation data within the compiled age models suggest that (i) the shallowest environments experienced the fastest transition to sulfidic/euxinic conditions during the melting stage of Sturtian deglaciation, (ii) the shallowest environments experienced the fastest transition to less reducing conditions in the aftermath of Sturtian deglaciation, and (iii) the deepest basinal environment in South China remained anoxic throughout the non-glacial interval (Figs. 5, C and D, and 6B). The combined record also hints at the possibility for partial

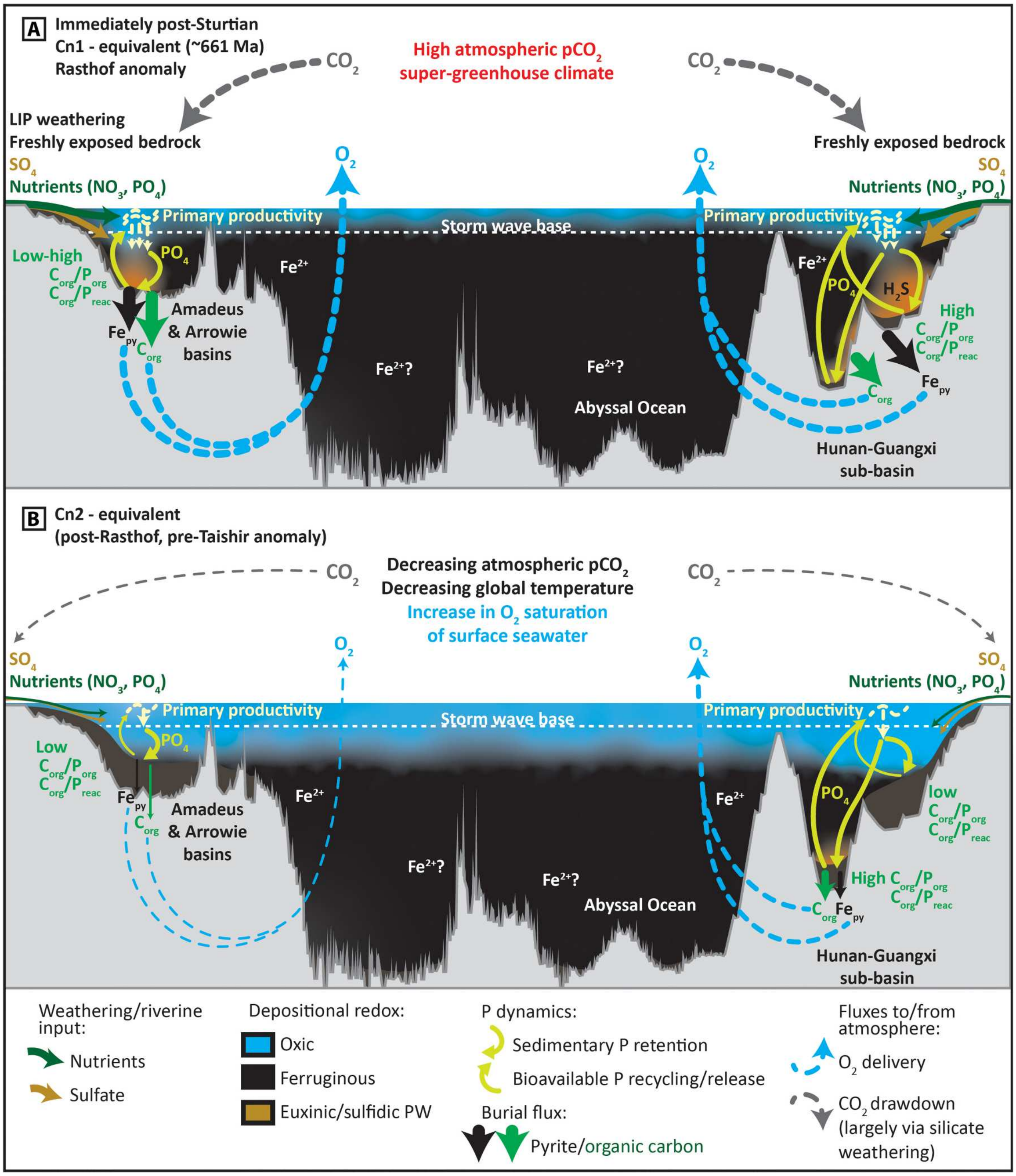

Fig. 6. Schematic dioramas representing proposed marine redox and phosphorus dynamics, in addition to burial fluxes and atmospheric response at two-time slices during the Cryogenian non-glacial interval. (**A**) In the immediate aftermath of the Sturtian deglaciation (~661 Ma), and (**B**) coincident with Cn2, between the Rasthof (Cn1) and Taishir (Cn3) $\delta^{13}C_{carb}$ anomalies (poorly constrained time interval <661 Ma to >650 Ma). The thicknesses of individual lines/arrows correspond with the relative magnitude of fluxes. The vertical scale is greatly exaggerated.

oxygenation of outer shelf-to-slope environments coincident with the Trezona (Cn5) anomaly (Fig. 5C). While numerous uncertainties remain in the relative timings of these transitions between each prescribed paleodepth, all environments from the shelf to slope clearly display the same broad transition to less reducing conditions in the interval from Cn1 to Cn2 (Fig. 5, C and D). Continued redox stratification throughout the non-glacial interval, in addition to regionally sulfidic deep marine deposition under sulfate-limited conditions, is also consistent with the available Mo, $\delta^{34}S$, and $\delta^{238}U_{carb}$ records (e.g., Fig. 5B and table S1) (*42*, *66*).

In a low-sulfate, post-Sturtian global ocean, the gradual decrease in weathering-derived nutrient and sulfate input would have reduced the shallow marine area conducive to the development of euxinic water column and sulfidic porewater conditions, leading to

enhanced sedimentary P retention in shelf environments. Global cooling and decreasing atmospheric pCO_2 may also have led to enhanced apatite authigenesis (*53*). This was accompanied by a corresponding reduction in the global areal extent of shallow seafloor conducive to bioavailable P recycling, which persisted in lower productivity deep basin environments (Fig. 6B). Even with continued continental P supply, a global decrease in sedimentary P recycling, relative to P retention, limited primary productivity and expanded the area of shallow marine oxic waters. This led to reduced organic carbon and pyrite burial on continental shelves, which may have stalled or slowed continued atmospheric oxygenation. However, positive $\delta^{13}C_{carb}$ values recorded during the Cn2 and Cn4 intervals may also imply that organic carbon burial persisted, but that the preservation of organic carbon was largely restricted to the anoxic deeper ocean and in zones of upwelling, consistent with high TOC throughout our deepest analyzed core (Fig. 4K) (*14*, *67*).

Geochemical stabilization and ecological transition during the Cryogenian non-glacial

Untangling cause and effect from the calibrated records of geochemical change and biotic first and last appearances is limited by the fragmentary nature of the paleontological record and the sensitivity of geochemical proxies to capturing ecologically meaningful changes to ecosystem habitability. Hence, we make the following observations based on our current understanding of Cryogenian non-glacial chronology and the available geochemical proxy record.

In the immediate wake of Sturtian deglaciation (Cn1), microfossil records, climate modeling, P phase association, and paleoredox proxy data suggest that a depauperate assemblage of micro-organisms inhabited warm, P-rich, redox-stratified waters, with anoxic and euxinic deeper waters recorded in multiple globally distributed environments (Figs. 1, B, D, and E, and 5). Available biomarker data show a low abundance or even the absence of eukaryotic algae in this interval, and by inference, primary production was most likely dominated by cyanobacteria (Fig. 5H) (*2*). The associated low sterane/hopane ratios (cholestane only) are recorded through ca. 240 m of strata that are coincident with, and overlie (and thereby postdate), the transition to less reducing conditions recorded by multiproxy geochemical data from the same core (BR05-DD01; Fig. 2) (*2*). This cyanobacterial-dominated assemblage therefore largely persisted throughout the gradual expansion of less reducing surface to mid-depth waters, during increasing $\delta^{13}C_{carb}$ to Cn2 (Figs. 1, B and E, and 5, C and D). While the absolute age for the first appearance of green algal steranes and putative sponge biomarkers remains uncertain, it likely postdates Cn1 (*2*) and is interpreted herein to have a maximum relative age coincident with the peak of Cn2, corresponding to the temporal position of the upper Aralka Formation recorded by core BR05-DD01 (Fig. 5H).

The delayed rise to dominance of green algae and first appearance of Demospongiae following global postglacial geochemical stabilization may reflect the continued environmental impediment of high sea surface temperatures for mesophilic algae before climate-carbon steady state (Fig. 5E and fig. S1) (*2*). Because of the dearth of globally distributed biomarker data in this interval, our age model makes the implicit assumption that there was one globally synchronous shift from red algal to green algal primary production. Following Cn3, the latter half of the Cryogenian non-glacial interval documents the first appearance of problematic macrofossils (Fig. 5H) [see, e.g., (*31*)]. These structures are found in strata that were deposited during a time in which our combined record supports generally stable redox stratification, with oxic to dysoxic (above the reduction potential conducive to the oxidation of ferrous Fe) shallow to mid-depth waters, and largely ferruginous basinal waters. The combined record also hints at the possibility for further pulsed oxygenation associated with the Trezona anomaly (Fig. 5C).

This age model framework, combined with our paleoredox and nutrient data, resolves an intuitive sequence of biotic first appearances relative to attendant climatic and geochemical change. It constrains the rise to dominance of green algae, and the first appearance of putative sponges and problematic macrofossils to an interval characterized by waning continental nutrient delivery. This interval was characterized by increased sedimentary P retention and the global expansion of less reducing, and likely more oligotrophic, inner to outer shelf environments (Figs. 1E and 5, C, D, F, and G). The timing of the maximum first appearance of putative sponges and problematic macrofossils is also consistent with the time scale of silicate weathering and CO_2 drawdown required to convert the inhospitable postglacial super-greenhouse environment to a cooler, habitable climate (Figs. 1D and 5E and fig. S1) (*24*). Thus, the gradual global stabilization of geochemical environments appears to have set the stage for an increase in biotic diversity and complexity following the Sturtian Snowball deglaciation. The time frame required for environmental stabilization may similarly be responsible for the delayed appearance of complex macrofossils (*68*) following Marinoan Snowball deglaciation.

MATERIALS AND METHODS

Global chronostratigraphic age model construction

The construction of global age models follows a hierarchical approach by first integrating litho- and $\delta^{13}C_{carb}$ chemostratigraphic information section by section into regional composite correlations based on published stratigraphic information. Individual sections are subdivided into units that are interpreted to represent broadly invariant lithofacies based on lithostratigraphy and sedimentology, and sedimentation rates are constant within (but permitted to vary between) each unit. Sedimentation rates are generally consistent with lithofacies (e.g., low rates in units of deeper marine shale and higher rates in units of shallow marine oolitic grainstone). Hiatuses are permitted at surfaces that show evidence of exposure or erosion, or where prior geochronological or geochemical information informs the presence of a cryptic hiatus. We then compare each regional composite to globally distributed datasets to produce global chemostratigraphic age frameworks.

We begin calibrating non-glacial age frameworks by constructing a chemostratigraphic scaffold of established $\delta^{13}C_{carb}$ trends from globally distributed carbonate-dominated successions of the Congo Craton, northern Namibia, the Zavkhan terrane of Mongolia, and the Brooks Range of Arctic Alaska (*4*, *8*, *11*, *12*). The resulting shape of the $\delta^{13}C_{carb}$ skeleton, and calibration of $^{87}Sr/^{86}Sr$ data from corresponding carbonate samples, is consistent with previous composite profiles developed for this interval (Fig. 1, B and C) (*4*, *8*, *13*, *22*, *69*, *70*). This skeleton profile provides a scaffold for calibration of geochemical data from interbedded carbonate-siliciclastic packages that include successions of the Centralian and Adelaide superbasins of Australia, and the Hay Creek Group and Kingston Peak Formation of Laurentia [e.g., see (*7*, *9*, *10*, *13*)]. In this intermediate step,

the ages of clastic units are constrained after visual alignment between $\delta^{13}C_{carb}$ data from interbedded carbonates and trends in the global $\delta^{13}C_{carb}$ scaffold. This approach thereby calibrates trends in a variety of proxy data from siliciclastic strata (e.g., Fe speciation and $\delta^{34}S_{py}$) relative to global trends in $\delta^{13}C_{carb}$. Last, we integrate wholly siliciclastic successions [e.g., Greenland, the Nanhua Basin, South China (*14*, *42*)] that lack any $\delta^{13}C_{carb}$ tie points but may contain radiometrically dated tuff interbeds [e.g., see (*17*)], $\delta^{13}C_{org}$, which may or may not correlate with global records of $\delta^{13}C_{carb}$, and geochemical proxy data of regional significance (e.g., Fe speciation). Given the sparsity of available radiometric constraints throughout the non-glacial interval, the absolute ages and durations of some of the resulting global geochemical trends and excursions remain uncertain (see the Supplementary Materials for expanded discussion).

Geochemical methods

To investigate the coevolution of biotic and geochemical records throughout the Cryogenian non-glacial interval, we used independent geochemical proxies that characterize regional water column and pore water paleoredox (Fe speciation and redox-sensitive trace element concentrations), as well as nutrient (P phase association) dynamics. Details of chemical extraction protocols and analytics, alongside detailed data interpretation for each core, are provided in the Supplementary Materials. All data analyzed herein are provided in tables S4 and S5.

Major and trace element concentrations of all samples were measured by inductively coupled plasma optical emission spectrometry and mass spectrometry, respectively, after quantitative HNO_3-HF-$HClO_4$ digestion. Fe speciation was performed after the established methodology of Poulton and Canfield (*71*) to extract operationally defined Fe phases, including Fe associated with carbonates (Fe_{carb}), ferric oxides (Fe_{ox}), magnetite (Fe_{mag}), and pyrite (Fe_{py}). The sum of these Fe pools constitutes the proportion of Fe that is considered highly reactive (Fe_{HR}) toward dissolved sulfide (*72*, *73*). Ratios of $Fe_{HR}/Fe_T > 0.38$ support Fe_{HR} enrichment and deposition under anoxic bottom water conditions, whereas values of <0.22 indicate deposition from oxic bottom waters (*39*). The intermediate range of 0.22 to 0.38 is regarded as equivocal because of the possibility for rapid sediment deposition or early diagenetic transformation of unsulfidized Fe_{HR} to poorly reactive sheet silicate minerals (*39*). For anoxic samples ($Fe_{HR}/Fe_T > 0.38$), the degree of sulfidation of the Fe_{HR} pool can be used to distinguish ferruginous ($Fe_{py}/Fe_{HR} < 0.7$) from euxinic ($Fe_{py}/Fe_{HR} > 0.8$) conditions, with an intermediate zone ascribed to "possible euxinia" (*39*). Recent analyses of Holocene sapropels and the euxinic Lake Cadagno indicate that $Fe_{py}/Fe_{HR} > 0.6$ may be a more suitable threshold for distinguishing ferruginous from possible euxinic conditions (*74*). We interpret possible euxinia in our study cores through a combination of $Fe_{HR}/Fe_T > 0.38$ and $Fe_{py}/Fe_{HR} > 0.6$, with additional corroborating evidence from authigenic trace element (Mo, U, and Re) enrichments (calculations and screening criteria detailed in table S2).

Selected shale samples from each core were analyzed for pyrite sulfur isotopes ($\delta^{34}S_{py}$), TOC, organic carbon isotopes ($\delta^{13}C_{org}$), and P phase associations. The P measurements use a sequential extraction method to distinguish the proportion of total P (P_{Tot}) associated with detrital apatite (P_{det}) relative to potentially bioavailable and reactive (P_{reac}) minerals, including Fe (oxyhydr)oxides (P_{Fe}), organic matter (P_{org}), and authigenic carbonate fluorapatite, biogenic apatite, and $CaCO_3$-bound P (P_{auth}) (*75*, *76*).

Supplementary Materials

This PDF file includes:
Supplementary Text
Figs. S1 to S8
Legend for table S1
Tables S2 to S5
References

Other Supplementary Material for this manuscript includes the following:
Table S1

REFERENCES AND NOTES

1. P. F. Hoffman, D. S. Abbot, Y. Ashkenazy, D. I. Benn, J. J. Brocks, P. A. Cohen, G. M. Cox, J. R. Creveling, Y. Donnadieu, D. H. Erwin, I. J. Fairchild, D. Ferreira, J. C. Goodman, G. P. Halverson, M. F. Jansen, G. Le Hir, G. D. Love, F. A. Macdonald, A. C. Maloof, C. A. Partin, G. Ramstein, B. E. J. Rose, C. V. Rose, P. M. Sadler, E. Tziperman, A. Voigt, S. G. Warren, Snowball Earth climate dynamics and Cryogenian geology-geobiology. *Sci. Adv.* **3**, e1600983 (2017).
2. J. J. Brocks, A. J. M. Jarrett, E. Sirantoine, C. Hallmann, Y. Hoshino, T. Liyanage, The rise of algae in Cryogenian oceans and the emergence of animals. *Nature* **548**, 578–581 (2017).
3. N. J. Planavsky, O. J. Rouxel, A. Bekker, S. V. Lalonde, K. O. Konhauser, C. T. Reinhard, T. W. Lyons, The evolution of the marine phosphate reservoir. *Nature* **467**, 1088–1090 (2010).
4. P. F. Hoffman, G. P. Halverson, D. P. Schrag, J. A. Higgins, E. W. Domack, F. A. Macdonald, S. B. Pruss, C. L. Blattler, P. W. Crockford, E. B. Hodgin, E. J. Bellefroid, B. W. Johnson, M. S. W. Hodgskiss, K. G. Lamothe, S. J. C. LoBianco, J. F. Busch, B. J. Howes, J. W. Greenman, L. L. Nelson, Snowballs in Africa: Sectioning a long-lived Neoproterozoic carbonate platform and its bathyal foreslope (NW Namibia). *Earth Sci. Rev.* **219**, 103616 (2021).
5. G. P. Halverson, P. F. Hoffman, D. P. Schrag, A. C. Maloof, A. H. N. Rice, Toward a Neoproterozoic composite carbon-isotope record. *Bull. Geol. Soc. Am.* **117**, 1181–1207 (2005).
6. G. P. Halverson, F. Ö. Dudás, A. C. Maloof, S. A. Bowring, Evolution of the $^{87}Sr/^{86}Sr$ composition of Neoproterozoic seawater. *Palaeogeogr. Palaeoclimatol. Palaeoecol.* **256**, 103–129 (2007).
7. J. A. Giddings, M. W. Wallace, Facies-dependent $\delta^{13}C$ variation from a Cryogenian platform margin, South Australia: Evidence for stratified Neoproterozoic oceans? *Palaeogeogr. Palaeoclimatol. Palaeoecol.* **271**, 196–214 (2009).
8. U. Bold, E. F. Smith, A. D. Rooney, S. A. Bowring, R. Buchwaldt, F. Ö. Dudás, J. Ramezani, J. L. Crowley, D. P. Schrag, F. A. Macdonald, Neoproterozoic stratigraphy of the Zavkhan terrane of Mongolia: The backbone for Cryogenian and early Ediacaran chemostratigraphic records. *Am. J. Sci.* **316**, 1–63 (2016).
9. C. Verdel, M. Campbell, Neoproterozoic carbon isotope stratigraphy of the Amadeus Basin, central Australia. *Bull. Geol. Soc. Am.* **129**, 1280–1299 (2017).
10. F. A. Macdonald, M. D. Schmitz, J. V. Strauss, G. P. Halverson, T. M. Gibson, A. Eyster, G. Cox, P. Mamrol, J. L. Crowley, Cryogenian of Yukon. *Precambrian Res.* **319**, 114–143 (2018).
11. J. V. Strauss, F. A. Macdonald, W. C. McClelland, Pre-Mississippian stratigraphy and provenance of the North Slope subterrane of Arctic Alaska I: Platformal carbonate rocks of the northeastern Brooks Range and their significance in circum-Arctic evolution, in *Circum-Arctic Structural Events: Tectonic Evolution of the Arctic Margins and Trans-Arctic Links with Adjacent Orogens*, K. Piepjohn, J. V. Strauss, L. Reinhardt, W. C. McClelland, Eds. (The Geological Society of America, 2018), Special Paper 541.
12. P. F. Hoffman, K. G. Lamothe, Seawater-buffered diagenesis, destruction of carbon isotope excursions, and the composition of DIC in Neoproterozoic oceans. *Proc. Natl. Acad. Sci. U.S.A.* **116**, 18874–18879 (2019).
13. L. L. Nelson, A. S. C. Ahm, F. A. Macdonald, J. A. Higgins, E. F. Smith, Fingerprinting local controls on the Neoproterozoic carbon cycle with the isotopic record of Cryogenian carbonates in the Panamint Range, California. *Earth Planet. Sci. Lett.* **566**, 116956 (2021).
14. X. Peng, X. K. Zhu, F. Shi, B. Yan, F. Zhang, N. Zhao, P. Peng, J. Li, D. Wang, G. A. Shields, A deep marine organic carbon reservoir in the non-glacial Cryogenian ocean (Nanhua Basin, South China) revealed by organic carbon isotopes. *Precambrian Res.* **321**, 212–220 (2019).
15. P. Gorjan, J. J. Veevers, M. R. Walter, Neoproterozoic sulfur-isotope variation in Australia and global implications. *Precambrian Res.* **100**, 151–179 (2000).

16. A. D. Rooney, F. A. Macdonald, J. V. Strauss, F. Ö. Dudás, C. Hallmann, D. Selby, Re-Os geochronology and coupled Os-Sr isotope constraints on the Sturtian snowball Earth. *Proc. Natl. Acad. Sci. U.S.A.* **111**, 51–56 (2014).
17. A. D. Rooney, C. Yang, D. J. Condon, M. Zhu, F. A. Macdonald, U-Pb and Re-Os geochronology tracks stratigraphic condensation in the Sturtian Snowball Earth aftermath. *Geology* **48**, 625–629 (2020).
18. G. M. Cox, V. Isakson, P. F. Hoffman, T. M. Gernon, M. D. Schmitz, S. Shahin, A. S. Collins, W. Preiss, M. L. Blades, R. N. Mitchell, A. Nordsvan, South Australian U-Pb zircon (CA-ID-TIMS) age supports globally synchronous Sturtian deglaciation. *Precambrian Res.* **315**, 257–263 (2018).
19. L. L. Nelson, E. F. Smith, E. B. Hodgin, J. L. Crowley, M. D. Schmitz, F. A. Macdonald, Geochronological constraints on Neoproterozoic rifting and onset of the Marinoan glaciation from the Kingston Peak Formation in Death Valley, California (USA). *Geology* **48**, 1083–1087 (2020).
20. X. Ma, J. Wang, Z. Wang, T. J. Algeo, C. Chen, Y. Cen, Q.-Z. Yin, C. Huang, L. Xu, C. Huang, D. Chen, Geochronological constraints on Cryogenian ice ages: Zircon U-Pb ages from a shelf section in South China. *Glob. Planet. Change.* **222**, 104071 (2023).
21. A. R. Prave, D. J. Condon, K. H. Hoffmann, S. Tapster, A. E. Fallick, Duration and nature of the end-Cryogenian (Marinoan) glaciation. *Geology* **44**, 631–634 (2016).
22. G. Shields, Working towards a new stratigraphic calibration scheme for the Neoproterozoic-Cambrian. *Eclogae Geol. Helv.* **92**, 221–233 (1999).
23. A.-S. C. Ahm, C. J. Bjerrum, P. F. Hoffman, F. A. Macdonald, A. C. Maloof, C. V. Rose, J. V. Strauss, J. A. Higgins, The Ca and Mg isotope record of the Cryogenian Trezona carbon isotope excursion. *Earth Planet. Sci. Lett.* **568**, 117002 (2021).
24. G. Le Hir, Y. Donnadieu, Y. Goddéris, R. T. Pierrehumbert, G. P. Halverson, M. Macouin, A. Nédélec, G. Ramstein, The Snowball Earth aftermath: Exploring the limits of continental weathering processes. *Earth Planet. Sci. Lett.* **277**, 453–463 (2009).
25. Y. Goddéris, G. Le Hir, M. Macouin, Y. Donnadieu, L. Hubert-Théou, G. Dera, M. Aretz, F. Fluteau, Z. X. Li, G. P. Halverson, Paleogeographic forcing of the strontium isotopic cycle in the Neoproterozoic. *Gondw. Res.* **42**, 151–162 (2017).
26. K. R. Moore, T. Bosak, F. Macdonald, K. Du, S. A. Newman, D. J. G. Lahr, S. B. Pruss, Pyritized Cryogenian cyanobacterial fossils from Arctic Alaska. *Palaios.* **32**, 769–778 (2017).
27. L. A. Riedman, P. M. Sadler, Global species richness record and biostratigraphic potential of early to middle Neoproterozoic eukaryote fossils. *Precambrian Res.* **319**, 6–18 (2018).
28. P. A. Cohen, M. Vizcaíno, R. P. Anderson, Oldest fossil ciliates from the Cryogenian glacial interlude reinterpreted as possible red algal spores. *Palaeontology* **63**, 941–950 (2020).
29. G. D. Love, E. Grosjean, C. Stalvies, D. A. Fike, J. P. Grotzinger, A. S. Bradley, A. E. Kelly, M. Bhatia, W. Meredith, C. E. Snape, S. A. Bowring, D. J. Condon, R. E. Summons, Fossil steroids record the appearance of Demospongiae during the Cryogenian period. *Nature* **457**, 718–721 (2009).
30. I. Bobrovskiy, J. M. Hope, B. J. Nettersheim, J. K. Volkman, C. Hallmann, J. J. Brocks, Algal origin of sponge sterane biomarkers negates the oldest evidence for animals in the rock record. *Nat. Ecol. Evol.* **5**, 165–168 (2020).
31. G. Burzynski, T. A. Dececchi, G. M. Narbonne, R. W. Dalrymple, Cryogenian *Aspidella* from northwestern Canada. *Precambrian Res.* **336**, 105507 (2020).
32. M. A. Lechte, M. W. Wallace, Sedimentary and tectonic history of the Holowilena Ironstone, a Neoproterozoic iron formation in South Australia. *Sediment. Geol.* **329**, 211–224 (2015).
33. W. V. Preiss, The Adelaide Geosyncline of South Australia and its significance in Neoproterozoic continental reconstruction. *Precambrian Res.* **100**, 21–63 (2000).
34. C. Hu, M. Zhu, Lithofacies and glacio-tectonic deformation structures of the Tiesi'ao/Dongshanfeng Formation on the Yangtze Block, South China: Implications for Sturtian Glaciation dynamics. *Palaeogeogr. Palaeoclimatol. Palaeoecol.* **538**, 109481 (2020).
35. K. K. Turekian, K. H. Wedepohl, Distribution of the elements in some major units of the Earth's crust. *Geol. Soc. Am. Bull.* **72**, 175–192 (1961).
36. E. Ingall, R. Jahnke, Evidence for enhanced phosphorus regeneration from marine sediments overlain by oxygen depleted waters. *Geochim. Cosmochim. Acta* **58**, 2571–2575 (1994).
37. D. E. Canfield, S. W. Poulton, A. H. Knoll, G. M. Narbonne, G. Ross, T. Goldberg, H. Strauss, Ferruginous conditions dominated later Neoproterozoic deep-water chemistry. *Science* **321**, 949–952 (2008).
38. P. Wang, T. J. Algeo, Q. Zhou, W. Yu, Y. Du, Y. Qin, Y. Xu, L. Yuan, W. Pan, Large accumulations of ^{34}S-enriched pyrite in a low-sulfate marine basin: The Sturtian Nanhua Basin, South China. *Precambrian Res.* **335**, 105504 (2019).
39. S. W. Poulton, D. E. Canfield, Ferruginous conditions: A dominant feature of the ocean through Earth's history. *Elements* **7**, 107–112 (2011).
40. J. Ai, N. Zhong, T. Zhang, Y. Zhang, T. Wang, S. C. George, Oceanic water chemistry evolution and its implications for post-glacial black shale formation: Insights from the Cryogenian Datangpo Formation, South China. *Chem. Geol.* **566**, 120083 (2021).
41. D. S. Hardisty, T. W. Lyons, N. Riedinger, T. T. Isson, J. D. Owens, R. C. Aller, D. M. Rye, N. J. Planavsky, C. T. Reinhard, B. C. Gill, A. L. Masterson, D. Asael, D. T. Johnston, An evaluation of sedimentary molybdenum and iron as proxies for pore fluid paleoredox conditions. *Am. J. Sci.* **318**, 527–556 (2018).
42. E. L. Scheller, A. J. Dickson, D. E. Canfield, C. Korte, K. K. Kristiansen, T. W. Dahl, Ocean redox conditions between the snowballs – Geochemical constraints from Arena Formation, East Greenland. *Precambrian Res.* **319**, 173–186 (2018).
43. P. A. E. Pogge Von Strandmann, E. E. Stüeken, T. Elliott, S. W. Poulton, C. M. Dehler, D. E. Canfield, D. C. Catling, Selenium isotope evidence for progressive oxidation of the Neoproterozoic biosphere. *Nat. Commun.* **6**, 10157 (2015).
44. F. Zhang, X. Zhu, B. Yan, B. Kendall, X. Peng, J. Li, T. J. Algeo, S. Romaniello, Oxygenation of a Cryogenian ocean (Nanhua Basin, South China) revealed by pyrite Fe isotope compositions. *Earth Planet. Sci. Lett.* **429**, 11–19 (2015).
45. T. Tyrrell, The relative influences of nitrogen and phosphorus on oceanic primary production. *Nature* **400**, 525–531 (1999).
46. C. T. Reinhard, N. J. Planavsky, B. C. Gill, K. Ozaki, L. J. Robbins, T. W. Lyons, W. W. Fischer, C. Wang, D. B. Cole, K. O. Konhauser, Evolution of the global phosphorus cycle. *Nature* **541**, 386–389 (2017).
47. R. Guilbaud, S. W. Poulton, J. Thompson, K. F. Husband, M. Zhu, Y. Zhou, G. A. Shields, T. M. Lenton, Phosphorus-limited conditions in the early Neoproterozoic ocean maintained low levels of atmospheric oxygen. *Nat. Geosci.* **13**, 296–301 (2020).
48. L. J. Alcott, B. J. W. Mills, A. Bekker, S. W. Poulton, Earth's great oxidation event facilitated by the rise of sedimentary phosphorus recycling. *Nat. Geosci.* **15**, 210–215 (2022).
49. M. A. Kipp, E. E. Stüeken, Biomass recycling and Earth's early phosphorus cycle. *Sci. Adv.* **3**, eaao4795 (2017).
50. T. A. Laakso, E. A. Sperling, D. T. Johnston, A. H. Knoll, Ediacaran reorganization of the marine phosphorus cycle. *Proc. Natl. Acad. Sci. U.S.A.* **117**, 11961–11967 (2020).
51. E. D. Ingall, R. M. Bustin, P. Van Cappellen, Influence of water column anoxia on the burial and preservation of carbon and phosphorus in marine shales. *Geochim. Cosmochim. Acta* **57**, 303–316 (1993).
52. M. D. Krom, R. A. Berner, The diagenesis of phosphorus in a nearshore marine sediment. *Geochim. Cosmochim. Acta* **45**, 207–216 (1981).
53. N. M. Papadomanolaki, W. K. Lenstra, M. Wolthers, C. P. Slomp, Enhanced phosphorus recycling during past oceanic anoxia amplified by low rates of apatite authigenesis. *Sci. Adv.* **8**, eabn2370 (2022).
54. P. Van Cappellen, E. D. Ingall, Benthic phosphorus regeneration, net primary production and ocean anoxia. *Paleoceanography* **9**, 677–692 (1994).
55. K. C. Ruttenberg, R. A. Berner, Authigenic apatite formation and burial in sediments from non-upwelling, continental margin environments. *Geochim. Cosmochim. Acta* **57**, 991–1007 (1993).
56. Y. Xiong, R. Guilbaud, C. L. Peacock, R. P. Cox, D. E. Canfield, M. D. Krom, S. W. Poulton, Phosphorus cycling in Lake Cadagno, Switzerland: A low sulfate euxinic ocean analogue. *Geochim. Cosmochim. Acta* **251**, 116–135 (2019).
57. C. P. Slomp, S. J. Van Der Gaast, W. Van Raaphorst, Phosphorus binding by poorly crystalline iron oxides in North Sea sediments. *Mar. Chem.* **52**, 55–73 (1996).
58. C. P. Slomp, E. H. G. Epping, W. Helder, W. Van Raaphorst, A key role for iron-bound phosphorus in authigenic apatite formation in North Atlantic continental platform sediments. *J. Mar. Res.* **54**, 1179–1205 (1996).
59. S. Roest-Ellis, J. A. Richardson, B. L. Phillips, A. Mehra, S. M. Webb, P. A. Cohen, J. V. Strauss, N. J. Tosca, Tonian carbonates record phosphate-rich shallow seas. *Geochem. Geophys. Geosystems* **24**, e2023GC010974 (2023).
60. M. Tranter, M. J. Sharp, H. R. Lamb, G. H. Brown, B. P. Hubbard, I. C. Willis, Geochemical weathering at the bed of Haut Glacier d'Arolla, Switzerland—A new model. *Hydrol. Process.* **16**, 959–993 (2002).
61. G. M. Cox, T. W. Lyons, R. N. Mitchell, D. Hasterok, M. Gard, Linking the rise of atmospheric oxygen to growth of the continental phosphorus inventory. *Earth Planet. Sci. Lett.* **489**, 28–36 (2018).
62. R. E. Ernst, D. P. G. Bond, S.-H. Zhang, K. L. Buchan, S. E. Grasby, N. Youbi, H. El Bilali, A. Bekker, L. Doucet, Large igneous province record through time and implications for secular environmental changes and geological time-scale boundaries, in *Large Igneous Provinces: A Driver of Global Environmental and Biotic Changes*, R. E. Ernst, A. J. Dickson, A. Bekker, Eds., Geophysical Monograph 255 (AGU, 2021), pp. 3–26.
63. M. T. Hurtgen, M. A. Arthur, G. P. Halverson, Neoproterozoic sulfur isotopes, the evolution of microbial sulfur species, and the burial efficiency of sulfide as sedimentary pyrite. *Geology* **33**, 41–44 (2005).
64. D. E. Canfield, The early history of atmospheric oxygen: Homage to Robert M. Garrels. *Annu. Rev. Earth Planet. Sci.* **33**, 1–36 (2005).

65. G. Shields, P. Stille, Stratigraphic trends in cerium anomaly in authigenic marine carbonates and phosphates: Diagenetic alteration or seawater signals? *Mineral. Mag.* **62A**, 1387–1388 (1998).
66. K. V. Lau, F. A. Macdonald, K. Maher, J. L. Payne, Uranium isotope evidence for temporary ocean oxygenation in the aftermath of the Sturtian Snowball Earth. *Earth Planet. Sci. Lett.* **458**, 282–292 (2017).
67. G. A. Shields, M. D. Brasier, P. Stille, D. Dorjnamjaa, Factors contributing to high $\delta^{13}C$ values in Cryogenian limestones of western Mongolia. *Earth Planet. Sci. Lett.* **196**, 99–111 (2002).
68. C. Yang, A. D. Rooney, D. J. Condon, X.-H. Li, D. V. Grazhdankin, F. T. Bowyer, C. Hu, F. Macdonald, M. Zhu, The tempo of Ediacaran evolution. *Sci. Adv.* **7**, eabi9643 (2021).
69. A. J. Kaufman, A. H. Knoll, G. M. Narbonne, Isotopes, ice ages, and terminal Proterozoic earth history. *Proc. Natl. Acad. Sci. U.S.A.* **94**, 6600–6605 (1997).
70. D. M. McKirdy, J. M. Burgess, N. M. Lemon, X. Yu, A. M. Cooper, V. A. Gostin, R. J. F. Jenkins, R. A. Both, A chemostratigraphic overview of the late Cryogenian interglacial sequence in the Adelaide Fold-Thrust Belt, South Australia. *Precambrian Res.* **106**, 149–186 (2001).
71. S. W. Poulton, D. E. Canfield, Development of a sequential extraction procedure for iron: Implications for iron partitioning in continentally derived particulates. *Chem. Geol.* **214**, 209–221 (2005).
72. R. Raiswell, D. E. Canfield, Sources of iron for pyrite formation in marine sediments. *Am. J. Sci.* **298**, 219–245 (1998).
73. S. W. Poulton, P. W. Fralick, D. E. Canfield, The transition to a sulphidic ocean ~1.84 billion years ago. *Nature* **431**, 173–177 (2004).
74. A. Benkovitz, A. Matthews, N. Teutsch, S. W. Poulton, M. Bar-Matthews, A. Almogi-Labin, Tracing water column euxinia in Eastern Mediterranean Sapropels S5 and S7. *Chem. Geol.* **545**, 119627 (2020).
75. K. C. Ruttenberg, Development of a sequential extraction method for different forms of phosphorus in marine sediments. *Limnol. Oceanogr.* **37**, 1460–1482 (1992).
76. J. Thompson, S. W. Poulton, R. Guilbaud, K. A. Doyle, S. Reid, M. D. Krom, Development of a modified SEDEX phosphorus speciation method for ancient rocks and modern iron-rich sediments. *Chem. Geol.* **524**, 383–393 (2019).
77. A. S. Merdith, S. E. Williams, A. S. Collins, M. G. Tetley, J. A. Mulder, M. L. Blades, A. Young, S. Armistead, J. Cannon, S. Zahirovic, R. D. Müller, Extending full-plate tectonic models into deep time: Linking the Neoproterozoic and Phanerozoic. *Earth-Science Rev.* **214**, 103477 (2021).
78. D. I. Benn, G. Le Hir, H. Bao, Y. Donnadieu, C. Dumas, E. J. Fleming, M. J. Hambrey, E. A. McMillan, M. S. Petronis, G. Ramstein, C. T. E. Stevenson, P. M. Wynn, I. J. Fairchild, Orbitally forced ice sheet fluctuations during the Marinoan Snowball Earth glaciation. *Nat. Geosci.* **8**, 704–707 (2015).
79. K. Grey, C. R. Calver, Correlating the Ediacaran of Australia. *Geol. Soc. Spec. Publ.* **286**, 115–135 (2007).
80. J. C. Lloyd, M. L. Blades, J. W. Counts, A. S. Collins, K. J. Amos, B. P. Wade, J. W. Halls, S. Hore, A. L. Ball, S. Shahin, M. Drabsch, Neoproterozoic geochronology and provenance of the Adelaide Superbasin. *Precambrian Res.* **350**, 105849 (2020).
81. H. J. Allen, K. Grey, P. W. Haines, C. J. Edgoose, V. J. Normington, *The Cryogenian Aralka Formation, Amadeus Basin: A Basinwide Biostratigraphic Correlation* (Geological Survey of Western Australia, 2018).
82. K. Grey, H.-J. Allen, A. Hill, P. W. Haines, Neoproterozoic biostratigraphy of the Amadeus Basin, in *Proceedings of the Petroleum Exploration Society of Australia (PESA); Central Australian Basins Symposium III, Alice Springs, Northern Territory, 16–17 July*, G. J. Ambrose, J. Scott, Eds. (PESA: Special Publication, 2012), p. 18.
83. M. W. Wallace, A. v. S. Hood, E. M. S. Woon, J. A. Giddings, T. A. Fromhold, The Cryogenian Balcanoona reef complexes of the Northern Flinders Ranges: Implications for Neoproterozoic ocean chemistry. *Palaeogeogr. Palaeoclimatol. Palaeoecol.* **417**, 320–336 (2015).
84. B. O'Connell, M. W. Wallace, A. v. S. Hood, M. A. Lechte, N. J. Planavsky, Iron-rich carbonate tidal deposits, Angepena Formation, South Australia: A redox-stratified Cryogenian basin. *Precambrian Res.* **342**, 105668 (2020).
85. L. A. Riedman, S. M. Porter, G. P. Halverson, M. T. Hurtgen, C. K. Junium, Organic-walled microfossil assemblages from glacial and interglacial Neoproterozoic units of Australia and Svalbard. *Geology* **42**, 1011–1014 (2014).
86. L. Yin, X. Yuan, Radiation of Meso-Neoproterozoic and Early Cambrian protists inferred from the microfossil record of China. *Palaeogeogr. Palaeoclimatol. Palaeoecol.* **254**, 350–361 (2007).
87. J. Wang, Z. X. Li, History of Neoproterozoic rift basins in South China: Implications for Rodinia break-up. *Precambrian Res.* **122**, 141–158 (2003).
88. S. H. Zhang, D. A. D. Evans, H. Y. Li, H. C. Wu, G. Q. Jiang, J. Dong, Q. L. Zhao, T. D. Raub, T. S. Yang, Paleomagnetism of the late Cryogenian Nantuo Formation and paleogeographic implications for the South China Block. *J. Asian Earth Sci.* **72**, 164–177 (2013).
89. A. S. Merdith, A. S. Collins, S. E. Williams, S. Pisarevsky, J. D. Foden, D. B. Archibald, M. L. Blades, B. L. Alessio, S. Armistead, D. Plavsa, C. Clark, R. D. Müller, A full-plate global reconstruction of the Neoproterozoic. *Gondw. Res.* **50**, 84–134 (2017).
90. Z. Lan, X. Li, M. Zhu, Z. Q. Chen, Q. Zhang, Q. Li, D. Lu, Y. Liu, G. Tang, A rapid and synchronous initiation of the wide spread Cryogenian glaciations. *Precambrian Res.* **255**, 401–411 (2014).
91. Z. Lan, X. H. Li, Q. Zhang, Q. L. Li, Global synchronous initiation of the 2nd episode of Sturtian glaciation: SIMS zircon U-Pb and O isotope evidence from the Jiangkou Group, South China. *Precambrian Res.* **267**, 28–38 (2015).
92. W. Wei, R. Frei, R. Klaebe, D. Li, G. Y. Wei, H. F. Ling, Redox condition in the Nanhua Basin during the waning of the Sturtian glaciation: A chromium-isotope perspective. *Precambrian Res.* **319**, 198–210 (2018).
93. L. Feng, J. Huang, D. Lu, Q. Zhang, Major and trace element geochemistry of the Neoproterozoic syn-glacial Fulu iron formation, South China. *Geol. Mag.* **154**, 1371–1380 (2017).
94. C. Zhou, M. H. Huyskens, X. Lang, S. Xiao, Q. Z. Yin, Calibrating the terminations of Cryogenian global glaciations. *Geology* **47**, 251–254 (2019).
95. A. D. Rooney, J. V. Strauss, A. D. Brandon, F. A. Macdonald, A Cryogenian chronology: Two long-lasting synchronous Neoproterozoic glaciations. *Geology* **43**, 459–462 (2015).
96. X. Bao, S. Zhang, G. Jiang, H. Wu, H. Li, X. Wang, Z. An, T. Yang, Cyclostratigraphic constraints on the duration of the Datangpo Formation and the onset age of the Nantuo (Marinoan) glaciation in South China. *Earth Planet. Sci. Lett.* **483**, 52–63 (2018).
97. L. Yin, Microfossils from late Proterozoic manganese ore deposits in western Hunan Province and eastern Guizhou Province, South China. *Sci. China Ser. B.* **8**, 861–866 (1990).
98. D. E. Canfield, R. Raiswell, J. T. Westrich, C. M. Reaves, R. A. Berner, The use of chromium reduction in the analysis of reduced inorganic sulfur in sediments and shales. *Chem. Geol.* **54**, 149–155 (1986).
99. L. J. Alcott, A. J. Krause, E. U. Hammarlund, C. J. Bjerrum, F. Scholz, Y. Xiong, A. J. Hobson, L. Neve, B. W. Mills, C. März, B. Schnetger, A. Bekker, S. W. Poulton, Development of iron speciation reference materials for palaeoredox analysis. *Geostand. Geoanalytical Res.* **44**, 581–591 (2020).
100. V. Pasquier, D. A. Fike, S. Révillon, I. Halevy, A global reassessment of the controls on iron speciation in modern sediments and sedimentary rocks: A dominant role for diagenesis. *Geochim. Cosmochim. Acta* **335**, 211–230 (2022).
101. M. O. Clarkson, S. W. Poulton, R. Guilbaud, R. A. Wood, Assessing the utility of Fe/Al and Fe-speciation to record water column redox conditions in carbonate-rich sediments. *Chem. Geol.* **382**, 111–122 (2014).
102. D. E. Canfield, T. W. Lyons, R. Raiswell, A model for iron deposition to euxinic black sea sediments. *Am. J. Sci.* **296**, 818–834 (1996).
103. G.-Y. Wei, T. Chen, S. W. Poulton, Y.-B. Lin, T. He, X. Shi, J. Chen, H. Li, S. Qiao, J. Liu, D. Li, H.-F. Ling, A chemical weathering control on the delivery of particulate iron to the continental shelf. *Geochim. Cosmochim. Acta* **308**, 204–216 (2021).
104. S. W. Poulton, R. Raiswell, The low-temperature geochemical cycle of iron: From continental fluxes to marine sediment deposition. *Am. J. Sci.* **302**, 774–805 (2002).
105. J. L. Kirschvink, Late Proterozoic low-latitude global glaciation: The snowball Earth, in *The Proterozoic Biosphere: A Multidisciplinary Study*, J. W. Schopf, C. Klein, D. Des Maris, Eds. (Cambridge Univ. Press, 1992), pp. 51–52.
106. P. F. Hoffman, A. J. Kaufman, G. P. Halverson, D. P. Schrag, A Neoproterozoic Snowball Earth. *Science* **281**, 1342–1346 (1998).
107. J. Ai, N. Zhong, S. C. George, Y. Zhang, L. Yao, T. Wang, Evolution of paleo-weathering during the late Neoproterozoic in South China: Implications for paleoclimatic conditions and organic carbon burial. *Palaeogeogr. Palaeoclimatol. Palaeoecol.* **555**, 109843 (2020).
108. P. Wang, Y. Du, W. Yu, T. J. Algeo, Q. Zhou, Y. Xu, L. Qi, L. Yun, W. Pan, The chemical index of alteration (CIA) as a proxy for climate change during glacial-interglacial transitions in Earth history. *Earth-Science Rev.* **201**, 103032 (2020).
109. G. Zhu, T. Li, Z. Zhang, K. Zhao, H. Song, P. Wang, H. Yan, H. Song, Nitrogen isotope evidence for oxygenated upper ocean during the Cryogenian interglacial period. *Chem. Geol.* **604**, 120929 (2022).
110. R. Raiswell, R. Newton, S. H. Bottrell, P. M. Coburn, D. E. G. Briggs, D. P. G. Bond, S. W. Poulton, Turbidite depositional influences on the diagenesis of Beecher's Trilobite Bed and the Hunsrück Slate; sites of soft tissue pyritization. *Am. J. Sci.* **308**, 105–129 (2008).
111. R. Raiswell, R. Newton, P. B. Wignall, An indicator of water-column anoxia: Resolution of biofacies variations in the Kimmeridge clay (Upper Jurassic, UK). *J. Sediment. Res.* **71**, 286–294 (2001).
112. S. W. Poulton, *The Iron Speciation Paleoredox Proxy* (Cambridge Univ. Press, 2021).
113. R. Raiswell, D. S. Hardisty, T. W. Lyons, D. E. Canfield, J. D. Owens, N. J. Planavsky, S. W. Poulton, C. T. Reinhard, The iron paleoredox proxies: A guide to the pitfalls, problems and proper practice. *Am. J. Sci.* **318**, 491–526 (2018).

114. D. B. Cole, S. Zhang, N. J. Planavsky, A new estimate of detrital redox-sensitive metal concentrations and variability in fluxes to marine sediments. *Geochim. Cosmochim. Acta* **215**, 337–353 (2017).
115. R. L. Rudnick, S. Gao, Composition of the continental crust, in *The crust*, R. L. Rudnick, H. D. Holland, K. K. Turekian, Eds. (Elsevier, 2003), pp. 1–64.
116. N. Tribovillard, T. J. Algeo, T. Lyons, A. Riboulleau, Trace metals as paleoredox and paleoproductivity proxies: An update. *Chem. Geol.* **232**, 12–32 (2006).
117. T. J. Algeo, N. Tribovillard, Environmental analysis of paleoceanographic systems based on molybdenum-uranium covariation. *Chem. Geol.* **268**, 211–225 (2009).
118. M. Kunzmann, G. P. Halverson, C. Scott, W. G. Minarik, B. A. Wing, Geochemistry of Neoproterozoic black shales from Svalbard: Implications for oceanic redox conditions spanning Cryogenian glaciations. *Chem. Geol.* **417**, 383–393 (2015).
119. A. I. Sheen, B. Kendall, C. T. Reinhard, R. A. Creaser, T. W. Lyons, A. Bekker, S. W. Poulton, A. D. Anbar, A model for the oceanic mass balance of rhenium and implications for the extent of Proterozoic ocean anoxia. *Geochim. Cosmochim. Acta* **227**, 75–95 (2018).
120. J. L. Morford, S. Emerson, The geochemistry of redox sensitive trace metals in sediments. *Geochim. Cosmochim. Acta* **63**, 1735–1750 (1999).
121. J. Crusius, S. Calvert, T. Pedersen, D. Sage, Rhenium and molybdenum enrichments in sediments as indicators of oxic, suboxic and sulfidic conditions of deposition. *Earth Planet. Sci. Lett.* **145**, 65–78 (1996).
122. J. L. Morford, S. R. Emerson, E. J. Breckel, S. H. Kim, Diagenesis of oxyanions (V, U, Re, and Mo) in pore waters and sediments from a continental margin. *Geochim. Cosmochim. Acta* **69**, 5021–5032 (2005).
123. C. A. Partin, A. Bekker, N. J. Planavsky, C. T. Scott, B. C. Gill, C. Li, V. Podkovyrov, A. Maslov, K. O. Konhauser, S. V. Lalonde, G. D. Love, S. W. Poulton, T. W. Lyons, Large-scale fluctuations in Precambrian atmospheric and oceanic oxygen levels from the record of U in shales. *Earth Planet. Sci. Lett.* **369–370**, 284–293 (2013).
124. C. T. Reinhard, N. J. Planavsky, L. J. Robbins, C. A. Partin, B. C. Gill, S. V. Lalonde, A. Bekker, K. O. Konhauser, T. W. Lyons, Proterozoic ocean redox and biogeochemical stasis. *Proc. Natl. Acad. Sci. U.S.A.* **110**, 5357–5362 (2013).
125. N. Tribovillard, T. J. Algeo, F. Baudin, A. Riboulleau, Analysis of marine environmental conditions based on molybdenum-uranium covariation—Applications to Mesozoic paleoceanography. *Chem. Geol.* **324–325**, 46–58 (2012).
126. C. E. Barnes, J. K. Cochran, Uranium removal in oceanic sediments and the oceanic U balance. *Earth Planet. Sci. Lett.* **97**, 94–101 (1990).
127. G. P. Klinkhammer, M. R. Palmer, Uranium in the oceans: Where it goes and why. *Geochim. Cosmochim. Acta* **55**, 1799–1806 (1991).
128. L. Olson, K. A. Quinn, M. G. Siebecker, G. W. Luther, D. Hastings, J. L. Morford, Trace metal diagenesis in sulfidic sediments: Insights from Chesapeake Bay. *Chem. Geol.* **452**, 47–59 (2017).
129. G. R. Helz, C. V. Miller, J. M. Charnock, J. F. W. Mosselmans, R. A. D. Pattrick, C. D. Garner, D. J. Vaughan, Mechanism of molybdenum removal from the sea and its concentration in black shales : EXAFS evidence. *Geochim. Cosmochim. Acta* **60**, 3631–3642 (1996).
130. T. J. Algeo, T. W. Lyons, Mo–total organic carbon covariation in modern anoxic marine environments: Implications for analysis of paleoredox and paleohydrographic conditions. *Paleoceanography* **21**, PA1016 (2006).
131. C. Scott, T. W. Lyons, A. Bekker, Y. Shen, S. W. Poulton, X. Chu, A. D. Anbar, Tracing the stepwise oxygenation of the Proterozoic ocean. *Nature* **452**, 456–459 (2008).
132. J. R. Creveling, D. T. Johnston, S. W. Poulton, B. Kotrc, C. März, D. P. Schrag, A. H. Knoll, Phosphorus sources for phosphatic Cambrian carbonates. *Bull. Geol. Soc. Am.* **126**, 145–163 (2014).
133. F. Horton, Did phosphorus derived from the weathering of large igneous provinces fertilize the Neoproterozoic ocean? *Geochem. Geophys. Geosystems* **16**, 1723–1738 (2015).
134. S. Sharoni, I. Halevy, Geologic controls on phytoplankton elemental composition. *Proc. Natl. Acad. Sci. U.S.A.* **119**, e2113263118 (2022).
135. N. J. Planavsky, The elements of marine life. *Nat. Geosci.* **7**, 855–856 (2014).
136. W. Yu, T. J. Algeo, Y. Du, Q. Zhou, P. Wang, Y. Xu, L. Yuan, W. Pan, Newly discovered Sturtian cap carbonate in the Nanhua Basin, South China. *Precambrian Res.* **293**, 112–130 (2017).
137. C. Zhou, R. Tucker, S. Xiao, Z. Peng, X. Yuan, Z. Chen, New constraints on the ages of Neoproterozoic glaciations in south China. *Geology* **32**, 437–440 (2004).
138. D. M. McKirdy, J. M. Burgess, N. M. Lemon, X. Yu, A. M. Cooper, V. A. Gostin, R. J. F. Jenkins, R. A. Both, A chemostratigraphic overview of the late Cryogenian interglacial sequence in the Adelaide Fold-Thrust Belt, South Australia. *Precambrian Res.* **106**, 149–186 (2001).
139. M. Ader, M. Macouin, R. I. F. Trindade, M.-H. Hadrien, Z. Yang, Z. Sun, J. Besse, A multilayered water column in the Ediacaran Yangtze platform? Insights from carbonate and organic matter paired $\delta^{13}C$. *Earth Planet. Sci. Lett.* **288**, 213–227 (2009).
140. M. Ader, P. Sansjofre, G. P. Halverson, V. Busigny, R. I. F. Trindade, M. Kunzmann, A. C. R. Nogueira, Ocean redox structure across the Late Neoproterozoic Oxygenation Event: A nitrogen isotope perspective. *Earth Planet. Sci. Lett.* **396**, 1–13 (2014).
141. R. P. Anderson, I. J. Fairchild, N. J. Tosca, A. H. Knoll, Microstructures in metasedimentary rocks from the Neoproterozoic Bonahaven Formation, Scotland: Microconcretions, impact spherules, or microfossils? *Precambrian Res.* **233**, 59–72 (2013).
142. M. Bau, P. Dulski, Distribution of yttrium and rare-earth elements in the Penge and Kuruman iron-formations, Transvaal Supergroup, South Africa. *Precambrian Res.* **79**, 37–55 (1986).
143. J. Bertrand-Sarfati, A. Siedlecka, Columnar stromatolites of the terminal precambrian porsanger dolomite and grasdal formation of Finnmark, North Norway. *Norsk Geologisk Tidsskrift* **60**, 1–27 (1980).
144. T. Bosak, D. J. G. Lahr, S. B. Pruss, F. A. Macdonald, L. Dalton, E. Matys, Agglutinated tests in post-Sturtian cap carbonates of Namibia and Mongolia. *Earth Planet. Sci. Lett.* **308**, 29–40 (2011).
145. T. Bosak, F. Macdonald, D. Lahr, E. Matys, Putative cryogenian ciliates from Mongolia. *Geology* **39**, 1123–1126 (2011).
146. T. Bosak, D. J. G. Lahr, S. B. Pruss, F. A. Macdonald, A. J. Gooday, L. Dalton, E. D. Matys, Possible early foraminiferans in post-Sturtian (716-635 Ma) cap carbonates. *Geology* **40**, 67–70 (2012).
147. C. K. B. Brain, A. R. Prave, K. H. Hoffmann, A. E. Fallick, A. Botha, D. A. Herd, C. Sturrock, I. Young, D. J. Condon, S. G. Allison, The first animals: Ca. 760-million-year-old sponge-like fossils from Namibia. *S. Afr. J. Sci.* **108**, 1–8 (2012).
148. M. D. Brasier, G. Shields, V. N. Kuleshov, E. A. Zhegallo, Integrated chemo- and biostratigraphic calibration of early animal evolution: Neoproterozoic-early Cambrian of southwest Mongolia. *Geol. Mag.* **133**, 445–485 (1996).
149. M. D. Brasier, G. Shields, Neoproterozoic chemostratigraphy and correlation of the Port Askaig glaciation, Dalradian Supergroup of Scotland. *J. Geol. Soc. London* **157**, 909–914 (2000).
150. C. Calver, Isotope stratigraphy of the Neoproterozoic Togari group, Tasmania. *Aust. J. Earth Sci.* **45**, 865–874 (1998).
151. X. Chen, S. Romaniello, A. D. Hermann, D. S. Hardisty, B. C. Gill, A. D. Anbar, Diagenetic effects on uranium isotope fractionation in carbonate sediments from the Bahamas. *Geochim. Cosmochim. Acta* **237**, 294–311 (2018).
152. M. Cheng, C. Li, X. Chen, L. Zhou, T. J. Algeo, H.-F. Ling, L.-J. Feng, C.-S. Jin, Delayed Neoproterozoic ocean oxygenation: Evidence from Mo isotopes of the Cryogenian Datangpo Formation. *Precambrian Res.* **319**, 187–197 (2018).
153. P. A. Cohen, F. A. Macdonald, S. Pruss, E. Matys, T. Bosak, Fossils of putative marine algae from the Cryogenian glacial interlude of Mongolia. *Palaios* **30**, 238–247 (2015).
154. L. A. Dalton, T. Bosak, F. A. Macdonald, D. J. G. Lahr, S. B. Pruss, Preservational and morphological variability of assemblages of agglutinated eukaryotes in Cryogenian cap carbonates of northern Namibia. *Palaios* **28**, 67–79 (2013).
155. H. J. W. de Baar, M. P. Bacon, P. G. Brewer, K. W. Bruland, Rare earth elements in the Pacific and Atlantic Oceans. *Geochim. Cosmochim. Acta* **49**, 1943–1959 (1985).
156. I. J. Fairchild, P. Bonnard, T. Davies, E. J. Fleming, N. Grassineau, G. P. Halverson, M. J. Hambrey, E. M. McMillan, E. McKay, I. J. Parkinson, C. T. E. Stevenson, The late Cryogenian warm interval, NE Svalbard: Chemostratigraphy and genesis. *Precambrian Res.* **281**, 128–154 (2016).
157. L.-J. Feng, X.-L. Chu, J. Huang, Q.-R. Zhang, H.-J. Chang, Reconstruction of paleo-redox conditions and early sulfur cycling during deposition of the Cryogenian Datangpo Formation in South China. *Gondw. Res.* **18**, 632–637 (2010).
158. G. P. Halverson, P. F. Hoffman, D. P. Schrag, A major perturbation of the carbon cycle before the Ghaub glaciation (Neoproterozoic) in Namibia: Prelude to snowball Earth? *Geochem. Geophys. Geosystems* **3**, 1–24 (2002).
159. P. F. Hoffman, D. P. Schrag, The Snowball Earth hypothesis: Testing the limits of global change. *Terra Nova* **14**, 129–155 (2002).
160. P. F. Hoffman, G. P. Halverson, E. W. Domack, A. C. Maloof, N. L. Swanson-Hysell, G. M. Cox, Cryogenian glaciations on the southern tropical paleomargin of Laurentia (NE Svalbard and East Greenland), and a primary origin for the upper Russoya (Islay) carbon isotope excursion. *Precambrian Res.* **206-207**, 137–158 (2012).
161. H. J. Hofmann, G. M. Narbonne, J. D. Aitken, Ediacaran remains from intertillite beds in northwestern Canada. *Geology* **18**, 1199–1202 (1990).
162. A. v. S. Hood, M. W. Wallace, Extreme ocean anoxia during the Late Cryogenian recorded in reefal carbonates of Southern Australia. *Precambrian Res.* **261**, 96–111 (2015).
163. M. T. Hurtgen, M. A. Arthur, N. S. Suits, A. J. Kaufman, The sulfur isotopic composition of Neoproterozoic seawater sulfate: Implications for a snowball Earth? *Earth Planet. Sci. Lett.* **203**, 413–429 (2002).
164. D. T. Johnston, F. A. Macdonald, B. C. Gill, P. F. Hoffman, D. P. Schrag, Uncovering the Neoproterozoic carbon cycle. *Nature* **483**, 320–324 (2012).
165. M. Kunzmann, T. H. Bui, P. W. Crockford, G. P. Halverson, C. Scott, T. W. Lyons, B. A. Wing, Bacterial sulfur disproportionation constrains timing of Neoproterozoic oxygenation. *Geology* **45**, 207–210 (2017).

166. M. G. Lawrence, A. Greig, K. D. Collerson, B. S. Kamber, Rare earth element and yttrium variability in South East Queensland waterways. *Aquat. Geochem.* **12**, 39–72 (2006).
167. C. Li, G. D. Love, T. W. Lyons, C. T. Scott, L. Feng, J. Huang, H. Chang, Q. Zhang, X. Chu, Evidence for a redox stratified Cryogenian marine basin, Datangpo Formation, South China. *Earth Planet. Sci. Lett.* **331-332**, 246–256 (2012).
168. H. F. Ling, X. Chen, D. Li, D. Wang, G. A. Shields-Zhou, M. Zhu, Cerium anomaly variations in Ediacaran-earliest Cambrian carbonates from the Yangtze Gorges area, South China: Implications for oxygenation of coeval shallow seawater. *Precambrian Res.* **225**, 110–127 (2013).
169. F. A. Macdonald, W. C. McClelland, D. P. Schrag, W. P. Macdonald, Neoproterozoic glaciation on a carbonate platform margin in Arctic Alaska and the origin of the North Slope subterrane. *GSA Bull.* **121**, 448–473 (2009).
170. F. A. Macdonald, J. V. Strauss, E. A. Sperling, G. P. Halverson, G. M. Narbonne, D. T. Johnston, M. Kunzmann, D. P. Schrag, J. A. Higgins, The stratigraphic relationship between the Shuram carbon isotope excursion, the oxygenation of Neoproterozoic oceans, and the first appearance of the Ediacara biota and bilaterian trace fossils in northwestern Canada. *Chem. Geol.* **362**, 250–272 (2013).
171. A. C. Maloof, C. V. Rose, R. Beach, B. M. Samuels, C. C. Calmet, D. H. Erwin, G. R. Poirier, N. Yao, F. J. Simons, Possible animal-body fossils in pre-Marinoan limestones from South Australia. *Nat. Geosci.* **3**, 653–659 (2010).
172. S. B. McLennan, Rare earth elements in sedimentary rocks. Influence of provenance and sedimentary processes, in *Geochemistry and Mineralogy of the Rare Earth Elements*, B. R. Lipin, G. A. McKay, Eds., (Mineralogical Society of America, 1989), Special Paper, pp. 169–200.
173. K. R. Moore, T. Bosak, F. A. Macdonald, D. J. G. Lahr, S. Newman, C. Settens, S. B. Pruss, Biologically agglutinated eukaryotic microfossil from Cryogenian cap carbonates. *Geobiology* **15**, 499–515 (2017).
174. A. R. Prave, A. E. Fallick, C. W. Thomas, C. M. Graham, A composite C-isotope profile for the Neoproterozoic Dalradian Supergroup of Scotland and Ireland. *J. Geol. Soc. London* **166**, 845–857 (2009).
175. M. E. Raaben, V. V. Lyubtsov, A. A. Predovsky, Correlation of stromatolitic formations of northern Norway (Finnmark) and northwestern Russian (Kildin Island and Kanin Peninsula). *Norges Geologiske Undersøkelse Special Publication* **7**, 233–246 (1995).
176. A. S. Rodler, R. Frei, C. Gaucher, C. Korte, S. A. Rosing, G. J. B. Germs, Multiproxy isotope constraints on ocean compositional changes across the late Neoproterozoic Ghaub glaciation, Otavi Group, Namibia. *Precambrian Res.* **298**, 306–324 (2017).
177. E. A. Sperling, C. J. Wolock, A. S. Morgan, B. C. Gill, M. Kunzmann, G. P. Halverson, F. A. Macdonald, A. H. Knoll, D. T. Johnston, Statistical analysis of iron geochemical data suggests limited late Proterozoic oxygenation. *Nature* **523**, 451–454 (2015).
178. E. A. Sperling, C. Carbone, J. V. Strauss, D. T. Johnston, G. M. Narbonne, F. A. Macdonald, Oxygen, facies, and secular controls on the appearance of Cryogenian and Ediacaran body and trace fossils in the Mackenzie Mountains of northwestern Canada. *GSA Bull.* **128**, 558–575 (2016).
179. N. L. Swanson-Hysell, C. V. Rose, C. C. Calmet, G. P. Halverson, M. T. Hurtgen, A. C. Maloof, Cryogenian glaciation and the onset of carbon-isotope decoupling. *Science* **328**, 608–611 (2010).
180. J. E. Tesdal, E. D. Galbraith, M. Kienast, Nitrogen isotopes in bulk marine sediment: Linking seafloor observations with subseafloor records. *Biogeosciences* **10**, 101–118 (2013).
181. G. Vidal, Late Precambrian acritarchs from the Eleonore Bay Group and Tillite Group in East Greenland: A preliminary report. *Grønlands Geologiske Undersøgelse Rapport* **78**, 1–19 (1976).
182. G. Vidal, Acritarchs from the Upper Proterozoic and Lower Cambrian of East Greenland. *Grønlands Geologiske Undersøgelse Bulletin* **134**, 1–40 (1979).
183. G. Vidal, Micropalaeontology and biostratigraphy of the Upper Proterozoic and Lower Cambrian sequence in East Finnmark, Northern Norway. *Norges Geologiske Undersøkelse* **362**, 1–53 (1981).
184. M. W. Wallace, A. v. S. Hood, A. Shuster, A. Greig, N. J. Planavsky, C. P. Reed, Oxygenation history of the Neoproterozoic to early Phanerozoic and the rise of land plants. *Earth Planet. Sci. Lett.* **466**, 12–19 (2017).
185. W. Wei, D. Wang, D. Li, H. Ling, X. Chen, G. Wei, F. Zhang, X. Zhu, B. Yan, The marine redox change and nitrogen cycle in the early Cryogenian interglacial time: Evidence from nitrogen isotopes and Mo contents of the basal Datangpo Formation, Northeastern Guizhou, South China. *J. Earth Sci.* **7**, 233–241 (2016).
186. G.-Y. Wei, W. Wei, D. Wang, T. Li, X. Yang, G. A. Shields, F. Zhang, G. Li, T. Chen, T. Yang, H.-F. Ling, Enhanced chemical weathering triggered an expansion of euxinic seawater in the aftermath of the Sturtian glaciation. *Earth Planet. Sci. Lett.* **539**, 116244 (2020).
187. C. Wu, T. Yang, G. A. Shields, X. Bian, B. Gao, H. Ye, W. Li, Termination of Cryogenian ironstone deposition by deep ocean euxinia. *Geochem. Perspect. Lett.* **15**, 1–5 (2020).
188. Q. Ye, J. Tong, S. Xiao, S. Zhu, Z. An, L. Tian, J. Hu, The survival of benthic macroscopic phototrophs on a Neoproterozoic snowball Earth. *Geology* **43**, 507–510 (2015).
189. Y. Ye, H. Wang, L. Zhai, X. Wang, C. Wu, S. Zhang, Contrasting Mo-U enrichments of the basal Datangpo Formation in South China: Implications for the Cryogenian interglacial ocean redox. *Precambrian Res.* **315**, 66–74 (2018).
190. H. Yoshioka, Y. Asahara, B. Tojo, S.-I. Kawakami, Systematic variations in C, O, and Sr isotopes and elemental concentrations in Neoproterozoic carbonates in Namibia: Implications for a glacial to interglacial transition. *Precambrian Res.* **124**, 69–85 (2003).
191. W. Yu, T. J. Algeo, Y. Du, B. Maynard, H. Guo, Q. Zhou, T. Peng, P. Wang, L. Yuan, Genesis of Cryogenian Datangpo manganese deposit: Hydrothermal influence and episodic post-glacial ventilation of Nanhua Basin, South China. *Palaeogeograph. Palaeoclimatolog. Palaeoecolog.* **459**, 321–337 (2016).

Acknowledgments: We thank I. Fairchild, T. Spencer, and L. Alcott for insightful discussion. **Funding:** This work was supported by NERC project NE/R010129/1 (to F.T.B., S.W.P., B.J.W.M., and G.A.S.), NERC SPHERES Doctoral Training Partnership NE/L002574/1 (to A.J.K.), National Natural Science Foundation of China 42063009 (to Y.F.), and Australian Research Council ARC DP170100556 ARC DP200100004 (to J.J.B.). **Author contributions:** Conceptualization: F.T.B., S.W.P., and A.J.K. Methodology: F.T.B., S.W.P., B.J.W.M., A.J.K., and G.L.H. Investigation: F.T.B., S.W.P., A.J.K., Y.S., M.A.K., K.-J.H., Y.F., B.S., X.-K.Z., J.L., L.M.v.M., J.J.B., B.J.W.M., and G.L.H. Visualization: F.T.B. Supervision: S.W.P., G.A.S., and B.J.W.M. Writing—original draft: F.T.B. Writing—review and editing: F.T.B., S.W.P., G.A.S., A.J.K., Y.S., B.J.W.M., J.J.B., M.A.K., and G.L.H. **Competing interests:** The authors declare that they have no competing interests. **Data and materials availability:** Full compilation of Cryogenian data and references is provided with corresponding age models in table S1, and radiometric ages and corresponding references are provided in table S3. Data analyzed in this study are provided in full in tables S4 and S5. All data needed to evaluate the conclusions in the paper are present in the paper and/or the Supplementary Materials.

Submitted 25 November 2022
Resubmitted 17 May 2023
Accepted 25 July 2023
Published 25 August 2023
10.1126/sciadv.adf9999
