# Supplementary material for: Biological diversification linked to environmental stabilization following the Sturtian Snowball glaciation
Source: Sci Adv. 2023 Aug 25;9(34):eadf9999. doi: 10.1126/sciadv.adf9999 (PMC10456883; doi:10.1126/sciadv.adf9999)
Supplement: Supplementary file 2 — Supplementary Text Figs. S1 to S8 Legend for table S1 Tables S2 to S5 References [file sciadv.adf9999_sm.v2.pdf]

CORRECTED 1 NOVEMBER 2023

Science Advances
AAAS

Supplementary Materials for

Biological diversification linked to environmental stabilization following the Sturtian Snowball glaciation

Fred T. Bowyer *et al.*

Corresponding author: Fred T. Bowyer, fred.bowyer@ed.ac.uk

Sci. Adv. **9**, eadf9999 (2023)
DOI: 10.1126/sciadv.adf9999

The PDF file includes:

Supplementary Text
Figs. S1 to S8
Legend for table S1
Tables S2 to S5
References

Other Supplementary Material for this manuscript includes the following:

Table S1

Figure 1F Abbreviations of Formation/Member names: Dur. – Durkan Member of the Ice Brook Formation; Delt. – Delthore Member of the Ice Brook Formation; SDL – Sourdough Limestone; MG – Mountain Girl submember; Bon. – Bonahaven Formation; Deg. – Degnish Limestone; Ard. – Ardrishaig Phyllite; Craig. – Craignish Phyllite; D. – Dongshanfeng Formation; G. – Gucheng Formation; Fiq/Shar. – Fiq/Shareef Formation; St. – Sturt; Ap. – Appila; Wily. – Wilyerpa; Br. – Brighton Limestone; Ba. – Balcanoona Formation; An. – Angepena Formation; Wt. – Weetootla dolomite; Yan. – Yankaninna Formation; Am. – Amberoona Formation; En. – Enorama shale Member; Trez. – Trezona Formation; Elat. – Elatina Formation; Why. – Whyalla Sandstone; Slang. – Slangen Member; Wilson. – Wilsonbreen Formation.

Exploring alternative age model outputs relative to age model-independent trends in FOAM CO_2-temperature and O_2 saturation of surface seawater

Uncertainty remains in the precise timing of Marinoan re-glaciation. This requires that alternative non-glacial durations are explored, and the associated timings of fossil occurrences and geochemical trends relative to age model-independent trends in climate model outputs are considered. Below, we explore three alternative non-glacial chronostratigraphic frameworks that calibrate all data and fossil occurrences within non-glacial durations of 10, 15.5 and 21 Myr, and then discuss the resulting trends relative to fixed outputs of the FOAM climate model (Fig. S1A). All age models employ the relative correlation of lithostratigraphic composite profiles shown in Fig. 1F.

Models B and B' (Non-glacial duration 10 Myr)

Model B assumes an age of ca. 660 Ma for global Sturtian deglaciation and ca. 650 Ma for Marinoan re-glaciation (Table S1). Whilst this remains consistent with the majority of available radiometric constraints, it is inconsistent with one zircon U-Pb age from an ash bed within the basal Datangpo Fm of South China (660.98 ± 0.18(0.31)[0.74] Ma; date uncertainties reported in ±X/Y/Z format, where X is the analytical uncertainty in the absence of all external errors, Y includes X and the tracer calibration uncertainty, and Z includes Y and the ^{238}U decay constant uncertainty; *17*), even when considering maximum uncertainty associated with incorporation of analytical, tracer calibration and U-Pb decay constant uncertainties. Model B assumes that the radiometric age of the Thorndike submember (South Park Member, Kingstone Peak Fm) approximates the depositional age near the end of the Keele Peak (Cn4), consistent with Model A. Therefore, model B may calibrate all data near the minimum possible duration of the non-glacial interval, notwithstanding the possibility for a Trezona anomaly (Cn5) duration of <1 Myr. Given the likelihood that Sturtian deglaciation occurred closer to ca. 661 Ma, a 10 Myr duration would calibrate the onset of Marinoan re-glaciation during the rising limb of Cn5.

Model B serves to shorten the timing of recovery from the initial post-Sturtian anoxic episode, but does not result in any notably different pattern or timescale of geochemical response relative to that shown between the geochemical data calibration of Model A and the FOAM climate model output. Given the more reasonable deglaciation age of ca. 661 Ma and negligible difference in geochemical calibration between models A and B, we consider all alternative models relative to Model A (Fig. S1B).

Model C (Non-glacial duration ca. 15.5 Myr)

Model C assumes an age of ca. 661 Ma for Sturtian deglaciation, but extends the duration of the non-glacial interval to 15.5 Myr, with Marinoan re-glaciation occurring at ca. 645.5 Ma (Fig. S1C). Model C results in crystallization of the dated Thorndike submember zircons approximately coincident with the Taishir anomaly (Cn3), rather than the Keele Peak as suggested by (*13*). This remains possible when considering that the Thorndike age is conservatively interpreted to represent a maximum depositional age (M.D.A.; *19*), and in recognition of the complexity of preserved $\delta^{13}C_{carb}$ within the regional depositional environment of the Thorndike submember (*13*). Given these associated uncertainties, we consider models A and C to be equally plausible based on integrated consideration of all available radiometric data.

Whilst the duration of the non-glacial interval is 3.5 Myr longer in Model C than Model A, the calibration of all geochemical and biotic data remain internally consistent. The result is that the timing of stabilization of geochemical trends (e.g., equilibration of Fe-speciation data) in inner shelf to slope environments appear to align perfectly with the timing of modelled climate-carbon steady state ca. 4.5–5 Myr post-Sturtian deglaciation (Fig. S1A, S1C). In Model C, this timing also aligns with the maximum possible age for the first appearance of green algal and putative sponge steranes (Fig. S1C).

Model D (Non-glacial duration ca. 21 Myr)

Model D also accepts an age of ca. 661 Ma for Sturtian deglaciation, but extends the non-glacial interval to occupy 21 Myr (Fig. S1D), which is close to the maximum possible duration based a zircon U-Pb age of 639.29 ± 0.26 Ma from an ash interbedded with the Marinoan-age Ghaub diamictite (*21*). This age model strongly suggests that the dated horizon in the Thorndike submember is younger than the maximum depositional age from zircon U-Pb geochronology (Fig. S1D). This age model also extends the minimum age of the basal black shale interval of the Datangpo Fm to ca.

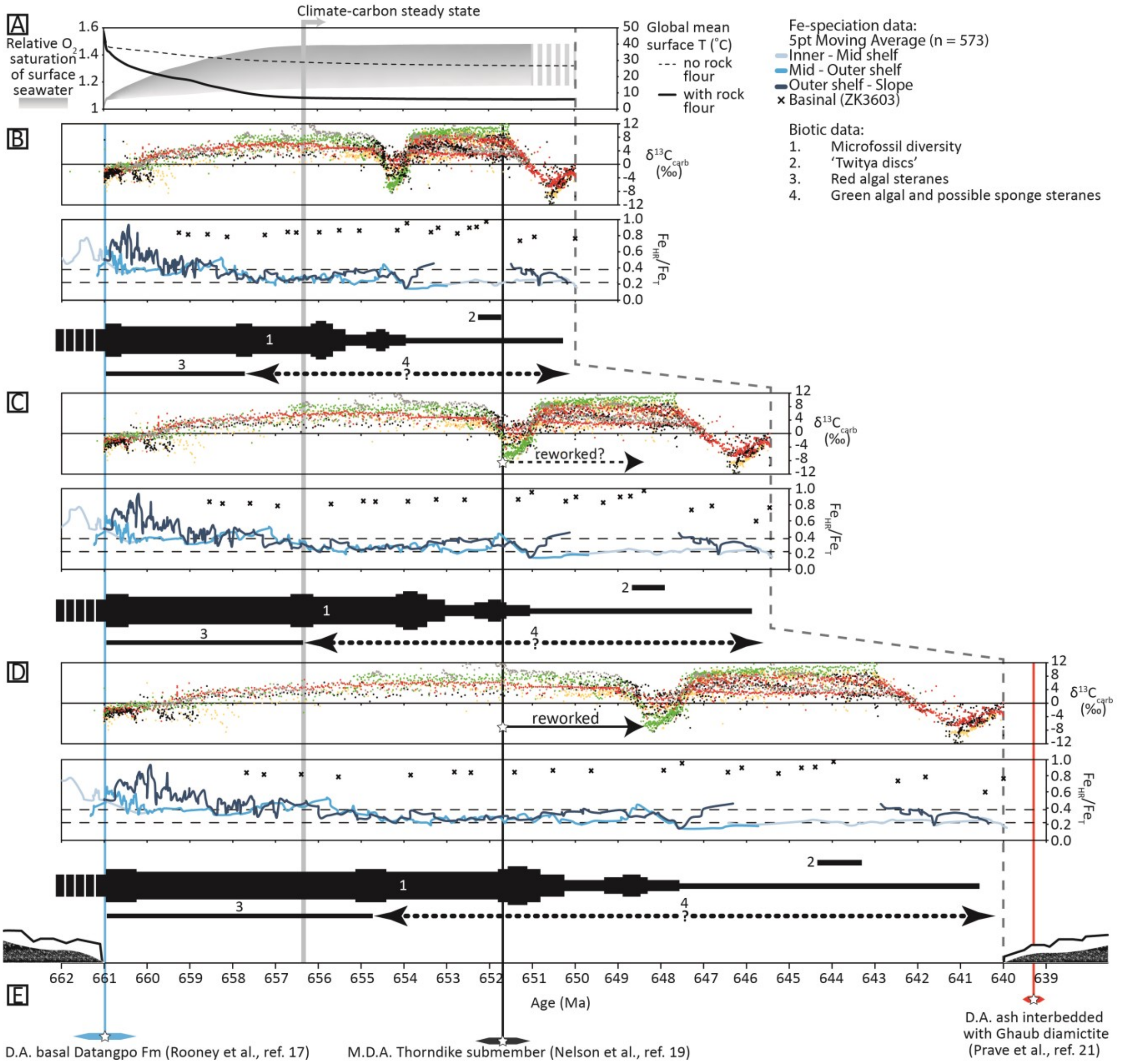

Fig. S1. Age model-independent trends in FOAM model outputs relative to alternative age model outputs (Table S1). (A) Global mean surface temperature and calculated relative O_2 saturation of surface seawater (grey envelope) based on FOAM CO_2-temperature relationship. Vertical grey line represents approximate timing of climate-carbon steady state. **(B) to (D)** $\delta^{13}C_{carb}$, Fe_{HR}/Fe_T and summarized fossil occurrences resulting from age models A (B), C (C), and D (D) (Table S1). **(E)** Key radiometric ages considered in construction of each age model (*17*, *19*, *21*). D.A. – Depositional age, M.D.A. – Maximum depositional age. Horizontal arrows that radiate from the vertical black line highlight the degree of possible reworking of the dated horizon in the Thorndike submember required to satisfy each $\delta^{13}C_{carb}$ age model (see text for details).

656 Ma, which is younger than radiometric constraints from the overlying siltstone interval in other sections (*17*).

In light of the caveats, we currently consider this maximum non-glacial duration less likely than models A-C. However, even in Model D, the interval of geochemical re-equilibration following Sturtian deglaciation remains largely within the 4.5–5 Myr interval of decreasing global temperature and increasing O_2 saturation state of surface seawater, and preceding the attainment of climate-carbon steady state (Fig. S1D).

Cryogenian age model limitations

Given ongoing uncertainties in paleogeographic reconstructions, and variability in regional and global $\delta^{13}C_{carb}$ associated with possible local effects and variable diagenetic regimes, Sturtian deglaciation and Marinoan reglaciation are conservatively considered to have been globally synchronous at the resolution of each age model. Globally synchronous Sturtian deglaciation is consistent with current radiometric constraints and associated uncertainties (Fig. 1G) (*16–18*), and is also consistent with a geologically instantaneous global deglaciation predicted from climate model simulations under immediately post-glacial super-greenhouse conditions (*78*).

With the exception of the timing of Sturtian deglaciation and the associated Rasthof anomaly, and the age of positive $\delta^{13}C_{carb}$ values associated with the Keele Peak (but see above), the non-glacial chemostratigraphic age model presented herein suffers from a dearth of radiometric ages to anchor the durations and absolute ages of individual $\delta^{13}C_{carb}$ excursions (Fig. 1B, G). As such, only relative trends in $\delta^{13}C_{carb}$ can presently be used for correlation. As noted in the main text, between-section variability in the magnitude of $\delta^{13}C_{carb}$ during each $\delta^{13}C_{carb}$ excursion of the Cryogenian non-glacial is well established, especially across the Taishir (Cn3) and Keele Peak (Cn4) anomalies. This variability in magnitude may reflect differences in the fidelity of preservation of seawater $\delta^{13}C$ associated with fluid-buffered versus sediment-buffered diagenetic regimes (*12*, *13*, *23*), which leads to some ambiguity in the correlation of minor $\delta^{13}C_{carb}$ trends that may be present within the Cn2 and Cn4 intervals, and the discrimination between the Cn3 versus Cn5 excursions. The latter case is especially problematic when considering successions of mixed lithology that do not host both excursions; perhaps most clearly demonstrated by the negative $\delta^{13}C_{carb}$ anomaly recorded in the lower Ice Brook Formation in some sections of the Wernecke Mountains (Yukon) (*10*). Specifically, the excursion in the lower Ice Brook Formation at Goz A section may correlate with either the Taishir or Trezona anomaly, leading to ambiguity in the correlation of geochemical data from the

overlying siliciclastics of the upper Ice Brook Formation, either to an interval equivalent to the Keele Peak or post-dating the Trezona anomaly. We follow the latter scenario, which is favoured by (*10*) and (*23*), and may imply a more continuous (post-Trezona) non-glacial record in Laurentia (consistent with a low-latitude position and/or limited removal of pre-glacial strata) relative to other globally-distributed successions. Importantly, these uncertainties do not affect the trends recorded by the geochemical proxy data compiled in this study, which are all bracketed by successive $\delta^{13}C$ excursions.

Lastly, we also recognize that the majority of depositional environments were likely semi-restricted, particularly before the onset of Sturtian deglaciation and nearing the onset of Marinoan reglaciation (e.g., *13*, *40*). Whilst consistent trends in $\delta^{13}C_{carb}$ in most regions may attest to at least partial connectivity with a well-mixed global ocean, we do not use proxies of more regional significance (e.g., $\delta^{34}S_{py}$) to inform inter-regional correlation. This reduces the probability of circular reasoning when interpreting global trends from regional geochemical datasets. Despite this approach, there are some aspects of our age model associated with assumed stratigraphic continuity that remain difficult to verify. These uncertainties are specifically associated with the onset ages and durations of deposition in continuous siliciclastic successions that do not have associated radiometric constraints, including the Arena Formation, Greenland, and the Xiangmeng Formation of South China (discussed further in *14*, *42*). In our age model, these successions are considered to be continuous throughout the full duration of the non-glacial interval in some sections. However, we note that, even if these successions were deposited over a shorter duration (e.g., Cn1 to Cn3 interval only), this would not affect the overall trends recorded by the proxy data compiled in this study, and would also maintain the same sequence of geochemical trends relative to fossil occurrences.

Geological background

The Amadeus Basin and Adelaide Superbasin, Australia

The complex subdivision and tectonic histories of the Centralian and Adelaide superbasins have been described by (*79*) and (*80*). Paleogeographic reconstructions suggest a gradual equatorial migration of Australian cratonic blocks in the northern hemisphere from ~45–30° during the Sturtian cryochron, to ~18° by the end of the end of the Marinoan cryochron, coincident with the Cryogenian-Ediacaran boundary (*77*).

In the central-western Amadeus Basin, the Bloods Range NTGS Drillhole BR05-DD01 comprises Sturtian glacial diamictites of the Areyonga Formation, overlain by 330.8 m of mid-outer ramp dolomitic shale and siltstone of the Aralka Formation (core depth 484.60–153.80 m). Rare

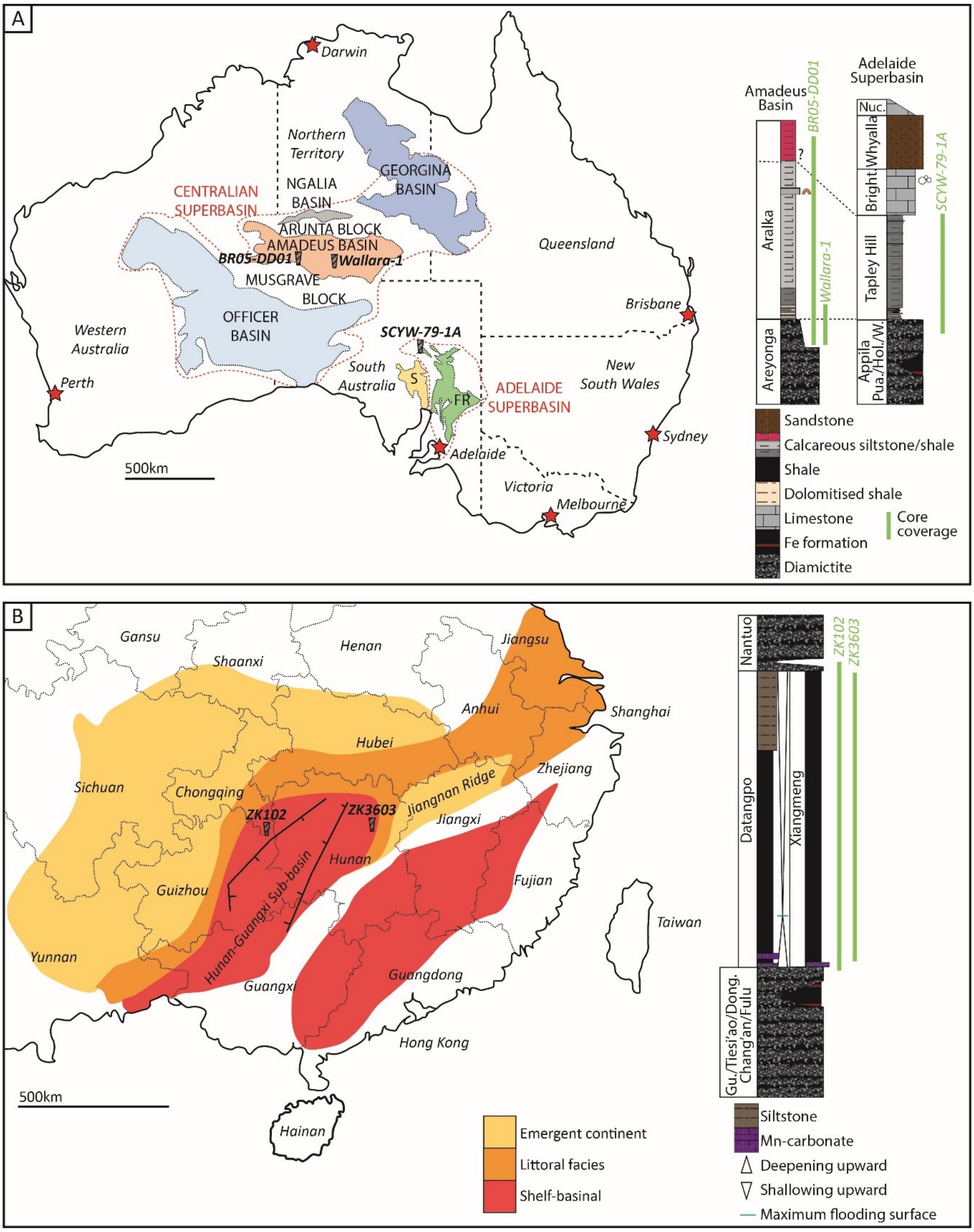

Fig. S2. Positions of analyzed drill cores. (A) BR05-DD01 and Wallara-1 cores of the Amadeus Basin (Centralian Superbasin), and SCYW-79-1A core of the Adelaide Superbasin, Australia (modified after ref. *79*). The sampled interval is shown by relative core length on schematic lithostratigraphic columns for each succession. S = Stuart Shelf, FR = Flinders Ranges, Pua. = Pualco tillite, Hol. = Holowilena ironstone, W. = Wilyerpa Formation, Bright. = Brighton limestone, Nuc. = Nuccaleena Formation. **(B)** ZK102 and ZK3603 cores of the Hunan-Guangxi Sub-basin of the Nanhua Basin, South China, with simplified regional paleodepth (modified after ref. *34*). The sampled interval is represented by relative core length on schematic lithostratigraphic columns. Gu. = Gucheng Formation, Dong. = Dongshanfeng Formation.

stromatolitic carbonate interbeds (core depth 191–154 m) in the upper part of the Aralka Formation include the stromatolites *Atilanya fennensis* (191.3–186.5 m) and *Tungussia inna* (156–154 m; *81*). At 153.80 m, the succession switches to immature, red siliciclastics that have been suggested to correlate with the Ediacaran to Cambrian-age Arumbera sandstone (*81*, *82*), which would imply a major hiatus. However, this correlation remains highly uncertain, and these strata may equally represent continued shallowing during the non-glacial interval, and stratigraphic correlation with the Marinoan-age Whyalla sandstone of the Adelaide Superbasin (Fig. S2A). The stromatolite *Atilanya fennensis* (present at 191.3–186.5 m) may also be present in the Balcanoona Formation of the Umberatana Group of the Adelaide Superbasin (*81*). This potential interbasinal correlation may further support correlation of the interval 484.6–153.8 m in BR05-DD01 with the Cn1-Cn3 interval only (contra Model B', see below) based on chemostratigraphy of the Balcanoona Formation (*7*). Overall, the Aralka Formation siliciclastics in core BR05-DD01 may largely correlate to the dominantly siliciclastic Ringwood Member of the Aralka Formation further east in the Amadeus Basin (models A-D; *9*), or may represent continuous siliciclastic deposition that was laterally equivalent to both the Ringwood and overlying Limbla members (Model B'). In the latter case, the youngest samples of core BR05-DD01 may correlate with the Cn4 interval, and the overall trends in Fe and P speciation data would remain consistent between regions but high $\delta^{34}S_{py}$ data in the younger half of BR05-DD01 (31.60–51.0‰) may imply the formation of superheavy pyrite in the Amadeus Basin based on corresponding $\delta^{34}S_{CAS}$ data from the Adelaide Superbasin (25.10–32.30‰) (*63*). Importantly, if Model B' is correct, the youngest biomarker data from BR05-DD01 (sample 11J005 i of ref. *2*), recorded at 242.2 m, post-date carbon-climate steady state, approximately coincident with the Taishir anomaly (Cn3). Sediments of the Wallara-1 core were deposited in the Amadeus Basin to the east of BR05-DD01, and constitute a similar but condensed lithological profile comprising diamictite of the Areyonga Formation overlain by dolomitic shale of the lowermost Aralka Formation.

Core SCYW-79-1A recovers strata of the northwest Adelaide Superbasin, along the northeastern Stuart Shelf at the margin with the Torrens Hinge Zone (*80*). Cryogenian strata of the Adelaide Superbasin begin with Sturtian glacial deposits of the lower Umberatana Group (Yudnamutana Subgroup), including the Appila and Sturt tillites, and equivalent Pualco and Wilyerpa diamictites and interbedded ironstone deposits to the east of SCYW-79-1A (*32*, *33*). Carbonaceous shales and siltstones of the Tapley Hill Formation (Nepouie Subgroup) conformably overlie the Appila tillite in SCYW-79-1A, and are laterally equivalent to the Aralka Formation of the Amadeus basin, and correlative transgressive siliciclastic deposits throughout the Centralian Superbasin (Fig. S2A; *33*). Maximum flooding in the Adelaide Superbasin is recorded by the lower Tapley Hill Formation

(Tindelpina Shale Member), which gradually shallows-upwards and is conformably overlain by the shallow marine oolitic grainstone of the Brighton Limestone (*33*). To the east, shale of the upper Tapley Hill Formation interfingers with equivalent marine carbonates of the Balcanoona Formation, which in turn shallow to iron-rich peritidal dolomite and dolomitic shale of the Angepena Formation (*7*, *83*, *84*). In SCYW-79-1A, the Brighton Limestone is overlain by the Whyalla sandstone, which has been interpreted as a periglacial aeolian sandstone that was deposited on the Stuart Shelf equivalent to the Elatina Formation diamictite of the Marinoan cryochron to the east (*70*).

The Aralka and Tapley Hill formations contain a characteristically depauperate Cryogenian non-glacial microfossil assemblage dominated by leiosphaerid acritarchs, alongside prokaryotic *Synsphaeridium*, simple filamentous forms, and rare occurrences of ornamented and double vesicle acritarchs (*85*). The associated assemblage is similar to microfossil assemblages from the correlative Macdonaldryggen Member of the Elbobreen Formation, Svalbard, and the lower Datangpo Formation, South China (Fig. 1E, F; *85*, *86*).

The Nanhua Basin, South China

The Nanhua Basin developed along the southeast margin of the Yangtze Block, South China, as a consequence of rifting during the Tonian break-up of Rodinia (*87*). Paleogeographic reconstructions suggest a gradual equatorial transit of the Yangtze Block in the northern hemisphere during the Cryogenian Period, from ~50–60° during the Sturtian cryochron to ~30–40° during the Marinoan cryochron (*77*, *88*, *89*). Cryogenian strata of the Nanhua basin were largely deposited within the Hunan-Guangxi sub-basin in eastern Guizhou Province and western Hunan Province (Fig. S2B), and include glacial strata of Sturtian age and the Marinoan-equivalent Nantuo Formation separated by the non-glacial Datangpo and Xiangmeng formations (*87*). Sturtian-age diamictite-bearing strata include the Chang'an, Xieshuihe, Liangjiehe, Dongshanfeng, Gucheng and Tiesi'ao formations (*34*, *90–92*). Iron-rich sandstones and banded iron formation deposits of the Fulu Formation are variably reported as overlying the Gucheng Formation (*93*), comprising the Liangjiehe and Gucheng members (*90*), or underlying the Gucheng Formation diamictites (*92*). However, litho- and sequence stratigraphic constraints support a syn-Sturtian position for the Fulu Formation, equivalent to the Xieshuihe and Liangjiehe formations, and conformably underlying the Gucheng Formation (*34*, *91*, *92*).

A Sturtian age for the lower diamictite-bearing deposits of the Nanhua Basin is confirmed by a zircon U-Pb SHRIMP age of 691.9 ± 8.0 Ma from tuffaceous siltstone of the Xieshuihe Formation, and a zircon U-Pb CA-ID-TIMS age of 658.8 ± 1.49 Ma from a tuff layer at the top of the Tiesi'ao Formation (*91*, *94*). More recent zircon U-Pb CA-ID-TIMS ages of 660.98 ± 0.74 Ma and 657.17 ±

0.78 Ma have been reported from tuff deposits interbedded with the upper Tiesi'ao and lower Datangpo formations, respectively (*17*). These ages, alongside a Re-Os isotopic model age (660.6 ± 3.9 Ma) of the upper Tiesi'ao Formation (*17*), confirm equivalence of the Datangpo and Xiangmeng formations with globally distributed non-glacial deposits of the Twitya Formation, northwest Canada (Re-Os: 662.4 ± 3.9 Ma; *16*), lower Taishir cap carbonate, Mongolia (Re-Os: 659 ± 4.5 Ma; *95*), and the Umberatana Group, Australia (zircon U-Pb CA-ID-TIMS: 663.03±0.76 Ma; *18*). Lastly, a zircon U-Pb LA-ICP-MS age of 651.2 ± 3.3 Ma has recently been reported from an ash interbed within the upper unit of the Datangpo Fm, 0.4 m below the unconformable contact with overlying Marinoan-age diamictite (*20*).

Here we present data from two drill core profiles that transcend continuous siliciclastic deposits of the Datangpo and Xiangmeng formations (Fig. S2B). Core ZK102 was recovered in the Daotuo area near Tongren City of eastern Guizhou Province and records outer shelf–slope deposits of the Datangpo Fm. Core ZK3603 was recovered in eastern Hunan Province and records deeper, slope–basin deposits of the Xiangmeng Fm (Fig. S2B). In both cores, the contacts between the Datangpo/Xiangmeng formations and the underlying and overlying diamictites appear to be conformable (*14*).

The lithostratigraphy of drill core ZK102 closely resembles that described for neighbouring drill core ZK105 (Daotuo section of *11*), and comprises a basal transgressive unit of ~10 m interbedded Mn-rich carbonate and black shale that conformably overlies diamictite of the Tiesi'ao Formation, followed by ~30 m organic-rich black shale, ~160 m grey shale, and ~60 m grey siltstone. The boundary between the upper Datangpo Formation and overlying lower Nantuo Formation in the Daotuo area is locally conformable in drill core and outcrop sections (*96*). The lithostratigraphy of core ZK3603 has been subdivided into 4 units; 0.4 m interbedded Mn-carbonate and organic-rich black shale, 82 m black shale, 1.1 m grey-black Mn-bearing limestone, and 5.5 m calcareous and pyritic grey-black shale (*14*). The microfossils assemblage of the lower Datangpo Formation is dominated by cyanobacteria and a variety of small vesicle acritarchs including (but not limited to) *Protosphaeridium*, *Synsphaeridium*, *Trachysphaeridium* and *Leiosphaeridia* (*86*, *97*).

Detailed methods

Drill core material was trimmed of any visible surficial alteration or veining and samples with macroscopic euhedral pyrite were not used. Samples were crushed and powdered using a tungsten carbide piston and agate disc mill. Unless specified otherwise, chemical extractions and analyses were undertaken at the Cohen Laboratories, School of Earth and Environment, University of Leeds.

Elemental analysis

Bulk digestions were performed on 50-80 mg of sample powder using HNO_3-HF-$HClO_4$ at ~70°C for 12 h, followed by addition of H_3BO_3 to prevent the formation of Al complexes. Major element (Al, Ca, Fe, K, Mg, Mn, Na, P) concentrations were measured using inductively coupled plasma optical emission spectrometry (ICP-OES, Thermo Fisher iCAP 7400), and trace element (Mo, Re, U) concentrations were measured using inductively coupled plasma mass spectrometry (ICP-MS, Thermo Fisher iCAPQc). Total digestions of a standard material (SBC-1, United States Geological Survey) yielded values within the certified range for all elements analyzed (<3.4%).

TOC and organic C isotopes

Samples were fully decarbonated via 3-4 12 h HCl (10% vol/vol) dissolutions, repeatedly washed with 18MΩ H_2O to remove all residual acid (pH >4), centrifuged and dried prior to analysis. Carbon concentrations were measured using a LECO carbon-sulfur analyzer, with replicate analyses yielding a precision of ±0.09 wt% (2σ). The organic carbon fractions of samples from core ZK102 were analyzed for organic C isotopes ($\delta^{13}C_{org}$) by elemental analyzer isotope ratio mass spectrometry (EA-IRMS) at Iso-Analytical Ltd. All data are reported relative to the Vienna Pee Dee Belemnite standard, with replicate analyses yielding a precision of ±0.14‰ (1σ). Organic carbon isotopic ratios in samples from the BR05-DD01 core were measured by EA-IRMS in IsoLab at the University of Washington. Replicate analyses yielded a precision of ±0.03% (1σ) for TOC and ±0.13‰ (1σ) for $\delta^{13}C_{org}$.

Fe speciation

Fe speciation was performed after the established methodology of Poulton and Canfield (*71*) to extract operationally-defined Fe phases including Fe associated with carbonates (Fe_{carb}), ferric oxides (Fe_{ox}), magnetite (Fe_{mag}) and pyrite (Fe_{py}). The sum of these Fe pools constitutes the proportion of Fe that is considered highly reactive (Fe_{HR}) towards dissolved sulfide. Fe_{carb}, Fe_{ox} and Fe_{mag} were extracted sequentially using buffered sodium acetate (pH 4.5, 50°C, 48 h), sodium dithionite (ambient temperature, 2 h), and ammonium oxalate (ambient temperature, 6 h). Extract solutions were measured for their Fe concentrations by atomic absorption spectrometry (AAS). Fe_{py} was trapped as Ag_2S after sample reaction with boiling chromous chloride [Cr(II)Cl_2] under anoxic conditions (*98*). A pre-leach in boiling 50% (vol/vol) HCl confirmed that no acid volatile sulfides were present.

All analyzed samples have total Fe concentrations (Fe_T) >0.5 wt%, including 5 carbonate-rich samples from the base of the Datangpo Formation in core ZK102. In order to ensure that the sequential leach quantitatively extracted each operationally-defined Fe phase, a recently-certified Fe speciation reference material (WHIT) was run alongside each batch (*99*). The results are in agreement with certified values, with mean values of Fe_{carb} = 0.599 wt%, Fe_{ox} = 0.069 wt%, and Fe_{mag} = 0.107 wt%, and relative standard deviations (RSDs) of <5% (n=17).

Pyrite sulfur isotopes

The isotopic composition of pyrite sulfur ($\delta^{34}S_{py}$) was determined on Ag_2S produced through sample reaction with boiling chromous chloride. Samples from core ZK102 were analyzed for $\delta^{34}S_{py}$ by EA-IRMS at Iso-Analytical Ltd. All data are reported relative to the Vienna Canyon Diablo Troilite standard. Replicate analyses yield a precision of ±0.17‰ (1σ). Samples from cores BR05-DD01, SCYW-79-1A and ZK3603 were analyzed at the Cohen Laboratories, School of Earth and Environment, University of Leeds and replicate analyses yield a precision of ±0.24‰ (1σ).

P phase association

Selected samples were subjected to a separate sequential P extraction following the modified SEDEX methodology (*75*) adjusted for use on ancient sediments (Table S5; *76*). This method isolates operationally-defined P pools including P bound in Fe(oxyhydr)oxide minerals (P_{Fe}), organic matter (P_{org}), authigenic carbonate fluorapatite, biogenic apatite and $CaCO_3$ (P_{auth}), and detrital apatite (P_{det}). Reactive P (P_{reac}) equates to the sum of P_{Fe}, P_{auth} and P_{org} (*36*). The concentrations of P in P_{org}, P_{auth} and P_{det} leachates were measured spectrophotometrically using the molybdate-blue method on a Spectonic GENESYS 6 at a wavelength of 880 nm, whereas P concentrations in the P_{Fe} leachates (including P_{Fe1}, P_{Fe2} and P_{mag}) were measured by ICP-OES (*76*). A mean P recovery of 89% of P_{Tot} (as measured by ICP-OES after total digestion) was achieved by the sequential extraction protocol. Replicate analyses of the Fe speciation standard (WHIT, n=9) gave a relative standard deviation of <7% for all extraction steps, with the exception of P_{Fe} (18%) due to very low concentrations of P_{Fe} (near detection).

Detailed paleoredox and P assessment

The importance of a multi-proxy dataset in the characterization of regional paleoredox

We note recent challenges to the Fe speciation technique, and interpretations of paleoredox data based on Fe speciation alone (*100*). Indeed, there are several caveats that must be considered

carefully when evaluating Fe speciation data, which are well described in the literature but were ignored in the analysis of Pasquier et al. (*100*).

Firstly, sediments with low Fe concentrations (<0.5 wt%) may yield spuriously elevated Fe_{HR}/Fe_T that are not representative of depositional paleoredox conditions (*101*). Samples analyzed for this study contain appreciable Fe_T, in the range 1.46–7.77 wt% (mean = 4.22 wt%, Table S4), and all samples included in the compilation of published Fe speciation data (Figs 5, S1) contain Fe_T >0.5 wt% (Table S1).

Secondly, rapid deposition (e.g., turbidites) may mask anoxic Fe_{HR} enrichment and lead to muted Fe_{HR}/Fe_T (*102*). However, no turbidites were analyzed in this study, and up-core changes in depositional rate are evaluated and discussed relative to changes in all available paleoredox data. In particular, whilst each of our cores show sedimentological evidence for shallowing (and likely corresponding increases in depositional rate), the redox shifts do not always correspond directly with recorded lithological shifts. For example, the redox shift in BR05-DD01 occurs within a monotonous dark grey siltstone interval (Fig. 2), and dominantly occurs across a transition from black to grey shale in ZK102 (Fig. 4). In the latter case, this color change does not correspond with a decrease in sediment grain size, but more likely represents enhanced organic carbon content of the basal unit of black shale (Fig. 4A). Diagenetic models also suggest that muted Mo_{auth} would be expected in intervals of enhanced sedimentation rate (*41*). However, our deepest (and likely slowest deposited) core (ZK3603), exhibits low Mo_{auth} throughout the non-glacial interval despite occasionally elevated Fe_{py}/Fe_{HR} up to 0.77 (e.g., Fig. 4N). If increasing sedimentation rates throughout the non-glacial were solely responsible for the observed decrease in (e.g.) Mo_{auth} in shelf-slope environments, then we may expect elevated Mo_{auth} to persist throughout deeper environments where sedimentation rates remained low and pore waters were occasionally sulfidic (e.g., ZK3603).

Thirdly, the interpretation of paleoredox conditions from Fe speciation systematics can potentially be affected by the degree of chemical weathering (*103*). In some modern subtropical environments, high riverine concentrations of Fe_{HR} supplied by intense chemical weathering can bypass the inner shore zone of Fe (oxyhydr)oxide trapping (e.g., *104*), and lead to Fe_{HR}/Fe_T values in marine sediments that are enriched relative to the normal oxic threshold of 0.38 (*103*). It is thought that the initiation of Sturtian deglaciation was a consequence of super-greenhouse conditions that followed a multi-million-year build-up of atmospheric CO_2 during the Sturtian cryochron (*1*, *105*, *106*). Previous studies utilizing the chemical index of alteration (CIA) proxy have suggested a high degree of chemical weathering in the Cryogenian non-glacial Nanhua Basin (*107*, *108*). However, the pronounced and unidirectional global trends in TOC, Fe_{HR}/Fe_T, Fe_{py}/Fe_{HR}, and redox sensitive trace metal enrichments (from high to low values) are distinct from the persistently elevated CIA values

observed throughout the non-glacial Nanhua succession, and so were more likely driven by changes to depositional redox conditions (*107*). This interpretation is further reinforced by a corresponding up-core increase in diameter and decrease in abundance of framboidal pyrite morphology (e.g., *40*, *109*), which are controlled by depositional redox conditions but are independent of chemical weathering-derived Fe_{HR}.

Fe speciation has been extensively calibrated in both modern depositional environments and using ancient rocks (*39*, *73*, *101*, *103*, *104*, *110*, *111*). The ancient rocks used in calibration have by definition undergone the diagenetic transformations that Pasquier et al. (*100*) claim compromise the utility of the proxy for paleoredox interpretation. As emphasized in the literature, Fe speciation data are most appropriately used in combination with other indicators of water column paleoredox (e.g., redox sensitive trace element concentrations) and all pertinent information to constrain changes in the depositional rate and depositional environment (e.g., *112*, *113*). This is the approach taken here, which provides a particularly robust reconstruction of paleoredox conditions based on an integrated consideration of independent multiproxy redox data. Below, we assess all available proxy data to build a conservative and integrative framework for the characterization of depositional redox conditions in each studied core.

Regional variability in the marine inventory of redox sensitive trace elements

Extensive study of elemental concentrations (including redox sensitive and detrital unreactive elements) in soils from across the continental United States found notable heterogeneity in trace element concentrations and ratios traditionally used to infer enrichments relative to detrital contribution. As such, the determination of trace element enrichments relative to crustal cutoff values has been considered to oversimplify intra- and inter-regional variability (*114*). Specifically, detrital fluxes of redox sensitive trace elements may regionally fall well below the crustal average, thereby effectively concealing paleoredox-related authigenic enrichments (see below) (*114*). As such, we consider authigenic trace element concentrations (auth) where possible, but also consider trace element enrichment factors (EF) in cases where the calculation of authigenic concentrations obscure trends in the dataset (e.g., U, see below). When discussing trace element enrichment factors, all values and trends are considered relative to local oxic baseline values for each dataset (see below).

Enrichment factors for redox sensitive trace elements represent enrichment ($X_{EF} > 1$) or depletion ($X_{EF} < 1$) relative to average upper continental crust (UCC) composition (UCC compositions after ref. *115*), calculated as:

$X_{EF} = [(X/Al)_{sample}/(X/Al)_{UCC}]$

In many previous studies, enrichment factors (X_{EF}) have been calculated relative to their concentrations in post-Archean average shale (PAAS). However, many ancient marine shales were likely deposited under anoxic conditions that would themselves result in redox sensitive trace element enrichments, leading to slightly elevated average PAAS relative to oxic shale composition (e.g., see discussion in ref. *114*).

Figure S3 shows Mo versus U enrichments for all data, and separated for 'possibly euxinic' data according to threshold Fe speciation ratios (Fe_{HR}/Fe_T >0.38, Fe_{py}/Fe_{HR} >0.60). This figure clearly demonstrates that detrital background U and Mo supplies to the Australian and South China successions were distinct, with unusually depleted oxic baseline Mo and U compositions in South China relative to UCC composition. It is also clear that the modern open marine environments used to calibrate specific drawdown mechanisms for Mo and U enrichment are not appropriate to define post-Sturtian depositional redox conditions. Specifically, during and in the immediate aftermath of Sturtian deglaciation, seawater Mo and U concentrations are highly likely to have been lower than present due to the preceding long-term (60 Myr) trace metal drawdown under dominantly anoxic Snowball ocean conditions (*42*). With an oceanic trace metal inventory essentially exhausted, the burial of Mo and U under anoxic and euxinic/sulfidic post-Sturtian conditions was limited by the supply of these elements from the continental weathering influx. The average elemental composition of source areas that supplied detrital material to individual depositional environments following Sturtian deglaciation is likely to have been highly variable (e.g., *114*). Furthermore, sulfidic conditions registered by our multiproxy paleoredox scheme were geographically isolated in the immediate aftermath of the Sturtian glaciation (Figs. S4, S5). Regional variability in the degree of trace metal enrichment is therefore expected and this is clearly demonstrated by the discrete fields occupied by the South China and Australian datasets in Mo_{EF}/U_{EF} space (Figs. S3A, B). In both regions, Mo_{EF} are positively correlated with Fe_{py}/Fe_{HR} (Figs S3C, D), which is most consistent with Mo drawdown controlled by sulfide availability, rather than the operation of an Fe (oxyhydr)oxide particulate shuttle.

Authigenic trace element concentrations (X_{auth}) were calculated (*116–118*) as:

$X_{auth} = X_{sample} – [(X/Al)_{UCC} *Al_{sample}]$

These authigenic concentrations also therefore represent enrichments relative to average upper continental crust (UCC) composition (*115*). As noted above, the clear regional distinction between oxic baseline values for U and Mo (Fig. S3) demonstrate that the background concentrations of U and Mo from the detrital sources were lower in South China than Australia. When calculating authigenic concentrations, negative X_{auth} values are set to zero (*118*), thereby obscuring data trends that may be meaningful. Given that the majority of U_{auth} values in our South China dataset were reset

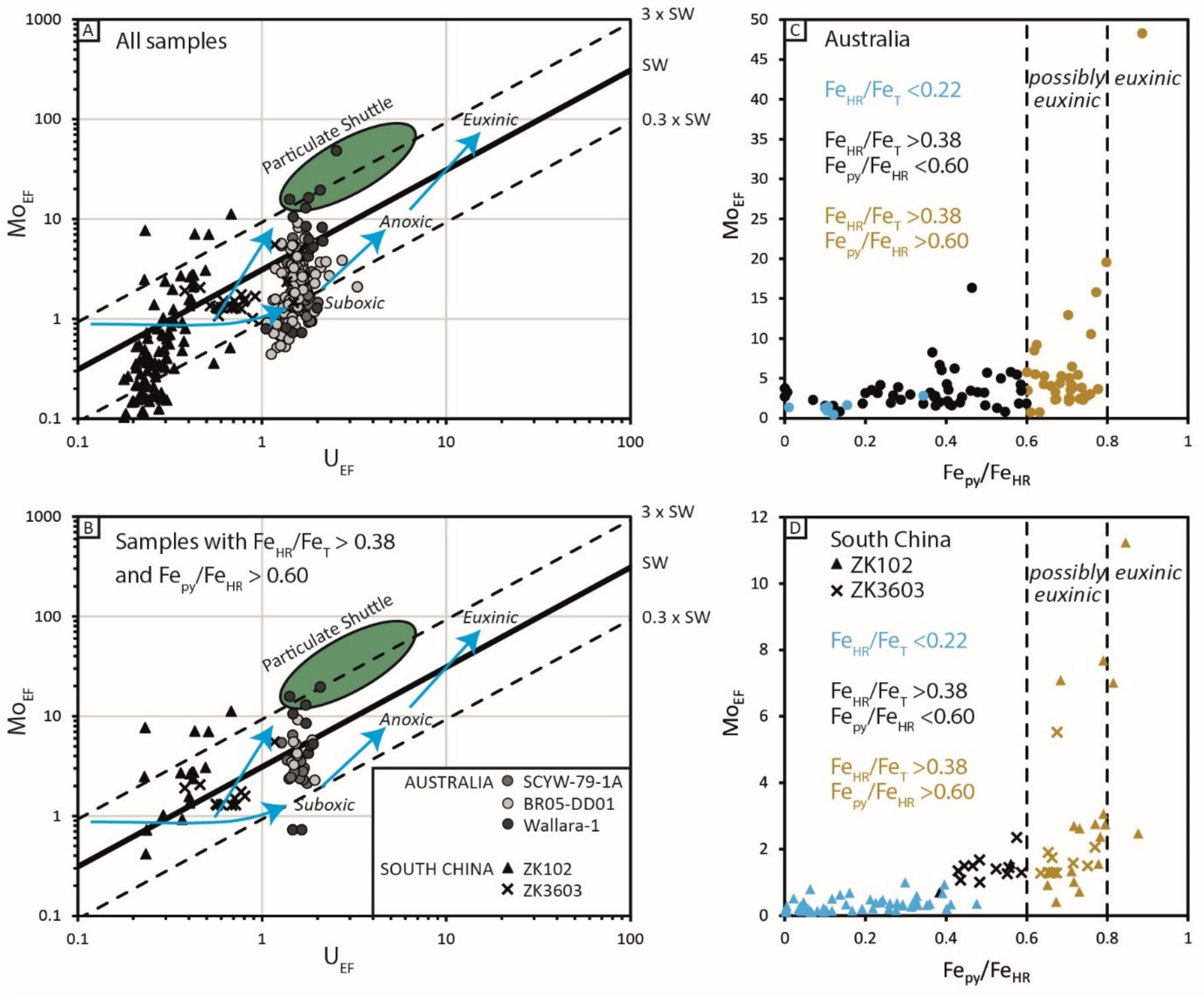

Fig. S3. Mo_{EF} vs. U_{EF} (A, B) and Mo_{EF} vs. Fe_{py}/Fe_{HR} (C, D) for analyzed Cryogenian shales. Diagrams modified after (*117*) and (*125*), with redox labels and interpretations corresponding to modern open marine basins as defined by (*125*). Solid line and dashed lines correspond to the modern seawater (SW) molar Mo/U ratio of 3.1 and multiples thereof (*125*). Arrows from 'suboxic' to 'euxinic' demonstrate the enrichment pathway expected for modern unrestricted marine environments. The arrow pointing to the green ellipse demonstrates the way in which sedimentary Mo_{EF} and U_{EF} would respond under the influence of an Fe (oxyhydr)oxide particulate shuttle. Note that Mo_{EF} scales with the degree of sulfidization of the Fe_{HR} pool (C, D), which suggests that the primary control on Mo drawdown was sulfide availability, rather than an Fe (oxyhydr)oxide particulate shuttle.

to zero, we consider both X_{auth} concentrations and trends in X_{EF} for each element throughout the non-glacial interval. As emphasized above, anoxic elemental enrichments are considered relative to oxic baseline values for each dataset in the main text and following discussion. We further note that none of the analyzed cores show any systematic trend in Al concentrations throughout the non-glacial interval that may affect trends observed in normalized trace metal enrichments.

Figures S4 and S5 show all core intervals colored by their interpreted depositional redox conditions based on a combination of Fe speciation data and observed degrees of enrichment in the redox sensitive elements Mo, U and Re. These three redox sensitive elements have differing reduction potentials and/or differing mechanisms for sedimentary enrichment, which, when considered in combination, permit a more detailed assessment of depositional redox conditions (e.g., *119*).

The removal rates of Mo, U, and Re to anoxic sediments are controlled by water column and pore water chemistry (*116*, *120*). Authigenic enrichments in U and Re occur in sediments deposited under anoxic water column conditions, in addition to more minor enrichments in anoxic sediment pore waters underlying weakly oxygenated bottom waters (*120–124*). Both U and Re enrichments primarily occur via diffusion from the water column into reducing sediments across the sediment-water interface (*126–128*). U and Re thus behave similarly to changes in depositional redox state, however Re has a higher sensitivity to weakly reducing conditions than U, and is also efficiently sequestered in the sediment under dysoxic conditions characterized by O_2 penetration depths of <1 cm below the sediment-water interface (*119–121*, *128*). Enhanced U and Re enrichments can take place under anoxic conditions characterized by low dissolved H_2S, whereas high dissolved H_2S is a prerequisite for high authigenic Mo enrichments (*129*). Mild authigenic Mo enrichments may also occur in sulfidic porewaters beneath weakly oxic to anoxic ferruginous water column conditions, and the magnitude of enrichment scales with sulfide availability (*120–122*). Importantly, widespread anoxic (and for Mo, euxinic) water column conditions will necessarily result in substantial redox sensitive trace element depletion in the global ocean or restricted basin, leading to muted authigenic enrichments (*130*, *131*).

We characterize changes in water column and pore water redox conditions throughout each core based on an integrated assessment of Fe speciation and redox sensitive trace element systematics (Table S2).

Table S2. Criteria used for the core data presented herein to identify water column and pore water paleoredox conditions

Water column redox state	Redox state of pore waters and/or sediment-water interface (SWI)	Criteria	Justification and specific core details
**dominantly oxic**	oxic SWI	predominance of Fe_{HR}/Fe_T <0.38, negligible Mo_{EF}, negligible U_{EF}, low Re_{EF}, low to moderate Re/Mo, low Re/U	Negligible enrichment in highly reactive iron or redox sensitive trace elements support oxic depositional conditions.
**dysoxic**	dysoxic SWI	predominance of Fe_{HR}/Fe_T <0.38, negligible Mo_{EF}, negligible U_{EF}, slightly elevated Re_{EF}, elevated Re/Mo, elevated Re/U	Negligible enrichment in highly reactive iron, Mo or U support oxic water column conditions. Minor Re enrichments relative to Mo and U support dysoxic SWI conducive to minor sedimentary Re retention.
**oxic to ferruginous**	oxic to ferruginous SWI	cyclic Fe_{HR}/Fe_T with values above and below 0.38, Fe_{py}/Fe_{HR} <0.6, negligible Mo_{EF}, negligible U_{EF}, negligible Re_{EF}, moderate to high Re/Mo, low Re/U	cyclicity in Fe speciation data is most prominently exhibited in BR05-DD01 (440-470m), and may suggest repetitive deepening of the redoxcline. No Mo enrichment due to low sulfide availability. Re and (to a greater degree) U may be lost from pore waters due to intermittent oxic conditions at the SWI.
**ferruginous**	sulfidic pore waters and ferruginous SWI	Fe_{HR}/Fe_T >0.38, Fe_{py}/Fe_{HR} >0.6, generally moderate Mo_{EF}, low to moderate-high U_{EF}, moderate to high Re_{EF}, low to moderate Re/Mo, low to high Re/U	Values of Mo_{EF} are variable (highest values occur in core Wallara-1), with enrichments dictated by sulfide availability. Re_{EF} are variable but are occasionally elevated despite low U_{EF} (e.g. SCYW-79-1A), which suggests that the SWI was not consistently sufficiently reducing for appreciable U drawdown.
**euxinic**	sulfidic pore waters	Fe_{HR}/Fe_T >0.38, Fe_{py}/Fe_{HR} >0.6, high Mo_{EF}, Moderate-high U_{EF}, high Re_{EF}, low Re/Mo, low to moderate Re/U	The combination of Fe_{py}/Fe_{HR} >0.6, high Mo_{EF} and low Re/Mo, is a robust indicator of efficient drawdown of Mo under elevated water column H_2S.

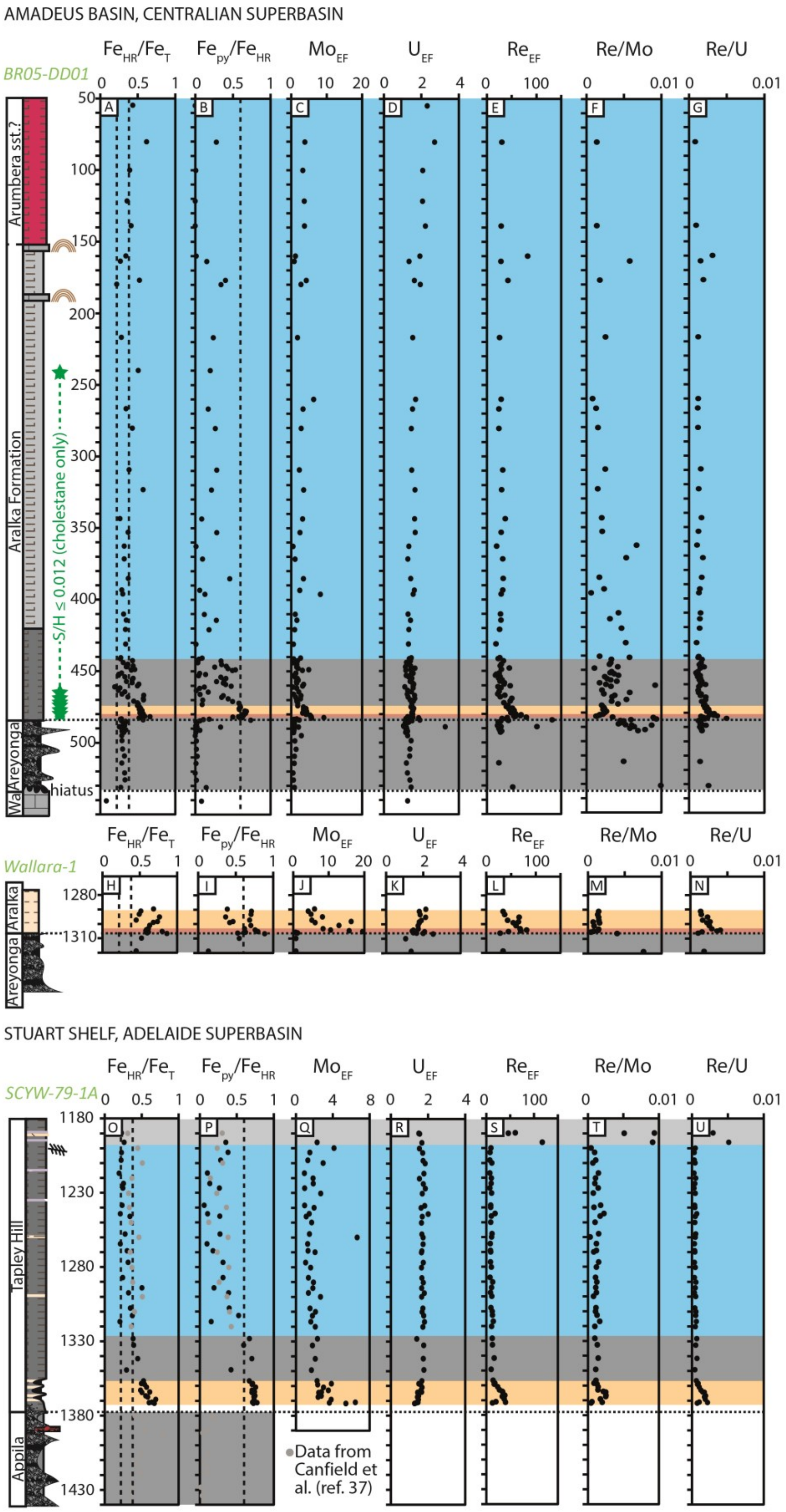

Fig. S4. Water column and pore water redox assessment for drill cores of Australia based on integrated Fe speciation data and redox sensitive trace element enrichments (Table S2). Redox data presented stratigraphically. See main text figures 2–3 for keys to lithostratigraphy. See Fig. S5 for reference to color banding by redox determination. Positions of samples analyzed for biomarker data (*2*) indicated by green stars. S/H – sterane/hopane ratio.

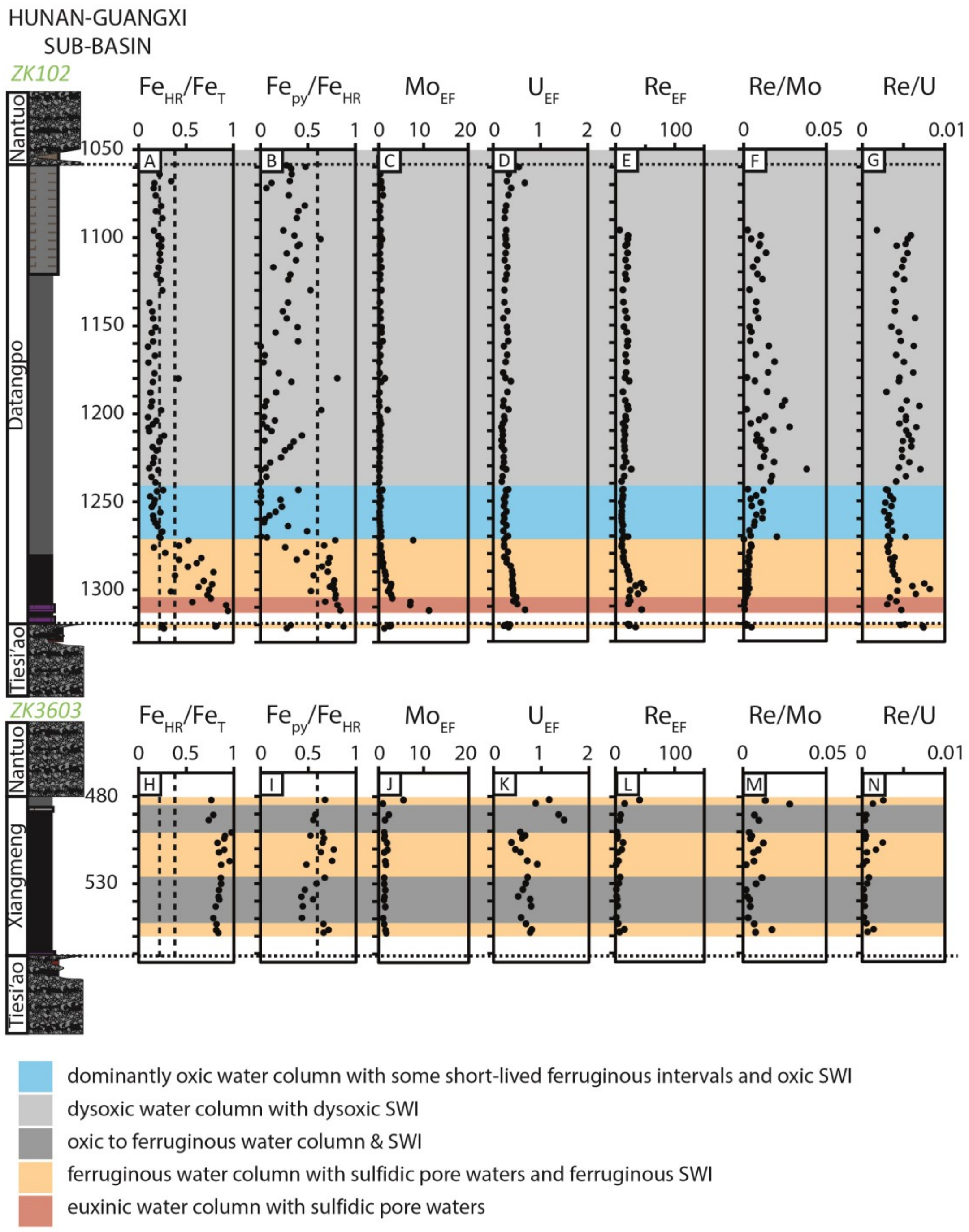

Fig. S5. Water column and pore water redox assessment for drill cores of South China based on integrated Fe speciation data and redox sensitive trace element enrichments (Table S2). Redox data presented stratigraphically. See main text figures 2–4 for keys to lithostratigraphy.

Long-term trends in paleoredox data and the composition of weathered source material

Long-term intra-basinal trends in elemental data may simply reflect long-term changes to weathering style or the source of weathered material within a catchment (*114*). This may be partially tractable in studies that resolve consistent trends between multiple sections from different depositional basins (*114*). However, the Cryogenian non-glacial interval may represent a specific case where all catchment areas and regional depositional environments were influenced by broadly similar long-term trends in both weathering regimes and depositional rate (albeit with variability in magnitudes controlled by differences in paleolatitude and regional tectonics). If long-term trends in trace metal enrichments are primarily related to long-term shifts in source composition, this may be reflected in corresponding trends in the concentrations of detrital elements used for normalization. However, as noted above, none of the analyzed cores show notable systematic unidirectional trends in Al concentration throughout the non-glacial interval. The correlation of trends observed between proxies that rely upon detrital normalization (e.g., U_{EF}) and those that are independent of detrital normalization (Fe-speciation) therefore strongly supports a common driver that is most parsimoniously related to changing depositional paleoredox conditions, which is further supported by the observed correlation between interpreted paleoredox conditions and P-speciation data (e.g., Figs. 2–4, see below).

Critical assessment of P speciation data

One possible complication when interpreting P speciation data is the potential for late stage diagenetic transformation of carbonate fluorapatite (P_{auth}) to more crystalline P-bearing phases (*76*, *132*). The resulting crystalline phases would be operationally extracted as P_{det}, thereby reducing the P_{reac} pool relative to the primary depositional value and increasing the corresponding ratio of TOC/P_{reac}. Previous studies have emphasized the likelihood for a high postglacial influx of detrital P, enhanced by the weathering of P-enriched Tonian LIPs (*3*, *133*). Indeed, we do see a minor and short-lived increase in P_{Tot} and P_{Tot}/Al in the immediate aftermath of the Sturtian glaciation. Detrital P accounts for the largest P pool in the majority of sampled cores, but it is notably elevated in cores from both Australian basins relative to the Nanhua Basin (mean P_{det} relative to summed extracted P: SCYW-79-1A = 72.3%, BR05-DD01 = 75.5%, Wallara-1 = 86.2%, ZK102 = 55.7%, ZK3603 = 16.6%). The relative contribution of P_{det} between and within cores is intuitive when considering the relative paleodepth and stratigraphic distance of sampled sediments from the underlying Sturtian diamictite. Specifically, the highest mean values of P_{det} occur in shallower cores, and are found in the immediate aftermath of Sturtian deglaciation (SCYW-79-1A, BR05-DD01 and Wallara-1) and with

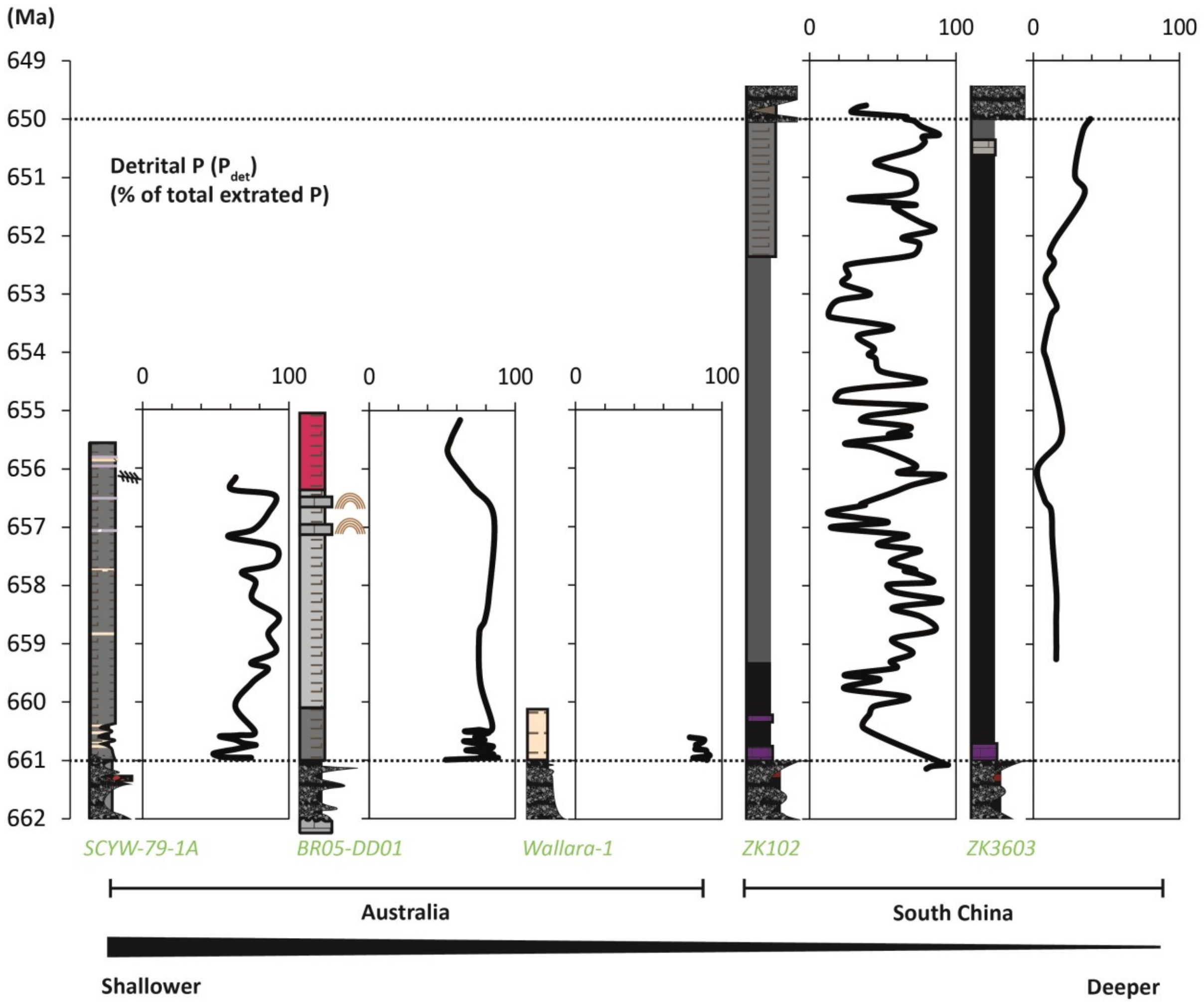

Fig. S6. Detrital P (P_{det}) as a percentage of the total P extracted during the sequential P extraction. Lithostratigraphic columns are schematically shown with interpreted relative paleodepth and depositional duration. See Figs. 2–4 for keys to lithostratigraphic and sedimentological symbols. The relative duration of deposition within Age Model A is shown to the left.

progressive shallowing immediately prior to Marinoan re- glaciation (ZK102 and ZK3603) (Figs. 2–4, S6). In contrast to the Aralka and Tapley Hill formations, the Datangpo and Xiangmeng formations in cores ZK102 and ZK3603 likely represent more continuous deposition throughout the Cryogenian non-glacial (Figs. 1F, S6). The P_{det} contribution in the deepest core (ZK3603) is substantially lower relative to all other cores, which is an expected consequence of distance from the detrital P source throughout deposition (Fig. S6).

We note that there is no significant correlation between P_{auth} and P_{det} in any of our cores (Fig. S7A). However, we also note that, with the exception of Wallara-1, there is no significant positive correlation between P_{det} and Al (Fig. S7B). If high values of TOC/P_{reac} observed in some euxinic/

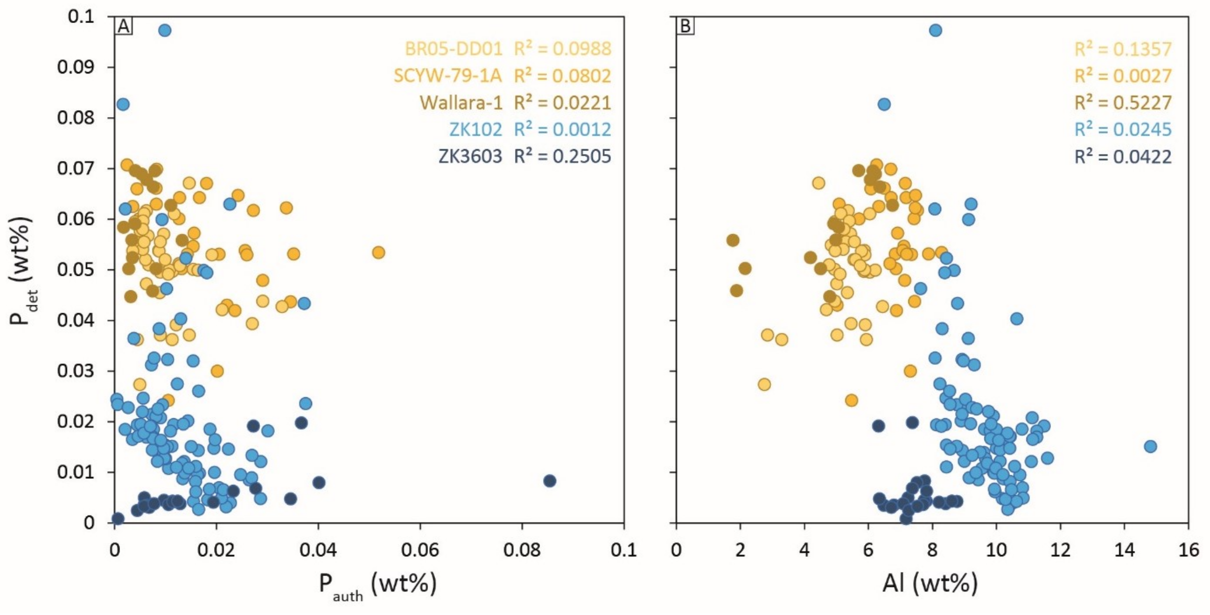

Fig. S7. Relationships between detrital P, authigenic P and Al. (A) P_{det} vs. P_{auth} and **(B)** P_{det} vs. Al for all samples analyzed for P speciation. Yellow-orange and blue data points are from Australian and South China samples, respectively (see key in A).

sulfidic samples have been artificially elevated via diagenetic transformation of P_{auth} to P_{det}, we may expect to see a correlation between positive excursions in P_{det}/Al and euxinic/sulfidic intervals. However, no clear and consistent correlation is evident in our cores (Fig. S8). Therefore, while diagenetic recrystallization is likely to have reduced P_{reac} in some samples, this has not consistently altered TOC/P_{reac} to higher values in sediments immediately following Sturtian deglaciation. We further note that the majority of euxinic/sulfidic samples from South China (in addition to some ferruginous samples in ZK3603) have TOC/P_{Tot} greater than the Redfield ratio, where P_{Tot} is the concentration of P in the bulk digest solution. These high values are most clearly displayed in ZK3603, where the contribution by P_{det} is minimal relative to P_{reac} (and especially P_{Fe}). Values of TOC/P_{Tot} > 106 provide unequivocal support for P recycling from sulfidic porewaters or under euxinic water column conditions, regardless of the extent of diagenetic transformation of P_{auth} to P_{det}. By contrast, lower values of TOC/P_{Tot} in Australian cores reflect a proportionally larger P_{det} contribution that results from the elevated supply of P to both the Amadeus Basin and Adelaide Superbasin (attested by high P_{Tot}/Al), relative to the Nanhua Basin.

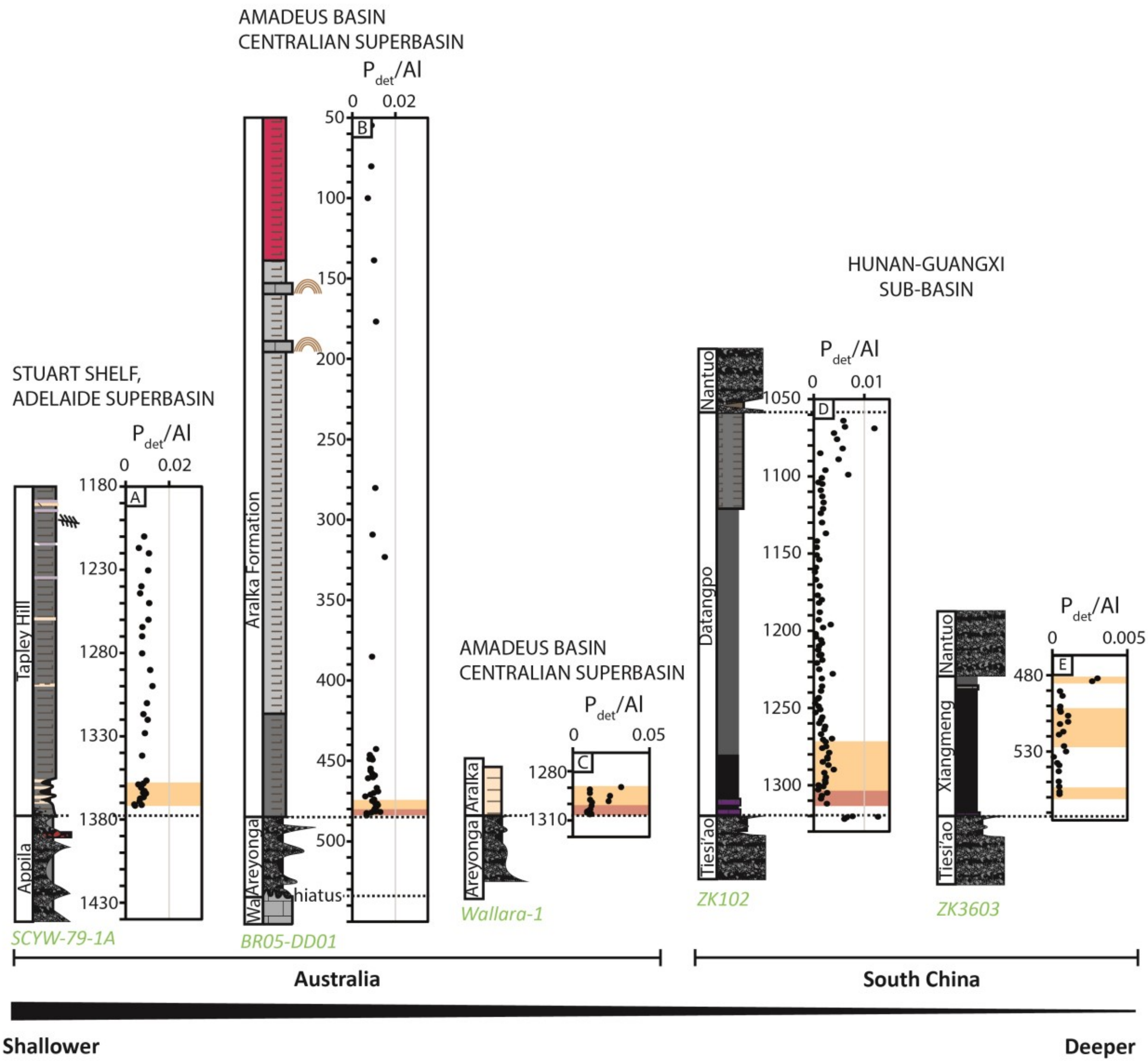

Fig. S8. The ratio of P_{det}/Al for all samples analyzed for P speciation. All drill cores are to scale. Horizontal red bands indicate intervals of euxinic water column conditions and horizontal yellow bands indicate intervals of ferruginous water column conditions with sulfidic pore waters based on interpreted Fe speciation and redox sensitive trace element concentrations (see Figs. S3–S5). Relative paleodepth is interpreted from core sedimentology and inferred relative position on the shelf. Note that Australian cores are not bracketed by overlying diamictite, and core completeness does not correlate directly with differences in accommodation space.

The Redfield ratio and changing C/P through time

Changes to mean global temperature, marine phosphate concentration and marine paleoredox conditions are likely to have resulted in deviation of C:N:P from the Redfield ratio throughout geologic time (e.g., *46*, *134*, *135*). In particular, decreasing mean global temperature and increasing marine phosphate availability throughout the Phanerozoic may have decreased C/P_{org} of planktonic

biomass (e.g., ref. *134* and references therein). Similarly, gradual global cooling during the non-glacial Cryogenian could potentially have resulted in a corresponding decrease in C/P_{org} of planktonic biomass. However, in the immediate aftermath of Sturtian deglaciation, when C/P_{org} may have been at its highest, shelf-slope cores in Australia (e.g., Wallara-1) show maximum C/P_{reac} = 315, and shelf-slope cores in South China (e.g., ZK102) yield maximum C/P_{reac} = 770. These values greatly exceed modelled maximum values of Phanerozoic C/P_{org} (*134*), and are elevated relative to C/P_{org} hypothesized for P-limited conditions in redox stratified Proterozoic oceans (C/P_{org} = 300/1; *46*). Together, high non-glacial C/P_{reac} most parsimoniously represent a degree of bioavailable P recycling from sediments in the immediate post-Sturtian Cn1 interval. The most efficient P recycling is recorded in shelf-slope and basinal cores of South China, as emphasized in the main text. Maintenance of high C/P_{reac} (mean = 353) in our deepest core (ZK3603), supports a degree of bioavailable P recycling from sulfidic sediment pore waters in some anoxic basinal environments throughout the non-glacial interval, even if C/P_{org} of primary biomass remained elevated relative to the Redfield ratio.

Table S3. Radiometric ages used in construction of the global age model framework

Age (Ma)	Details	Reference
**639.29 ± 0.26 (0.31) [0.75]**	Zircon U-Pb CA-ID-TIMS age of tuff deposit interbedded with the Ghaub glacial diamictite (~15m below the base of the Keilberg cap carbonate), Duurwater section, northern Namibia (sample DW-1). Middle of three ash beds. Nine single grain analyses (MSWD = 2.6, n = 9)	(*21*)
**651.2 ± 3.3 Ma**	Zircon U-Pb laser ablation inductively coupled plasma mass spectrometry (LA-ICP-MS) age of a 3 cm-thick tuff deposit within the upper Datangpo Formation, 0.4 m below the unconformable boundary with the overlying Marinoan diamictite, Gucheng section, western Hubei Province (sample DTP-27). Eighteen single grain analyses (of 39 valid analyses) (MSWD = 0.36).	(*20*)
**651.69 ± 0.64 (0.73) [0.99] Ma**	U-Pb zircon CA-ID-TIMS age from a lenticular bed of laminated light-grey siltstone within dolostone of the upper Thorndike submember, 15m below the base of the Wildrose submember of the South Park Member of the Kingston Peak Formation, Panamint Range, Death Valley, California (sample L1502). Interpreted as a maximum depositional age. MSWD=1.0, n=4 of 5.	(*19*)
**657.17 ± 0.27 (0.37) [0.78] Ma**	U-Pb zircon CA-ID-TIMS age of a mudstone deposit interpreted as a devitrified airfall tuff bed in the middle siltstone unit of the Datangpo Formation, Maopingdong section, western Hunan province, China (sample FDM14-14). Interpreted as a depositional age. MSWD=1.3, n=7.	(*17*)

**658.8 ± 0.50 (0.54) [1.49] Ma**	U-Pb zircon CA-ID-TIMS age of a tuff bed in the basal Datangpo Formation cap dolostone, Jiangjunshan section, eastern Guizhou province, China (sample 17LSJ-17). MSWD=1.4, n=9 of 11.	(*94*)
**658.97 ± 0.22 (0.33) [0.76] Ma**	U-Pb zircon CA-ID-TIMS age of a tuff bed in the lower black shale unit of the Datangpo Formation, Xiaozhai section, western Hunan province, China (sample FDM14-13). Interpreted as a depositional age. MSWD=1.3, n=6.	(*17*)
**659 ± 4.5 Ma**	Re-Os age from the cap carbonate in the basal Tayshir Fm, 1.2m above the contact with the Maikhan-Uul diamictites, Taishir locality, Mongolia. Initial $^{187}Os/^{188}Os$=0.60±0.01, MSWD=0.67, n=6.	(*95*)
**660.6 ± [3.1] 3.9 Ma**	Re-Os age of a black shale unit interbedded with diamictites of the Tiesi-ao Formation, Guangzidong section, northeast Guizhou, China (sample F1408-15.6). Interpreted as a depositional age. Initial $^{187}Os/^{188}Os$=1.55±0.05, MSWD=0.92.	(*17*)
**660.7 ± 1.1 Ma**	U-Pb zircon CA-ID-TIMS age of a mudstone deposit interpreted as a devitrified airfall tuff bed in the lower black shale unit of the Datangpo Formation, Xiaozhai section, western Hunan province, China (sample FDM14-12). Interpreted as a maximum age.	(*17*)
**660.8 ± 1.5 Ma**	U-Pb zircon CA-ID-TIMS age of a mudstone deposit interpreted as a devitrified airfall tuff bed in the lower Datangpo Formation (siltstone), Jiangjunshan section, northeast Guizhou province, China (sample FDM14-19). Interpreted as a maximum age.	(*17*)
**660.98 ± 0.18 (0.31) [0.74] Ma**	U-Pb zircon CA-ID-TIMS age of a mudstone deposit interpreted as a devitrified airfall tuff bed in the lower black shale unit of the Datangpo Formation, Gaozeng section, southeast Guizhou province, China (sample 17-	(*17*)

	GZGZ01). Interpreted as a depositional age. MSWD=1.5, n=7.	
**662.1 ± 0.8 Ma**	U-Pb zircon CA-ID-TIMS age of a mudstone deposit interpreted as a devitrified airfall tuff bed in the lower Datangpo Formation, Xiaozhai section, western Hunan province, China (sample FDM14-11). Interpreted as a maximum age.	(*17*)
**662.4 ± 3.9 Ma**	Re-Os age of organic-rich (>0.5 wt%) micritic limestone of the lower Twitya Formation of the Hay Creek Group near Mountain River, Mackenzie Mountains, Northwest Territories, Canada. Initial $^{187}Os/^{188}Os$=0.54±0.01, MSWD=1.9, n=7.	(*16*)
**662.7 ± 6.2 Ma**	U-Pb LA-ICP-MS age of zircons from a tuff bed in the lower black shale unit of the Datangpo Formation, Jiangjunshan section, northeast Guizhou province, China (sample JJS-T). MSWD = 7.8, n = 42. Interpreted as a depositional age.	(*136*)
**662.9 ± 4.3 Ma**	Weighted mean ^{207}Pb-^{206}Pb zircon ID-TIMS age of a tuff bed in the lower Datangpo Formation (within Mn-carbonate-rich layer), Zhailanggou section, easter Guizhou province, China (sample ZLG-5). Methodology employed for zircon abrasion uncertain (possibly air-abrasion). MSWD=1.24, n=6	(*137*)
**663.03 ± 0.11 (0.33) [0.76] Ma**	^{206}Pb-^{238}U zircon CA-ID-TIMS age from sixteen single grain analyses. Zircons derive from a ca. 3 cm-thick tuff within uppermost Sturtian diamictites (Wilyerpa Formation) of the Umberatana Group (stratigraphically above the Burra Group and below the Wilpena Group) west of Copley township (sample P1634). MSWD=0.52, n=16.	(*18*)

Table S4. Analytical Data 1: Major and trace element concentrations, Fe speciation, total organic carbon (TOC) concentrations, and pyrite sulfur ($\delta^{34}S_{py}$) and organic carbon ($\delta^{13}C_{org}$) isotopes. Depth (m) indicates sample position relative to drill core datum. BD = below detection; ND = not determined. Carbonate samples are denoted by italicized text.

**ZK102** core													
**ID**	**Depth (m)**	**Al (wt%)**	**Fe_T (wt%)**	**Fe_{carb} (wt%)**	**Fe_{ox} (wt%)**	**Fe_{mag} (wt%)**	**Fe_{py} (wt%)**	**TOC (wt%)**	**$\delta^{34}S_{py}$ (‰)**	**$\delta^{13}C_{org}$ (‰)**	**Mo (ppm)**	**Re (ppb)**	**U (ppm)**
Tiesi'ao Formation													
DTP-1	1322	7.627	1.821	0.311	0.031	0.009	0.134	0.058	53.65	-28.55	1.28	6.24	0.83
DTP-2	1321	9.125	4.705	0.441	0.017	0.017	3.346	0.078	59.09	-29.28	3.05	5.09	0.69
DTP-3	1320.5	6.495	2.927	0.653	0.017	0.009	1.721	0.072	57.34	-29.49	2.37	3.64	0.78
DTP-4	1320.3	8.073	3.492	0.571	0.019	0.009	0.278	0.065	57.99	-29.88	2.60	4.12	0.79
Datangpo Formation													
DTP-5	*1319.6*	*3.452*	*2.162*	*0.781*	*0.031*	*0.024*	*1.122*	*1.189*	*56.65*	*-32.37*	*0.52*	*17.60*	*0.52*
DTP-6	*1319*	*7.182*	*2.189*	*0.862*	*0.017*	*0.008*	*0.735*	*0.315*	*54.38*	*-32.40*	*3.64*	*16.83*	*0.69*
DTP-7	*1318*	*2.328*	*2.191*	*0.846*	*0.049*	*0.064*	*0.997*	*1.615*	*63.90*	*-32.30*	*0.48*	*15.16*	*0.43*
DTP-8	*1316*	*0.521*	*1.462*	*0.866*	*0.051*	*0.029*	*0.085*	*2.641*	*57.20*	*ND*	*BD*	*15.61*	*BD*
DTP-10	1312	9.000	3.332	0.439	0.037	0.011	2.648	2.073	49.21	-32.78	13.65	9.62	2.02
DTP-11	*1310*	*2.517*	*2.377*	*0.924*	*0.040*	*0.038*	*0.832*	*1.372*	*61.89*	*-32.66*	*BD*	*19.30*	*0.53*
DTP-12	1308.8	10.000	3.048	0.472	0.037	0.015	2.291	2.016	57.89	-32.56	9.47	5.17	1.70
DTP-13	1307	8.936	4.202	0.661	0.053	0.038	1.626	2.188	61.14	-32.50	8.55	5.30	1.28
DTP-14	1305	8.768	4.199	0.575	0.039	0.057	2.526	1.782	64.66	-32.42	3.63	4.83	1.43
DTP-15	1303	9.428	3.619	0.451	0.036	0.052	2.086	1.869	57.41	-32.43	3.49	8.63	1.33
DTP-16	1301	9.629	3.847	0.511	0.040	0.059	0.699	1.772	63.55	-32.12	2.74	5.79	1.35
DTP-17	1300	9.145	3.874	0.528	0.039	0.060	2.248	2.015	59.64	-32.17	2.93	10.55	1.28
DTP-18	1298.4	8.398	3.912	0.579	0.039	0.048	1.808	3.413	48.31	-31.80	2.98	6.89	1.13
DTP-19	1297	8.901	3.569	0.553	0.041	0.045	2.131	3.223	47.14	-31.70	3.32	9.31	1.23
DTP-20	1295	8.921	3.901	0.508	0.039	0.048	2.083	2.530	53.39	-31.62	1.88	5.17	1.19
DTP-21	1292	9.188	3.660	0.511	0.040	0.065	0.785	2.327	54.92	-31.64	1.94	4.98	1.26
DTP-22	1290	9.118	3.749	0.717	0.046	0.092	2.101	1.853	54.04	-31.62	1.66	4.54	1.22
DTP-23	1287	8.418	3.940	0.630	0.034	0.050	1.332	1.303	36.48	-31.84	1.05	3.78	1.03
DTP-24	1285	8.433	3.387	0.505	0.033	0.051	1.487	1.082	29.41	-32.02	1.16	3.03	0.81
DTP-25	1283	9.030	4.383	0.969	0.038	0.140	0.712	0.566	28.30	-31.87	0.86	2.90	0.86
DTP-26	1282	8.554	4.567	0.686	0.036	0.094	2.209	0.476	33.24	-31.23	0.84	2.62	0.67
DTP-27	1279	8.535	3.161	0.327	0.025	0.102	0.428	0.181	31.55	-31.00	0.81	2.84	0.89
DTP-28	1276	8.760	3.554	0.315	0.025	0.081	0.148	0.324	41.46	-31.67	0.43	2.10	0.68
DTP-29	1275	9.238	3.905	0.433	0.026	0.083	1.111	0.165	53.82	-30.70	0.52	2.33	0.72
DTP-30	1272	8.129	6.796	0.614	0.034	0.105	2.822	0.277	27.46	-31.10	8.43	2.12	0.62
DTP-31	1270.5	11.111	3.812	0.598	0.037	0.175	0.059	0.196	14.86	-30.66	0.27	5.50	1.04
DTP-32	1270	8.922	3.756	0.687	0.030	0.109	0.005	0.157	ND	-30.30	0.67	2.92	0.93
DTP-33	1267	9.609	3.961	0.376	0.029	0.100	0.490	0.353	70.40	-31.19	0.71	2.51	0.69
DTP-34	1264	9.898	4.469	0.456	0.035	0.148	0.264	0.174	9.45	-30.65	0.45	2.90	0.93
DTP-35	1262	9.753	4.094	0.539	0.033	0.126	0.028	0.178	-5.17	-30.70	0.37	2.47	0.70
DTP-36	1260	9.541	3.023	0.331	0.027	0.097	0.023	0.379	18.42	-32.07	0.20	2.33	0.75
DTP-37	1258	10.165	3.956	0.369	0.034	0.143	0.058	0.216	-5.35	-31.05	0.32	2.50	0.77
DTP-38	1256	10.225	3.625	0.545	0.034	0.117	0.134	0.202	47.53	-31.02	0.22	2.55	0.96
DTP-39	1253	10.511	3.880	0.249	0.030	0.140	0.122	0.148	16.27	-30.50	0.56	2.79	0.79
DTP-40	1251	9.657	5.580	0.684	0.038	0.175	0.002	0.150	16.96	-30.98	0.22	2.26	0.80
DTP-41	1249	9.906	4.743	0.412	0.040	0.184	0.170	0.178	17.78	-31.09	0.65	2.85	0.76
DTP-42	1247	10.385	3.626	0.261	0.033	0.146	0.002	0.162	15.74	-30.83	0.41	3.10	0.91
DTP-43	1244	10.208	4.330	0.627	0.041	0.162	0.001	0.136	27.53	-30.61	0.24	2.86	0.92
DTP-44	1243.5	9.939	4.848	0.506	0.047	0.204	0.502	0.171	16.69	-30.74	1.15	3.08	1.06
DTP-45	1239	9.486	4.184	0.572	0.036	0.131	0.003	0.135	1.91	-30.80	0.14	2.35	0.57
DTP-46	1236	11.486	4.413	0.404	0.035	0.129	0.038	0.132	-6.41	-30.24	0.23	4.04	0.76
DTP-47	1232	10.308	5.600	0.690	0.052	0.408	0.005	0.126	-6.96	-30.15	0.17	6.67	0.94
DTP-48	1231	11.253	4.675	0.342	0.030	0.121	0.030	0.158	-1.31	-31.36	0.33	3.44	0.77
DTP-49	1228	10.633	4.868	0.463	0.038	0.170	0.076	0.147	50.08	-31.09	0.25	4.59	0.80
DTP-50	1225	10.787	3.813	0.316	0.031	0.133	0.134	0.160	51.88	-30.88	0.32	3.88	0.80

DTP-51	1221	11.092	4.808	0.445	0.041	0.180	0.232	0.175	39.55	-31.70	0.32	4.11	0.85
DTP-52	1219	10.813	4.636	0.285	0.034	0.153	0.215	0.145	51.75	-31.46	0.39	3.99	0.67
DTP-53	1216	10.793	4.913	0.492	0.042	0.158	0.376	0.147	41.75	-31.27	0.47	3.66	0.74
DTP-54	1215.5	11.597	4.396	0.767	0.033	0.133	0.041	0.168	1.64	-31.34	0.39	4.11	0.68
DTP-55	1212.5	11.254	5.251	0.572	0.049	0.166	0.611	0.190	35.17	-31.47	0.54	4.32	0.77
DTP-56	1210	10.736	4.658	0.291	0.034	0.138	0.061	0.134	-1.21	-31.51	0.21	3.84	0.72
DTP-57	1208	10.566	4.233	0.280	0.025	0.105	0.029	0.111	-7.65	-31.18	0.15	4.10	0.62
DTP-58	1206	10.430	5.541	0.577	0.033	0.193	0.018	0.118	0.81	-30.99	0.74	3.25	0.72
DTP-59	1204	11.135	3.710	0.410	0.028	0.145	0.104	0.159	51.63	-31.25	0.51	4.80	0.90
DTP-60	1202	10.831	4.145	0.204	0.022	0.160	0.015	0.101	-2.33	-30.33	0.34	4.45	0.83
DTP-61	1198	10.625	5.219	0.272	0.028	0.144	0.797	0.597	20.32	-33.39	2.85	5.46	1.15
DTP-62	1196	9.800	5.951	0.405	0.047	0.285	0.037	0.105	-8.70	-30.14	0.21	4.79	0.69
DTP-63	1193	9.306	5.731	0.535	0.035	0.193	0.052	0.100	-14.55	-30.17	0.15	3.73	0.69
DTP-64	1188	14.817	5.055	0.320	0.032	0.174	0.105	0.097	62.40	-29.39	0.31	4.39	1.49
DTP-65	1182	9.715	4.335	0.264	0.026	0.150	0.214	0.113	-17.33	-30.75	0.78	5.39	1.21
DTP-66	1180	9.359	7.276	0.353	0.035	0.187	2.471	0.093	21.83	-29.31	1.75	3.63	0.80
DTP-67	1177	10.338	5.367	0.419	0.048	0.248	0.170	0.084	46.50	-28.94	0.30	4.47	0.72
DTP-68	1171	10.427	4.660	0.288	0.026	0.156	0.016	0.130	-2.61	-30.48	0.25	4.63	0.92
DTP-69	1167	10.110	4.345	0.490	0.036	0.183	0.035	0.101	-13.55	-29.68	0.56	4.16	1.01
DTP-70	1162	10.180	4.534	0.208	0.030	0.207	0.002	0.107	3.44	-29.39	0.32	4.82	0.77
DTP-71	1159	10.353	4.050	0.174	0.031	0.170	0.246	0.516	3.45	-32.75	1.31	5.20	1.11
DTP-72	1154	10.285	3.798	0.204	0.036	0.202	0.084	0.127	7.97	-29.56	0.96	4.67	1.05
DTP-73	1151	9.977	4.166	0.250	0.029	0.168	0.287	0.317	32.25	-31.91	0.91	3.41	0.96
DTP-74	1146	10.464	4.449	0.230	0.034	0.229	0.190	0.128	34.40	-30.11	0.54	4.83	0.75
DTP-75	1142	9.952	4.214	0.247	0.033	0.186	0.144	0.210	16.79	-30.95	0.54	3.97	1.00
DTP-76	1137	9.936	4.294	0.163	0.025	0.157	0.141	0.135	37.38	-30.16	0.41	3.17	0.78
DTP-77	1130	9.375	5.072	0.394	0.042	0.165	0.672	0.274	31.73	-31.98	0.81	2.82	0.74
DTP-78	1124	10.418	3.880	0.429	0.036	0.167	0.264	0.278	26.85	-31.50	0.41	4.64	0.91
DTP-79	1121	10.113	3.953	0.336	0.028	0.155	0.239	0.120	29.98	-29.91	0.47	4.01	0.97
DTP-80	1117	10.000	4.955	0.624	0.050	0.225	0.143	0.207	27.59	-30.85	0.85	4.85	1.00
DTP-81	1113	9.832	4.915	0.446	0.042	0.216	0.424	0.194	29.52	-31.28	0.48	4.00	0.79
DTP-82	1109	9.820	5.265	0.504	0.054	0.289	0.327	0.169	29.29	-30.35	0.35	4.74	0.86
DTP-83	1105	10.419	4.731	0.388	0.050	0.255	0.444	0.292	18.12	-31.53	0.46	4.31	1.03
DTP-84	1104	10.343	4.463	0.345	0.039	0.181	0.395	0.232	28.24	-31.25	0.49	4.78	0.91
DTP-85	1101	10.205	5.319	0.223	0.039	0.213	0.819	0.123	27.65	-29.77	1.09	5.08	0.92
DTP-86	1099	10.071	4.738	0.388	0.041	0.200	0.353	0.210	30.50	-30.60	0.50	5.16	0.87
DTP-87	1096	9.212	4.971	0.347	0.042	0.219	0.195	0.156	31.60	-30.09	0.59	1.49	0.84
DTP-88	1089	8.277	4.701	0.499	0.043	0.180	0.450	0.158	29.18	-30.49	0.46	BD	0.67
DTP-89	1085	8.779	5.005	0.263	0.041	0.243	0.365	0.154	31.56	-30.46	0.25	BD	0.75
DTP-90	1082	8.438	4.572	0.331	0.045	0.200	0.507	0.275	16.92	-31.33	0.40	BD	0.77
DTP-91	1076	8.677	5.276	0.369	0.048	0.252	0.284	0.205	28.83	-30.70	1.17	BD	0.95
DTP-92	1072	8.302	4.601	0.435	0.045	0.200	0.046	0.202	32.34	-30.76	0.90	BD	1.05
DTP-93	1069	8.079	4.580	0.499	0.037	0.139	0.090	0.175	29.72	-30.51	0.56	BD	1.80
DTP-94	1068	8.103	5.416	0.904	0.051	0.327	0.575	0.170	30.68	-29.76	0.55	BD	0.79
DTP-95	1064	8.439	4.847	0.481	0.036	0.195	0.350	0.163	26.51	-29.73	0.36	BD	0.93
DTP-96	1061	8.387	4.954	0.469	0.031	0.185	0.329	0.213	26.66	-30.13	0.54	BD	1.02
DTP-97	1060	8.970	4.605	0.376	0.022	0.120	0.469	0.214	27.41	-30.38	0.43	2.05	1.62
Nantuo Formation													
DTP-98	1059	8.229	4.359	0.441	0.027	0.148	0.234	0.146	27.99	-30.18	0.19	BD	0.59
DTP-99	1057	8.999	4.142	0.275	0.024	0.139	0.047	0.135	-17.05	-29.86	0.14	BD	0.68
DTP-100	1055	9.242	4.293	0.253	0.020	0.129	0.017	0.123	7.64	-29.59	0.19	BD	0.75
DTP-101	1054	8.553	4.120	0.301	0.027	0.125	0.040	0.106	24.46	-28.92	0.17	BD	0.56

ZK3603 core

All $\delta^{13}C_{org}$ data originally published in Peng et al. (ref. 14)

ID	Depth (m)	Al (wt%)	Fe_T (wt%)	Fe_{carb} (wt%)	Fe_{ox} (wt%)	Fe_{mag} (wt%)	Fe_{py} (wt%)	TOC (wt%)	$\delta^{34}S_{py}$ (‰)	$\delta^{13}C_{org}$ (‰)	Mo (ppm)	Re (ppb)	U (ppm)
Xiangmeng Formation													
94	482	6.319	2.476	0.446	0.080	0.089	1.277	1.579	-9.82	*-32.20*	4.71	6.32	2.44
92	484	7.377	1.628	0.383	0.039	0.120	0.470	1.456	-1.80	*-32.30*	1.01	2.84	2.17
87	490.5	6.793	2.284	0.369	0.370	0.025	1.032	1.844	19.85	*-32.40*	2.16	1.51	3.08
84	493.5	7.243	2.228	0.289	0.418	0.022	0.911	1.631	24.10	*-32.40*	1.42	1.36	3.55
78	500.5	7.810	3.360	0.579	0.541	0.018	2.139	4.276	61.52	*-31.40*	1.38	0.51	1.45
74	502.5	6.483	2.744	0.516	0.654	0.017	1.305	3.767	62.58	*-32.70*	1.23	0.65	1.43
72	504	7.741	2.674	0.398	0.388	0.015	1.599	3.900	62.81	*-32.60*	1.36	0.63	1.54
70	506.5	7.755	2.235	0.359	0.270	0.012	1.208	3.614	60.70	*-32.80*	2.00	2.46	0.98
66	510.5	7.515	2.916	0.452	0.143	0.009	2.013	3.927	62.59	*-32.70*	2.09	1.95	1.14
64	512	7.090	2.539	0.336	0.435	0.014	1.359	3.840	63.56	*-32.50*	1.23	0.79	1.34
59	517	6.343	4.094	0.382	0.575	0.017	2.937	3.828	62.56	*-32.70*	1.28	0.84	1.49
57	519	6.713	2.903	0.691	0.598	0.013	1.215	4.625	63.39	*-32.30*	1.52	0.29	2.04
49	526.5	7.814	2.841	0.470	0.314	0.016	1.651	4.792	64.23	*-32.30*	1.36	1.55	1.86
45	530	7.375	2.967	0.547	0.490	0.025	1.508	4.185	65.29	*-32.30*	1.29	1.03	1.66
41	533.5	7.177	3.240	0.938	0.507	0.016	1.272	4.069	62.75	*-32.40*	1.46	0.29	1.47
37	537.5	7.259	3.652	0.872	0.856	0.020	1.317	3.750	60.15	*-32.80*	1.33	0.36	1.26
35	539	7.708	2.851	0.375	0.686	0.021	1.331	4.120	57.98	*-32.60*	1.31	0.55	1.96
31	543	7.530	3.000	0.595	0.734	0.019	1.079	3.676	57.68	*-32.90*	1.52	0.68	1.98
24	549.6	8.406	2.938	0.576	0.708	0.019	1.006	3.313	48.72	*-32.90*	1.22	0.38	1.62
20	553	8.766	3.108	0.446	0.405	0.016	1.675	3.882	57.72	*-32.80*	1.52	1.03	1.98
16	556.4	8.209	3.037	0.556	0.135	0.015	1.769	3.366	59.52	*-32.60*	1.75	3.06	2.19
14	558	8.608	3.449	0.528	0.433	0.014	1.913	3.875	58.50	*-33.40*	2.03	1.53	2.20

SCYW-79-1A (Stuart Creek Yara Wurta) Adelaide Superbasin, 30°07'35"S, 137°09'19"E.

Fe-speciation data from italicized samples originally published in Canfield et al. (ref. 37)

Additional $\delta^{34}S_{py}$ data for SCYW-79-1A published in Gorjan et al. (ref. 15)

Additional $\delta^{13}C_{org}$ data for SCYW-79-1A published in McKirdy et al. (138)

ID	Depth (m)	Al (wt%)	Fe_T (wt%)	Fe_{carb} (wt%)	Fe_{ox} (wt%)	Fe_{mag} (wt%)	Fe_{py} (wt%)	TOC (wt%)	$\delta^{34}S_{py}$ (‰)	$\delta^{13}C_{org}$ (‰)	Mo (ppm)	Re (ppb)	U (ppm)
Tapley Hill Formation													
1108619	1189.8	6.090	3.191	0.411	0.078	0.099	0.139	0.134	56.92	ND	0.96	9.03	3.05
20	1190	5.808	3.096	0.180	0.450	0.060	0.300	0.158	ND	ND	1.28	6.52	3.00
1108620	1196.1	5.966	3.272	0.389	0.073	0.093	0.302	0.135	32.37	ND	1.87	17.05	3.31
21	1200	4.361	2.820	0.300	0.660	0.040	0.310	0.126	ND	ND	2.45	1.06	2.22
1108621	1202.9	7.842	3.641	0.312	0.089	0.113	0.317	0.229	34.03	ND	1.62	1.53	4.46
1108622	1208.1	6.104	3.914	0.428	0.086	0.111	0.245	0.148	45.76	ND	1.07	1.16	3.52
22	1210	5.020	3.919	0.410	0.930	0.080	0.640	0.234	ND	ND	2.02	1.41	3.07
1108623	1216.85	6.878	3.965	0.436	0.109	0.146	0.081	0.237	41.02	ND	0.86	1.26	4.07
23	1220.2	5.186	3.633	0.380	0.720	0.080	0.200	0.186	ND	ND	1.31	1.38	2.66
1108624	1223.8	6.253	4.040	0.633	0.107	0.151	0.141	0.185	34.87	ND	1.60	1.55	3.63
1108625	1226.9	6.645	4.322	0.486	0.124	0.169	0.282	0.208	16.71	ND	0.85	1.23	4.10
1108626	1238.5	6.439	4.234	0.634	0.130	0.175	0.059	0.229	32.52	ND	0.82	1.47	3.90
24	1230.4	6.702	4.183	0.330	0.560	0.180	0.320	0.287	ND	ND	2.44	1.80	3.76
25	1240	6.862	4.283	0.290	0.510	0.150	0.540	0.289	ND	ND	1.84	1.56	3.72
1108627	1244.15	7.137	3.930	0.453	0.117	0.168	0.088	0.308	35.79	ND	1.41	3.19	4.75
1108628	1245.9	4.871	3.116	0.474	0.123	0.177	0.288	0.276	27.40	ND	0.75	1.29	2.74
26	1250	6.077	3.971	0.360	0.760	0.170	0.180	0.314	ND	ND	1.42	1.42	3.33
1108629	1257.8	6.473	4.419	0.590	0.120	0.176	0.333	0.270	35.01	ND	1.29	1.93	3.60
27	1260	5.702	4.107	0.320	0.750	0.150	0.760	0.321	ND	ND	5.13	1.59	3.28
1108630	1264.4	6.840	4.405	0.527	0.125	0.187	0.092	0.269	38.64	ND	1.18	1.42	3.93
1108631	1269.1	6.014	4.150	0.731	0.123	0.179	0.225	0.207	35.86	ND	1.07	1.29	3.35
28	1270	6.672	4.125	0.300	0.600	0.230	0.350	0.325	ND	ND	1.88	1.52	3.66
1108632	1276.9	7.579	4.678	0.652	0.144	0.222	0.466	0.300	34.12	ND	1.08	1.63	4.39
29	1280.3	7.103	4.487	0.270	0.520	0.240	0.670	0.368	ND	ND	1.54	1.84	3.88
1108633	1287.2	7.764	5.264	0.605	0.110	0.166	0.405	0.255	37.59	ND	1.45	1.55	4.30
30	1290.3	6.258	4.011	0.310	0.630	0.210	0.410	0.348	ND	ND	1.63	2.05	3.63
1108634	1294.1	4.805	4.259	1.493	0.111	0.124	0.413	0.229	60.06	ND	1.20	1.30	2.65
1108635	1297.5	7.134	4.233	0.518	0.129	0.185	0.535	0.367	34.99	ND	1.32	1.55	4.26

31	1300	5.085	3.748	0.300	0.830	0.130	0.740	0.337	ND	ND	1.84	1.47	2.79
1108636	1307.75	6.658	4.108	0.597	0.110	0.149	0.570	0.300	33.83	ND	1.38	1.57	3.80
32	1310.2	6.324	4.245	0.310	0.570	0.170	0.710	0.445	ND	ND	1.82	1.96	3.54
1108637	1312.7	7.186	4.346	0.506	0.111	0.149	0.853	0.502	36.14	ND	1.76	2.27	4.23
1108638	1316.8	6.911	4.370	0.509	0.102	0.146	0.138	0.424	39.75	ND	1.53	2.54	4.15
33	1320.2	6.494	4.145	0.240	0.440	0.180	0.640	0.353	ND	ND	1.85	1.79	3.72
1108639	1328.3	7.180	4.793	0.434	0.080	0.090	1.241	0.549	27.29	ND	2.28	2.23	3.33
1108640	1332.7	6.974	4.378	0.439	0.125	0.132	1.018	0.444	31.25	ND	1.70	2.23	4.09
1108641	1341.7	7.073	4.312	0.395	0.079	0.095	1.354	0.440	28.21	ND	2.03	2.90	4.11
1108642	1349.3	7.052	4.216	0.474	0.103	0.143	0.526	0.430	39.08	ND	1.62	1.79	4.15
1108643	1356.7	6.703	4.378	0.574	0.084	0.100	1.539	0.481	24.22	ND	2.08	2.35	3.72
1108644	1358.5	7.533	4.725	0.456	0.096	0.116	1.677	0.681	26.37	ND	3.94	3.28	3.76
1108645	1359.1	7.447	4.711	0.524	0.100	0.118	1.797	0.864	25.65	ND	2.38	3.04	3.96
1108646	1361	7.349	5.052	0.501	0.090	0.103	2.162	0.672	28.52	ND	2.98	4.27	4.11
1108647	1363	7.467	4.506	0.449	0.086	0.101	1.547	0.920	22.80	ND	3.56	5.01	4.01
1108648	1364.1	7.422	5.477	0.650	0.096	0.107	2.478	0.873	23.46	ND	2.80	6.10	4.10
1108649	1364.5	7.151	5.121	0.567	0.089	0.107	1.818	0.776	26.42	ND	2.41	6.01	3.44
1108650	1366.75	7.464	4.774	0.503	0.078	0.087	1.986	0.900	24.65	ND	2.79	7.12	3.70
1108651	1367.5	7.876	5.030	0.584	0.115	0.088	2.140	0.754	27.55	ND	2.58	6.47	3.74
1108652	1369.2	8.288	4.418	0.607	0.138	0.061	2.245	1.111	34.11	ND	4.29	7.56	4.09
1108653	1371.1	7.310	3.646	0.524	0.104	0.044	1.662	1.113	24.23	ND	6.37	3.63	3.55
1108654	1371.4	7.199	4.568	0.484	0.069	0.062	2.135	0.494	21.03	ND	3.53	7.07	3.27
1108655	1371.8	5.478	4.306	0.568	0.161	0.061	2.098	0.249	21.59	ND	4.01	1.73	2.32
Appila Tillite													
39	1380.2	5.825	1.950	0.160	0.480	0.030	0.130	ND	ND	ND	ND	ND	ND
1108657	1382.4	ND	1.810	0.170	0.540	0.040	0.190	ND	ND	ND	ND	ND	ND
40	1389.9	6.332	1.650	0.210	0.550	0.020	0.100	ND	ND	ND	ND	ND	ND
1108658	1391.8	ND	0.950	0.160	0.550	0.010	0.030	ND	ND	ND	ND	ND	ND
1108659	1406	ND	1.890	0.210	0.600	0.030	0.100	ND	ND	ND	ND	ND	ND
1108660	1412.8	ND	2.050	0.200	0.560	0.050	0.070	ND	ND	ND	ND	ND	ND
1108661	1418.5	ND	2.200	0.300	0.630	0.050	0.100	ND	ND	ND	ND	ND	ND
1108662	1428.3	ND	2.200	0.270	0.410	0.060	0.010	ND	ND	ND	ND	ND	ND
1108663	1434	ND	1.810	0.240	0.330	0.030	0.020	ND	ND	ND	ND	ND	ND

SR/17-2 (Adelaide Superbasin, ~65km NW of SCYW-79-1A)

Fe-speciation data originally published in Canfield et al. (ref. 37)

ID	Depth (m)	Al (wt%)	Fe_T (wt%)	Fe_{carb} (wt%)	Fe_{ox} (wt%)	Fe_{mag} (wt%)	Fe_{py} (wt%)	TOC (wt%)	$\delta^{34}S_{py}$ (‰)	$\delta^{13}C_{org}$ (‰)	Mo (ppm)	Re (ppb)	U (ppm)
Tapley Hill Formation													
87	495	5.488	3.073	0.190	0.420	0.070	0.010	0.084	ND	ND	1.00	1.51	3.62
88	505	4.564	3.023	0.260	0.900	0.070	0.090	0.080	ND	ND	1.40	2.50	2.26
89	515.2	4.881	3.192	0.120	0.650	0.100	0.100	0.116	ND	ND	1.21	1.24	2.44
90	525.1	4.304	2.958	0.120	0.350	0.120	0.730	0.113	ND	ND	3.35	3.42	2.99
91	535.2	4.841	3.196	0.250	0.590	0.110	0.070	0.098	ND	ND	1.11	0.91	2.51
92	545.6	6.162	3.730	0.220	0.520	0.140	0.380	0.169	ND	ND	2.91	1.14	3.45
93	555.1	4.633	2.904	0.210	0.390	0.130	0.410	0.121	ND	ND	1.99	81.71	2.92
94	565.3	4.460	3.178	0.340	0.680	0.140	0.280	0.100	ND	ND	1.89	1.12	2.55
95	575	ND	2.660	0.500	0.330	0.100	0.110	ND	ND	ND	ND	ND	ND
96	585.2	5.066	3.369	0.310	0.540	0.130	0.020	0.090	ND	ND	0.85	0.70	2.18
97	595.2	ND	3.480	0.540	0.310	0.130	0.020	ND	ND	ND	ND	ND	ND
98	604.9	ND	3.680	0.490	0.330	0.090	0.010	ND	ND	ND	ND	ND	ND
99	615	5.516	3.962	0.280	0.190	0.040	0.070	ND	ND	ND	1.14	0.80	2.26
100	625.4	1.076	1.111	0.360	0.560	0.150	0.010	0.079	ND	ND	0.42	0.62	0.71
101	635.2	ND	2.440	0.520	0.410	0.100	0.150	ND	ND	ND	ND	ND	ND
102	645.8	4.712	3.620	0.270	0.580	0.180	0.140	0.124	ND	ND	1.11	0.69	2.69
103	655.1	ND	3.650	0.490	0.260	0.170	0.100	ND	ND	ND	ND	ND	ND
104	666	ND	1.640	0.720	0.480	0.020	0.010	ND	ND	ND	ND	ND	ND
105	675	5.627	3.537	0.280	0.750	0.190	0.080	0.182	ND	ND	1.50	0.95	2.75
106	685.2	5.749	3.535	0.320	0.540	0.280	0.080	0.242	ND	ND	1.17	1.44	2.98
107	695.3	6.018	3.934	0.290	0.670	0.280	0.060	0.233	ND	ND	1.66	1.25	3.18
108	705.7	5.869	3.725	0.350	0.680	0.240	0.080	0.274	ND	ND	1.34	1.32	3.24
109	715.2	6.206	3.971	0.280	0.790	0.260	0.050	0.206	ND	ND	1.47	1.60	3.45
110	725.4	6.154	3.975	0.290	0.640	0.300	0.060	0.219	ND	ND	1.65	1.70	3.29
111	735.3	6.005	3.976	0.340	0.630	0.280	0.200	ND	ND	ND	ND	ND	ND

112	744.9	5.853	3.541	0.290	0.540	0.240	0.040	0.185	ND	ND	1.30	1.50	3.07
113	755.6	6.040	3.586	0.300	0.500	0.240	0.170	0.333	ND	ND	4.24	2.08	3.35
114	765.6	5.452	3.863	0.390	0.850	0.210	0.430	0.235	ND	ND	2.32	1.39	2.96
115	775.6	ND	4.050	0.720	0.380	0.160	0.080	ND	ND	ND	ND	ND	ND
116	785.7	ND	3.810	1.050	0.400	0.240	0.120	ND	ND	ND	ND	ND	ND
117	796.5	ND	4.200	0.730	0.260	0.230	0.700	ND	ND	ND	ND	ND	ND
118	805.4	ND	4.130	0.740	0.220	0.210	0.730	ND	ND	ND	ND	ND	ND
119	815.3	ND	3.530	0.590	0.250	0.160	0.770	ND	ND	ND	ND	ND	ND
120	825.3	ND	4.190	0.540	0.250	0.120	1.350	ND	ND	ND	ND	ND	ND
121	837	ND	4.250	0.510	0.190	0.140	1.390	ND	ND	ND	ND	ND	ND
122	845.6	ND	4.650	0.670	0.310	0.200	1.070	ND	ND	ND	ND	ND	ND
123	855.7	ND	4.270	0.420	0.190	0.140	1.380	ND	ND	ND	ND	ND	ND
124	865.5	ND	3.900	0.510	0.210	0.110	1.120	ND	ND	ND	ND	ND	ND
125	874.9	ND	3.930	0.860	0.400	0.090	1.060	ND	ND	ND	ND	ND	ND
126	885	ND	3.700	1.000	0.440	0.120	0.880	ND	ND	ND	ND	ND	ND
127	895.4	ND	4.700	0.630	0.240	0.130	1.710	ND	ND	ND	ND	ND	ND
128	905.5	ND	3.820	1.300	0.520	0.080	1.140	ND	ND	ND	ND	ND	ND
129	915.4	ND	4.030	0.780	0.240	0.000	0.660	ND	ND	ND	ND	ND	ND

BR05-DD01 (Amadeus Basin)

ID	Depth (m)	Al (wt%)	Fe_T (wt%)	Fe_{carb} (wt%)	Fe_{ox} (wt%)	Fe_{mag} (wt%)	Fe_{py} (wt%)	TOC (wt%)	$\delta^{34}S_{py}$ (‰)	$\delta^{13}C_{org}$ (‰)	Mo (ppm)	Re (ppb)	U (ppm)
Aralka Formation													
LvM024	54.9	4.689	3.049	0.050	1.237	0.040	0.000	0.092	ND	-27.5	1.71	ND	3.60
LvM025	80.3	4.949	4.364	0.044	1.819	0.076	0.759	0.105	ND	-27.6	2.58	3.62	4.46
LvM026	100.1	5.462	3.813	0.047	1.381	0.062	0.009	0.136	ND	-27.9	2.40	ND	3.76
LvM027	121.6	5.280	3.283	0.076	1.038	0.064	0.000	ND	ND	-27.9	2.63	ND	3.62
LvM028	138.9	4.941	3.751	0.106	1.368	0.078	0.000	0.139	ND	-28	2.50	3.44	3.63
LvM029	160	2.922	2.019	0.526	0.091	0.062	0.009	ND	ND	-28.8	0.47	5.84	1.87
LvM030	163.7	4.318	2.581	0.360	0.130	0.099	0.106	ND	45.2	-28.8	0.52	2.99	1.93
LvM031	177	3.293	2.673	0.566	0.168	0.104	0.563	0.067	42.9	-28.9	1.90	3.42	1.78
LvM032	179.8	1.485	4.485	0.354	0.146	0.150	0.340	0.086	43.1	-28.7	0.56	ND	0.96
LvM033	216.9	6.010	5.392	0.438	0.198	0.521	0.366	ND	51.1	-28.9	1.46	3.75	3.08
LvM034	240	ND	2.830	0.960	0.087	0.100	0.286	ND	41.6	-28.9	ND	ND	ND
LvM035	260.1	5.788	4.606	ND	ND	ND	ND	ND	ND	-28.5	4.95	4.04	3.26
LvM036	266.8	6.510	5.237	0.807	0.168	0.521	0.310	ND	44.2	-28.8	2.95	3.83	3.32
LvM037	280.4	4.770	4.328	0.728	0.174	0.456	0.498	0.176	36.0	-29.4	1.84	2.83	2.33
LvM038	309.4	5.548	4.772	0.843	0.132	0.334	0.529	0.178	20.8	-29.2	1.72	4.32	2.75
LvM039	323.3	4.442	4.814	1.562	0.210	0.387	0.592	0.180	16.3	-29.8	2.11	3.19	2.45
LvM040	343.6	7.107	5.404	0.448	0.234	0.626	0.124	ND	8.1	-28.3	3.12	6.42	3.86
LvM041	353.1	6.202	5.165	0.567	0.218	0.585	0.551	ND	31.6	-29.2	2.05	4.32	3.45
LvM042	362.8	6.292	5.378	0.711	0.226	0.770	0.018	ND	ND	-28.5	0.45	3.00	2.79
LvM043	371.6	5.279	4.748	0.652	0.177	0.547	0.147	ND	35	-29.3	0.78	4.13	2.25
LvM044	385.3	5.879	5.113	0.436	0.262	0.335	0.883	0.271	6.1	-31.8	2.72	4.74	2.80
LvM045	393.4	6.550	5.492	0.561	0.378	0.558	0.092	ND	1.8	-28.2	2.16	5.11	3.54
LvM046	396.2	6.812	5.441	0.536	0.241	0.657	0.214	ND	5.4	-28.5	7.56	4.75	3.55
LvM047	410.2	5.827	5.168	0.634	0.194	0.588	0.193	ND	35.7	-28.8	0.90	3.88	2.50
LvM048	414.4	6.177	5.020	0.548	0.198	0.512	0.492	ND	19.7	-29.7	1.36	4.26	2.92
LvM049	420.9	5.877	4.517	0.584	0.160	0.502	0.278	ND	10.8	-29.3	0.78	3.67	2.62
LvM050	431.1	6.631	6.158	0.571	0.741	0.750	0.022	ND	ND	-28.4	0.56	2.94	2.88
LvM051	440.7	6.156	5.090	0.504	0.156	0.607	0.126	ND	11.1	-28.4	2.20	3.90	3.00
LvM052	441.4	6.680	5.320	0.507	0.205	0.640	0.073	ND	10.7	-28.8	0.64	3.71	3.01
LvM053	442.8	5.232	4.935	0.804	0.179	0.415	0.731	0.209	34.8	-31	1.27	4.24	2.47
LvM054	444	5.687	4.463	0.591	0.191	0.538	0.045	ND	18.3	-28.9	0.39	ND	2.27
LvM055	445.2	6.237	4.974	0.531	0.219	0.443	0.648	ND	28.5	-30.7	1.38	4.46	2.77
LvM056	446.4	6.041	5.298	0.619	0.258	0.489	0.965	0.283	25.5	-30.8	1.29	3.98	2.87
LvM057	447	4.636	2.983	0.373	0.140	0.254	0.268	ND	33.3	-30.5	0.97	ND	1.77
LvM058	448	5.895	5.457	0.392	0.403	0.565	1.045	0.381	19.6	-32.1	1.56	6.51	3.26
LvM059	448.9	6.215	5.051	0.401	0.310	0.370	1.245	0.338	20.6	-31.8	4.18	4.44	2.94
LvM060	449.4	5.013	3.704	0.508	0.083	0.186	0.759	0.123	39.3	-29.6	2.16	ND	1.96
LvM061	451.4	6.591	5.961	0.447	0.205	0.943	0.100	ND	8	-29.6	1.17	3.94	3.42
LvM062	452.2	6.949	4.174	0.301	0.148	0.446	0.143	ND	22.7	-29.3	1.01	3.21	3.33
LvM063	453	6.371	4.485	0.378	0.164	0.505	0.051	ND	26.7	-29.4	0.72	2.66	2.52
LvM064	454	6.111	4.762	0.633	0.112	0.287	0.533	ND	43.3	-29.6	0.92	3.94	2.69

LvM065	455	5.857	5.299	0.623	0.226	0.528	0.951	0.282	32.4	-31.1	1.38	3.42	2.91
LvM066	456.3	5.883	5.074	0.608	0.224	0.504	0.829	0.262	24.4	-31	1.59	4.41	2.85
LvM067	457.2	6.245	5.135	0.523	0.151	0.611	0.510	ND	23.1	-29.8	1.25	4.06	2.90
LvM068	458.2	5.789	5.235	0.673	0.207	0.515	0.834	0.250	19.4	-31.5	1.23	4.98	2.95
LvM069	459.2	5.427	5.062	0.872	0.237	0.444	1.059	0.282	18.8	-32.1	2.60	4.57	2.88
LvM070	460.3	5.114	5.031	0.577	0.270	0.457	1.192	0.307	21.9	-32.8	2.24	5.83	2.66
LvM071	461	5.019	4.793	0.293	0.098	0.427	0.112	0.129	-13.1	-28.5	0.30	2.75	1.86
LvM072	461.8	6.388	5.971	0.489	0.174	0.820	0.092	ND	7.9	-29	1.34	4.37	3.32
LvM073	463.4	6.543	5.589	0.447	0.143	0.742	0.016	ND	ND	-28.6	ND	ND	2.76
LvM074	464.4	6.650	5.476	0.501	0.186	0.798	0.309	ND	15.5	-29.9	1.91	4.00	3.27
LvM075	466.1	6.986	5.434	0.475	0.160	0.841	0.101	ND	13.5	-29.5	0.64	3.71	2.90
LvM076	467	4.835	5.189	1.300	0.224	0.364	1.122	0.314	29	-32.6	1.81	4.23	2.63
LvM077	468.1	6.344	4.703	0.475	0.166	0.482	0.610	ND	52.7	-30.7	1.10	4.17	2.72
LvM078	469.1	4.953	4.846	1.067	0.152	0.346	1.225	0.377	17.5	-33.5	1.77	6.05	2.72
LvM079	470.1	2.753	5.429	2.352	0.156	0.317	0.313	0.086	16.8	-31.1	0.57	ND	1.28
LvM080	471	5.342	4.712	0.504	0.205	0.357	1.050	0.307	28.9	-35.4	1.15	6.12	2.81
LvM081	472.2	5.937	4.226	0.381	0.214	0.287	1.219	0.307	15	-36.4	1.47	4.97	2.94
LvM082	473.1	ND	4.578	1.927	0.091	0.105	0.211	ND	20	-31.7	ND	ND	ND
LvM083	474.1	6.038	4.796	0.456	0.176	0.326	1.430	0.514	22.1	-33.5	1.48	6.27	3.01
LvM084	475	5.603	4.402	0.460	0.219	0.240	1.389	0.397	21.2	-34.4	2.62	5.34	2.69
LvM085	476	5.442	4.312	0.502	0.223	0.243	1.364	0.471	19.3	-33.6	3.09	6.81	2.89
LvM086	476.7	5.367	4.395	0.497	0.235	0.218	1.344	0.408	20.4	-34.1	2.50	5.84	2.73
LvM087	477.6	5.142	4.263	0.458	0.191	0.160	1.569	0.452	21.7	-34	2.80	6.83	2.65
LvM088	478	5.407	4.186	0.485	0.095	0.164	1.591	0.420	22.1	-33.8	3.20	7.14	2.76
LvM089	479.2	5.239	4.286	0.395	0.207	0.145	1.522	0.378	21.7	-34.8	2.30	6.16	2.58
LvM090	480	5.302	4.197	0.489	0.193	0.183	1.553	0.433	21.9	-34.5	3.02	7.48	2.72
LvM091	480.7	5.213	4.258	0.526	0.159	0.167	1.533	0.404	20	-34.6	3.72	8.73	2.62
LvM092	481.2	5.561	4.203	0.553	0.170	0.183	1.483	0.348	22.7	-35.2	4.14	9.12	2.72
LvM093	481.6	4.957	4.100	0.740	0.131	0.163	1.401	0.293	23.3	-35.6	3.65	6.57	2.40
LvM094	481.9	2.852	2.861	0.727	0.086	0.134	0.953	0.210	29.4	-36.5	2.19	3.95	1.20
LvM095	482.3	5.744	4.683	0.567	0.155	0.264	1.639	0.712	18	-37.5	7.13	11.08	2.97
LvM096	482.7	5.903	3.670	0.359	0.168	0.232	1.138	0.404	10.9	-39.8	4.58	5.89	3.65
LvM097	483.3	6.988	4.737	0.412	0.211	0.466	0.247	ND	20.5	-33.5	1.48	5.19	2.73
LvM098	483.6	6.727	5.012	0.362	0.220	0.615	0.145	ND	38.1	ND	0.73	6.53	2.67
LvM099	484.1	6.445	7.770	0.390	0.261	0.507	3.290	0.110	5.4	-32.9	1.98	20.65	4.13
LvM100	484.6	6.535	5.028	0.378	0.337	0.621	0.086	ND	3.9	-30.5	0.74	6.90	3.28
Areyonga Diamictite													
LvM101	485.1	6.679	5.747	0.375	0.790	0.772	0.015	ND	-0.3	-29.9	0.77	4.55	3.07
LvM102	485.7	6.726	6.015	0.341	0.918	0.749	0.036	ND	-0.2	-29.7	0.83	3.74	2.97
LvM103	486.1	7.043	5.531	0.266	0.786	0.617	0.031	ND	0.2	-29.1	0.82	4.06	2.66
LvM104	487.3	6.665	6.035	0.245	1.044	0.552	0.032	ND	-0.9	-29.1	0.86	3.95	2.54
LvM105	488.1	6.690	6.078	0.249	1.149	0.575	0.010	ND	ND	-28.9	0.82	3.52	2.34
LvM106	488.6	6.919	5.931	0.255	1.018	0.540	0.029	ND	-4.1	-28.9	0.86	4.47	2.45
LvM107	488.9	7.264	5.176	0.266	0.540	0.486	0.033	ND	-3.3	-28.9	0.84	5.03	2.88
LvM108	489	5.744	3.106	0.297	0.112	0.224	0.322	ND	32	-29.2	1.62	14.12	6.25
LvM109	490.1	5.894	4.618	0.353	0.646	0.329	0.000	ND	ND	-28.9	0.55	3.50	2.40
LvM110	491.1	5.950	4.673	0.324	0.827	0.345	0.100	ND	4.7	-28.9	0.64	3.72	2.35
LvM111	492	5.739	4.111	0.309	0.582	0.296	0.010	ND	ND	-28.8	0.53	4.13	2.42
LvM112	492.8	5.727	4.233	0.311	0.651	0.360	0.011	ND	ND	-28.7	0.55	3.81	2.20
LvM113	495	4.354	2.720	0.290	0.324	0.170	0.000	ND	ND	-27.8	1.69	ND	1.84
LvM114	498.6	4.115	2.375	0.260	0.297	0.123	0.011	ND	ND	-25.3	0.58	ND	1.98
LvM115	504.4	4.190	2.404	0.225	0.390	0.094	0.011	ND	ND	-27.1	0.79	ND	1.93
LvM116	509.3	4.155	2.540	0.200	0.490	0.110	0.022	ND	ND	-27.4	0.51	ND	1.90
LvM117	514.3	5.547	3.594	0.172	0.643	0.208	0.011	ND	ND	-28	0.66	3.27	2.21
LvM118	521.1	4.699	2.799	0.382	0.423	0.104	0.000	ND	ND	-28.3	0.34	ND	1.96
LvM119	526	4.477	2.748	0.380	0.414	0.104	0.015	ND	ND	-28.2	0.38	ND	2.07
LvM120	531.2	4.438	2.542	0.273	0.252	0.073	0.102	ND	-6.2	-28.4	0.56	5.60	2.15
LvM121	540.7	4.777	1.887	0.074	0.052	0.018	0.013	ND	ND	ND	ND	ND	2.01

Wallara-1 (Amadeus Basin)													
ID	Depth (m)	Al (wt%)	Fe_T (wt%)	Fe_{carb} (wt%)	Fe_{ox} (wt%)	Fe_{mag} (wt%)	Fe_{py} (wt%)	TOC (wt%)	$\delta^{34}S_{py}$ (‰)	$\delta^{13}C_{org}$ (‰)	Mo (ppm)	Re (ppb)	U (ppm)
Aralka Formation													
LvM122	1289.7	1.765	1.677	0.617	0.045	0.041	0.447	0.259	25.35	-32.1	1.44	ND	1.25
LvM123	1291.2	6.219	4.423	0.453	0.089	0.121	1.620	0.699	24.86	-32.2	3.56	5.17	3.68
LvM124	1293.4	6.056	5.060	0.500	0.103	0.133	1.757	0.747	24.91	-32.5	4.12	5.47	3.67
LvM125	1295.2	1.883	2.526	1.109	0.062	0.054	0.705	0.242	26.43	-33.1	2.10	3.05	1.32
LvM126	1297.4	6.139	4.599	0.425	0.114	0.115	1.422	0.815	23.69	-32.5	4.38	6.47	3.85
LvM127	1298.4	2.151	3.052	1.080	0.069	0.059	1.043	0.352	25.14	-33.2	4.74	3.40	1.27
LvM128	1299.1	4.186	3.725	1.294	0.091	0.086	1.069	0.553	22.43	-33.4	3.52	5.58	2.48
LvM129	1301.1	5.692	4.226	0.589	0.106	0.102	1.882	0.785	24.24	-33.5	9.93	8.33	3.25
LvM130	1303.3	5.067	4.257	0.805	0.104	0.093	1.624	0.641	ND	-34.6	5.79	8.43	2.91
LvM131	1304.2	6.356	4.836	0.467	0.125	0.095	2.162	0.803	24.36	-35.3	9.03	12.59	3.10
LvM132	1304.8	6.755	4.617	0.428	0.101	0.083	2.069	0.926	26.47	-35.5	14.39	11.44	3.15
LvM133	1305.3	4.791	3.560	0.450	0.070	0.057	2.271	0.692	17.82	-37	12.64	5.23	3.28
LvM134	1305.8	4.977	2.745	0.492	0.068	0.066	1.071	0.123	46.22	-33.1	0.49	ND	2.41
LvM135	1306.1	4.918	2.545	0.466	0.065	0.063	0.926	0.112	54.19	-32.8	0.48	ND	2.67
LvM136	1306.3	4.505	2.651	0.629	0.069	0.056	0.837	0.131	29.04	-34.6	0.78	3.09	2.97
Areyonga Diamictite													
LvM137	1306.8	1.930	6.492	0.558	0.053	0.030	4.961	ND	23.06	-22.9	12.57	ND	1.62
LvM138	1310	4.192	2.397	0.424	0.073	0.072	0.686	ND	40.52	-29.4	0.45	ND	1.46
LvM139	1318.8	3.384	1.676	0.550	0.055	0.051	0.103	ND	ND<br>10.38	-28.2	0.37	2.80	1.51

Table S5. Analytical Data 2: Total P concentrations (P_{Tot}), and P speciation. Depth (m) indicates sample position relative to drill core datum. BD = below detection; ND = not determined.

ZK102 core								
ID	Depth (m)	P_{Tot} (ppm)	P_{Fe} (ppm)	P_{auth} (ppm)	P_{det} (ppm)	P_{org} (ppm)	P_{SUM} (ppm)	P_{reac} (ppm)
Tiesi'ao Formation								
DTP-1	1322	699	12	102	463	4	581	118
DTP-2	1321	854	13	93	599	9	715	116
DTP-3	1320.5	971	18	17	827	12	874	47
DTP-4	1320.3	728	14	21	620	11	667	47
Datangpo Formation								
DTP-5	1319.6	ND	ND	ND	ND	ND	ND	ND
DTP-6	1319	ND	ND	ND	ND	ND	ND	ND
DTP-7	1318	ND	ND	ND	ND	ND	ND	ND
DTP-8	1316	ND	ND	ND	ND	ND	ND	ND
DTP-10	1312	692	11	375	236	11	633	398
DTP-11	1310	ND	ND	ND	ND	ND	ND	ND
DTP-12	1308.8	416	9	195	148	10	362	215
DTP-13	1307	359	7	165	143	10	324	181
DTP-14	1305	387	10	94	233	10	348	114
DTP-15	1303	421	6	264	85	8	364	278
DTP-16	1301	412	8	187	185	7	388	203
DTP-17	1300	408	6	269	89	11	375	286
DTP-18	1298.4	356	9	116	194	10	328	134
DTP-19	1297	403	9	144	201	10	365	164
DTP-20	1295	348	8	73	215	9	305	90
DTP-21	1292	379	9	135	196	8	347	152
DTP-22	1290	469	13	38	365	11	427	62
DTP-23	1287	344	10	57	246	9	322	76
DTP-24	1285	303	8	100	154	10	272	118
DTP-25	1283	320	9	5	244	18	276	32
DTP-26	1282	302	8	6	234	12	261	27
DTP-27	1279	496	11	165	261	10	447	186
DTP-28	1276	316	7	113	151	11	282	131

DTP-29	1275	306	9	27	228	9	272	44
DTP-30	1272	293	9	45	194	11	258	64
DTP-31	1270.5	370	12	91	208	11	321	113
DTP-32	1270	497	11	105	323	10	448	125
DTP-33	1267	297	8	93	139	11	251	112
DTP-34	1264	362	10	82	211	11	314	103
DTP-35	1262	341	10	55	220	10	294	74
DTP-36	1260	269	7	106	108	8	229	121
DTP-37	1258	333	8	104	150	11	274	124
DTP-38	1256	324	11	76	172	4	263	91
DTP-39	1253	331	6	227	39	2	275	235
DTP-40	1251	295	8	101	127	3	239	112
DTP-41	1249	356	8	196	100	4	308	209
DTP-42	1247	335	5	219	32	3	260	228
DTP-43	1244	273	7	134	87	4	232	144
DTP-44	1243.5	323	9	167	98	6	281	183
DTP-45	1239	307	9	96	140	4	248	109
DTP-46	1236	310	10	73	191	3	276	85
DTP-47	1232	5075	99	234	3864	6	4203	339
DTP-48	1231	376	5	110	181	3	300	119
DTP-49	1228	645	13	130	403	9	555	152
DTP-50	1225	339	ND	ND	ND	ND	ND	ND
DTP-51	1221	337	5	137	121	6	269	148
DTP-52	1219	325	3	205	69	5	282	213
DTP-53	1216	306	6	77	185	5	273	88
DTP-54	1215.5	286	5	98	128	7	237	110
DTP-55	1212.5	304	6	63	169	8	246	77
DTP-56	1210	333	4	163	95	5	266	172
DTP-57	1208	341	4	160	111	2	277	166
DTP-58	1206	313	5	74	171	5	255	85
DTP-59	1204	259	5	35	165	7	212	48
DTP-60	1202	335	3	210	49	6	267	218
DTP-61	1198	270	3	155	42	6	206	164
DTP-62	1196	610	8	301	182	4	495	313
DTP-63	1193	440	7	73	312	4	396	84
DTP-64	1188	583	4	151	151	6	312	161
DTP-65	1182	280	4	133	118	8	262	144
DTP-66	1180	279	4	138	99	6	247	148
DTP-67	1177	390	8	197	165	5	375	210
DTP-68	1171	262	3	159	82	5	249	167
DTP-69	1167	228	5	84	122	6	217	95
DTP-70	1162	396	4	287	48	6	345	297
DTP-71	1159	213	2	165	27	11	205	178
DTP-72	1154	273	3	183	46	5	237	191
DTP-73	1151	292	4	145	108	5	263	154
DTP-74	1146	326	6	213	65	5	289	224
DTP-75	1142	256	5	160	62	4	230	168
DTP-76	1137	318	3	186	67	5	260	194
DTP-77	1130	357	6	85	225	4	321	96
DTP-78	1124	253	7	47	171	4	229	57
DTP-79	1121	274	5	74	144	4	227	83
DTP-80	1117	258	7	21	184	4	217	32
DTP-81	1113	329	7	51	195	6	259	64
DTP-82	1109	347	7	76	166	5	255	88
DTP-83	1105	266	7	96	147	5	256	108
DTP-84	1104	308	6	58	177	6	247	70
DTP-85	1101	395	5	248	95	4	352	256
DTP-86	1099	300	6	91	163	5	265	102
DTP-87	1096	1025	14	226	629	6	876	247
DTP-88	1089	322	6	71	191	5	273	82
DTP-89	1085	985	11	372	433	5	822	388
DTP-90	1082	325	6	122	110	5	243	133
DTP-91	1076	956	16	175	499	6	696	197

DTP-92	1072	546	10	88	384	7	488	104
DTP-93	1069	499	8	78	326	10	421	96
DTP-94	1068	1246	23	99	973	6	1100	127
DTP-95	1064	776	13	140	523	6	682	159
DTP-96	1061	766	12	181	494	6	694	200
DTP-97	1060	560	8	155	320	8	491	171
Nantuo Formation								
DTP-98	1059	466	8	123	275	9	415	141
DTP-99	1057	448	5	287	121	8	421	300
DTP-100	1055	449	4	270	133	5	413	280
DTP-101	1054	407	5	223	146	5	379	233

ZK3603 core

ID	Depth (m)	P_{Tot} (ppm)	P_{Fe} (ppm)	P_{auth} (ppm)	P_{det} (ppm)	P_{org} (ppm)	P_{SUM} (ppm)	P_{reac} (ppm)
Xiangmeng Formation								
94	482	524	7	272	191	19	490	298
92	484	663	7	367	198	15	586	388
87	490.5	133	17	58	35	13	124	88
84	493.5	164	24	59	50	10	142	93
78	500.5	335	117	114	43	13	287	244
74	502.5	352	197	63	34	14	308	274
72	504	361	154	97	45	17	312	268
70	506.5	1069	33	854	83	11	981	898
66	510.5	495	4	401	79	9	494	415
64	512	363	127	129	38	14	309	271
59	517	720	246	346	47	19	659	612
57	519	377	210	68	30	15	323	292
49	526.5	403	31	233	63	16	343	280
45	530	426	13	277	68	14	373	305
41	533.5	316	233	7	8	15	263	255
37	537.5	387	233	45	24	17	320	295
35	539	334	134	106	36	18	294	258
31	543	298	146	58	33	14	251	218
24	549.6	281	109	78	38	14	238	200
20	553	320	98	114	42	15	269	226
16	556.4	307	4	194	41	19	258	217
14	558	336	92	124	43	14	273	230

SCYW-79-1A (Stuart Creek Yara Wurta) Adelaide Superbasin, 30°07'35"S, 137°09'19"E.

ID	Depth (m)	P_{Tot} (ppm)	P_{Fe} (ppm)	P_{auth} (ppm)	P_{det} (ppm)	P_{org} (ppm)	P_{SUM} (ppm)	P_{reac} (ppm)
Tapley Hill Formation								
1108619	1189.8	979	ND	ND	ND	ND	ND	ND
20	1190	877	ND	ND	ND	ND	ND	ND
1108620	1196.1	1010	ND	ND	ND	ND	ND	ND
21	1200	868	ND	ND	ND	ND	ND	ND
1108621	1202.9	1190	ND	ND	ND	ND	ND	ND
1108622	1208.1	886	ND	ND	ND	ND	ND	ND
22	1210	723	2	221	430	7	676	246
1108623	1216.85	838	2	237	420	13	692	272
23	1220.2	734	0	41	557	8	619	62
1108624	1223.8	902	ND	ND	ND	ND	ND	ND
1108625	1226.9	873	ND	ND	ND	ND	ND	ND
1108626	1238.5	909	ND	ND	ND	ND	ND	ND
24	1230.4	869	0	83	699	11	812	113
25	1240	794	0	132	502	11	663	161
1108627	1244.15	962	2	291	479	21	818	338
1108628	1245.9	685	ND	ND	ND	ND	ND	ND
26	1250	790	0	44	660	8	730	70
1108629	1257.8	861	ND	ND	ND	ND	ND	ND
27	1260	743	0	48	600	8	672	71
1108630	1264.4	880	2	205	531	18	781	250
1108631	1269.1	901	ND	ND	ND	ND	ND	ND
28	1270	843	0	127	512	9	664	152
1108632	1276.9	968	ND	ND	ND	ND	ND	ND

29	1280.3	824	0	154	546	8	728	181
1108633	1287.2	987	ND	ND	ND	ND	ND	ND
30	1290.3	834	0	25	707	8	760	53
1108634	1294.1	777	ND	ND	ND	ND	ND	ND
1108635	1297.5	869	ND	ND	ND	ND	ND	ND
31	1300	782	0	82	630	8	733	104
1108636	1307.75	879	ND	ND	ND	ND	ND	ND
32	1310.2	844	0	36	625	8	686	61
1108637	1312.7	923	ND	ND	ND	ND	ND	ND
1108638	1316.8	883	2	156	572	18	771	199
33	1320.2	873	0	82	661	10	771	110
1108639	1328.3	1009	2	167	642	22	860	217
1108640	1332.7	924	ND	ND	ND	ND	ND	ND
1108641	1341.7	1001	2	257	538	32	850	312
1108642	1349.3	955	ND	ND	ND	ND	ND	ND
1108643	1356.7	977	2	128	642	40	836	194
1108644	1358.5	1122	2	272	617	26	944	326
1108645	1359.1	985	2	346	437	24	829	392
1108646	1361	1020	2	260	530	28	841	311
1108647	1363	1120	2	243	647	23	940	293
1108648	1364.1	965	2	126	601	23	777	176
1108649	1364.5	1032	2	181	670	19	896	226
1108650	1366.75	1157	1	337	622	20	1001	379
1108651	1367.5	1135	1	352	531	32	937	406
1108652	1369.2	1292	2	518	534	30	1107	573
1108653	1371.1	715	0	202	300	31	543	244
1108654	1371.4	889	0	143	531	27	717	186
1108655	1371.8	501	1	105	242	22	381	139

SR/17-2 (Adelaide Superbasin, ~65km NW of SCYW-79-1A)

ID	Depth (m)	P_{Tot} (ppm)	P_{Fe} (ppm)	P_{auth} (ppm)	P_{det} (ppm)	P_{org} (ppm)	P_{SUM} (ppm)	P_{reac} (ppm)
Tapley Hill Formation								
87	495	923	0	60	694	5	773	79
88	505	787	0	16	575	5	607	32
89	515.2	916	ND	ND	ND	ND	ND	ND
90	525.1	3140	4	617	1981	8	2650	669
91	535.2	936	ND	ND	ND	ND	ND	ND
92	545.6	1293	ND	ND	ND	ND	ND	ND
93	555.1	1342	2	169	918	6	1115	198
94	565.3	1168	2	93	842	6	965	123
96	585.2	992	ND	ND	ND	ND	ND	ND
99	615	952	ND	ND	ND	ND	ND	ND
100	625.4	857	ND	ND	ND	ND	ND	ND
101	635.2	ND	ND	ND	ND	ND	ND	ND
102	645.8	1141	ND	ND	ND	ND	ND	ND
105	675	1090	ND	ND	ND	ND	ND	ND
106	685.2	916	ND	ND	ND	ND	ND	ND
107	695.3	994	ND	ND	ND	ND	ND	ND
108	705.7	824	ND	ND	ND	ND	ND	ND
109	715.2	791	ND	ND	ND	ND	ND	ND
110	725.4	818	ND	ND	ND	ND	ND	ND
111	735.3	890	ND	ND	ND	ND	ND	ND
112	744.9	807	ND	ND	ND	ND	ND	ND
113	755.6	723	ND	ND	ND	ND	ND	ND
114	765.6	746	1	83	591	8	702	111

BR05-DD01 (Amadeus Basin)

ID	Depth (m)	P_{Tot} (ppm)	P_{Fe} (ppm)	P_{auth} (ppm)	P_{det} (ppm)	P_{org} (ppm)	P_{SUM} (ppm)	P_{reac} (ppm)
Aralka Formation								
LvM024	54.9	715	2	212	421	21	676	255
LvM025	80.3	807	3	291	438	27	785	347
LvM026	100.1	742	3	271	394	32	721	327
LvM027	121.6	709	ND	ND	ND	ND	ND	ND
LvM028	138.9	741	2	155	501	31	713	212

LvM029	160	565	ND	ND	ND	ND	ND	ND
LvM030	163.7	540	ND	ND	ND	ND	ND	ND
LvM031	177	449	0	45	362	9	425	63
LvM032	179.8	314	ND	ND	ND	ND	ND	ND
LvM033	216.9	734	ND	ND	ND	ND	ND	ND
LvM035	260.1	729	ND	ND	ND	ND	ND	ND
LvM036	266.8	744	ND	ND	ND	ND	ND	ND
LvM037	280.4	640	0	67	510	31	624	114
LvM038	309.4	677	0	61	520	59	659	139
LvM039	323.3	887	2	147	671	47	890	219
LvM040	343.6	813	ND	ND	ND	ND	ND	ND
LvM041	353.1	733	ND	ND	ND	ND	ND	ND
LvM042	362.8	703	ND	ND	ND	ND	ND	ND
LvM043	371.6	678	ND	ND	ND	ND	ND	ND
LvM044	385.3	739	0	87	538	64	708	170
LvM045	393.4	987	ND	ND	ND	ND	ND	ND
LvM046	396.2	983	ND	ND	ND	ND	ND	ND
LvM047	410.2	696	ND	ND	ND	ND	ND	ND
LvM048	414.4	733	ND	ND	ND	ND	ND	ND
LvM049	420.9	666	ND	ND	ND	ND	ND	ND
LvM050	431.1	719	ND	ND	ND	ND	ND	ND
LvM051	440.7	690	ND	ND	ND	ND	ND	ND
LvM052	441.4	713	ND	ND	ND	ND	ND	ND
LvM053	442.8	689	0	50	578	37	685	108
LvM054	444	712	ND	ND	ND	ND	ND	ND
LvM055	445.2	734	ND	ND	ND	ND	ND	ND
LvM056	446.4	688	0	90	495	60	665	170
LvM057	447	548	ND	ND	ND	ND	ND	ND
LvM058	448	818	0	191	530	68	809	279
LvM059	448.9	770	0	164	499	62	743	243
LvM060	449.4	588	0	63	472	27	577	105
LvM061	451.4	771	ND	ND	ND	ND	ND	ND
LvM062	452.2	597	ND	ND	ND	ND	ND	ND
LvM063	453	708	ND	ND	ND	ND	ND	ND
LvM064	454	676	ND	ND	ND	ND	ND	ND
LvM065	455	682	0	114	497	55	684	187
LvM066	456.3	703	0	108	500	57	686	186
LvM067	457.2	708	ND	ND	ND	ND	ND	ND
LvM068	458.2	718	0	88	537	65	710	173
LvM069	459.2	728	0	66	567	66	718	151
LvM070	460.3	633	0	106	492	68	682	191
LvM071	461	612	0	146	371	38	569	197
LvM072	461.8	868	ND	ND	ND	ND	ND	ND
LvM073	463.4	614	ND	ND	ND	ND	ND	ND
LvM074	464.4	926	ND	ND	ND	ND	ND	ND
LvM075	466.1	623	ND	ND	ND	ND	ND	ND
LvM076	467	684	0	39	549	46	649	100
LvM077	468.1	702	ND	ND	ND	ND	ND	ND
LvM078	469.1	725	0	41	595	58	709	115
LvM079	470.1	358	0	50	274	16	347	74
LvM080	471	645	0	89	455	62	620	165
LvM081	472.2	592	0	113	362	57	544	182
LvM083	474.1	828	0	117	610	52	798	188
LvM084	475	703	0	100	520	49	686	166
LvM085	476	739	0	97	571	34	721	150
LvM086	476.7	762	0	63	617	32	730	113
LvM087	477.6	742	0	58	611	30	718	107
LvM088	478	725	0	56	596	31	702	105
LvM089	479.2	718	0	56	580	40	695	115
LvM090	480	723	0	90	555	28	691	136
LvM091	480.7	664	0	55	540	28	639	99
LvM092	481.2	666	0	58	555	28	659	104
LvM093	481.6	631	0	36	538	22	611	74

LvM094	481.9	492	1	90	371	13	485	114
LvM095	482.3	692	0	128	508	24	680	172
LvM096	482.7	585	0	121	391	23	549	158
LvM097	483.3	842	ND	ND	ND	ND	ND	ND
LvM098	483.6	885	ND	ND	ND	ND	ND	ND
LvM099	484.1	815	1	329	427	31	814	387
LvM100	484.6	845	ND	ND	ND	ND	ND	ND
Areyonga Diamictite								
LvM101	485.1	847	ND	ND	ND	ND	ND	ND
LvM102	485.7	894	ND	ND	ND	ND	ND	ND
LvM103	486.1	808	ND	ND	ND	ND	ND	ND
LvM104	487.3	879	ND	ND	ND	ND	ND	ND
LvM105	488.1	827	ND	ND	ND	ND	ND	ND
LvM106	488.6	880	ND	ND	ND	ND	ND	ND
LvM107	488.9	955	ND	ND	ND	ND	ND	ND
LvM108	489	650	ND	ND	ND	ND	ND	ND
LvM109	490.1	685	ND	ND	ND	ND	ND	ND
LvM110	491.1	716	ND	ND	ND	ND	ND	ND
LvM111	492	652	ND	ND	ND	ND	ND	ND
LvM112	492.8	642	ND	ND	ND	ND	ND	ND
LvM113	495	482	ND	ND	ND	ND	ND	ND
LvM114	498.6	446	ND	ND	ND	ND	ND	ND
LvM115	504.4	383	ND	ND	ND	ND	ND	ND
LvM116	509.3	402	ND	ND	ND	ND	ND	ND
LvM117	514.3	609	ND	ND	ND	ND	ND	ND
LvM118	521.1	547	ND	ND	ND	ND	ND	ND
LvM119	526	521	ND	ND	ND	ND	ND	ND
LvM120	531.2	496	ND	ND	ND	ND	ND	ND
LvM121	540.7	376	ND	ND	ND	ND	ND	ND

Wallara-1 (Amadeus Basin)

ID	Depth (m)	P_{Tot} (ppm)	P_{Fe} (ppm)	P_{auth} (ppm)	P_{det} (ppm)	P_{org} (ppm)	P_{SUM} (ppm)	P_{reac} (ppm)
Aralka Formation								
LvM122	1289.7	761	2	133	558	10	715	157
LvM123	1291.2	821	0	53	689	27	787	97
LvM124	1293.4	796	0	63	678	25	783	106
LvM125	1295.2	559	2	75	459	13	558	99
LvM126	1297.4	849	0	79	696	32	825	129
LvM127	1298.4	617	2	82	502	17	614	111
LvM128	1299.1	608	0	35	524	18	590	66
LvM129	1301.1	809	0	41	696	23	776	81
LvM130	1303.3	662	0	18	584	21	639	55
LvM131	1304.2	800	0	76	664	31	789	125
LvM132	1304.8	819	0	110	627	25	779	152
LvM133	1305.3	540	0	32	447	14	504	57
LvM134	1305.8	660	0	34	559	13	620	61
LvM135	1306.1	685	0	40	591	14	659	68
LvM136	1306.3	599	0	28	502	18	560	58
Areyonga Diamictite								
LvM137	1306.8	208	ND	ND	ND	ND	ND	ND
LvM138	1310	445	ND	ND	ND	ND	ND	ND
LvM139	1318.8	327	ND	ND	ND	ND	ND	ND

Other Supplementary Materials for this manuscript include the following:

Table S1 [TableS1_Cryogenian_non-glacial_age_models.xlsx]

REFERENCES AND NOTES

1. P. F. Hoffman, D. S. Abbot, Y. Ashkenazy, D. I. Benn, J. J. Brocks, P. A. Cohen, G. M. Cox, J. R. Creveling, Y. Donnadieu, D. H. Erwin, I. J. Fairchild, D. Ferreira, J. C. Goodman, G. P. Halverson, M. F. Jansen, G. Le Hir, G. D. Love, F. A. Macdonald, A. C. Maloof, C. A. Partin, G. Ramstein, B. E. J. Rose, C. V. Rose, P. M. Sadler, E. Tziperman, A. Voigt, S. G. Warren, Snowball Earth climate dynamics and Cryogenian geology-geobiology. *Sci. Adv.* **3**, e1600983 (2017).

2. J. J. Brocks, A. J. M. Jarrett, E. Sirantoine, C. Hallmann, Y. Hoshino, T. Liyanage, The rise of algae in Cryogenian oceans and the emergence of animals. *Nature* **548**, 578–581 (2017).

3. N. J. Planavsky, O. J. Rouxel, A. Bekker, S. V. Lalonde, K. O. Konhauser, C. T. Reinhard, T. W. Lyons, The evolution of the marine phosphate reservoir. *Nature* **467**, 1088–1090 (2010).

4. P. F. Hoffman, G. P. Halverson, D. P. Schrag, J. A. Higgins, E. W. Domack, F. A. Macdonald, S. B. Pruss, C. L. Blattler, P. W. Crockford, E. B. Hodgin, E. J. Bellefroid, B. W. Johnson, M. S. W. Hodgskiss, K. G. Lamothe, S. J. C. LoBianco, J. F. Busch, B. J. Howes, J. W. Greenman, L. L. Nelson, Snowballs in Africa: Sectioning a long-lived Neoproterozoic carbonate platform and its bathyal foreslope (NW Namibia). *Earth Sci. Rev.* **219**, 103616 (2021).

5. G. P. Halverson, P. F. Hoffman, D. P. Schrag, A. C. Maloof, A. H. N. Rice, Toward a Neoproterozoic composite carbon-isotope record. *Bull. Geol. Soc. Am.* **117**, 1181–1207 (2005).

6. G. P. Halverson, F. Ö. Dudás, A. C. Maloof, S. A. Bowring, Evolution of the $^{87}Sr/^{86}Sr$ composition of Neoproterozoic seawater. *Palaeogeogr. Palaeoclimatol. Palaeoecol.* **256**, 103–129 (2007).

7. J. A. Giddings, M. W. Wallace, Facies-dependent $\delta^{13}C$ variation from a Cryogenian platform margin, South Australia: Evidence for stratified Neoproterozoic oceans? *Palaeogeogr. Palaeoclimatol. Palaeoecol.* **271**, 196–214 (2009).

8. U. Bold, E. F. Smith, A. D. Rooney, S. A. Bowring, R. Buchwaldt, F. O. Dudas, J. Ramezani, J. L. Crowley, D. P. Schrag, F. A. Macdonald, Neoproterozoic stratigraphy of the Zavkhan terrane of Mongolia: The backbone for Cryogenian and early Ediacaran chemostratigraphic records. *Am. J. Sci.* **316**, 1–63 (2016).

9. C. Verdel, M. Campbell, Neoproterozoic carbon isotope stratigraphy of the Amadeus Basin, central Australia. *Bull. Geol. Soc. Am.* **129**, 1280–1299 (2017).

10. F. A. Macdonald, M. D. Schmitz, J. V. Strauss, G. P. Halverson, T. M. Gibson, A. Eyster, G. Cox, P. Mamrol, J. L. Crowley, Cryogenian of Yukon. *Precambrian Res.* **319**, 114–143 (2018).

11. J. V. Strauss, F. A. Macdonald, W. C. McClelland, Pre-Mississippian stratigraphy and provenance of the North Slope subterrane of Arctic Alaska I: Platformal carbonate rocks of the northeastern Brooks Range and their significance in circum-Arctic evolution, in *Circum-Arctic Structural Events: Tectonic Evolution of the Arctic Margins and Trans-Arctic Links with Adjacent Orogens*, K. Piepjohn, J. V. Strauss, L. Reinhardt, W. C. McClelland, Eds. (The Geological Society of America, 2018), Special Paper 541.

12. P. F. Hoffman, K. G. Lamothe, Seawater-buffered diagenesis, destruction of carbon isotope excursions, and the composition of DIC in Neoproterozoic oceans. *Proc. Natl. Acad. Sci. U.S.A.* **116**, 18874–18879 (2019).

13. L. L. Nelson, A. S. C. Ahm, F. A. Macdonald, J. A. Higgins, E. F. Smith, Fingerprinting local controls on the Neoproterozoic carbon cycle with the isotopic record of Cryogenian carbonates in the Panamint Range, California. *Earth Planet. Sci. Lett.* **566**, 116956 (2021).

14. X. Peng, X. K. Zhu, F. Shi, B. Yan, F. Zhang, N. Zhao, P. Peng, J. Li, D. Wang, G. A. Shields, A deep marine organic carbon reservoir in the non-glacial Cryogenian ocean (Nanhua Basin, South China) revealed by organic carbon isotopes. *Precambrian Res.* **321**, 212–220 (2019).

15. P. Gorjan, J. J. Veevers, M. R. Walter, Neoproterozoic sulfur-isotope variation in Australia and global implications. *Precambrian Res.* **100**, 151–179 (2000).

16. A. D. Rooney, F. A. Macdonald, J. V. Strauss, F. Ö. Dudás, C. Hallmann, D. Selby, Re-Os geochronology and coupled Os-Sr isotope constraints on the Sturtian snowball Earth. *Proc. Natl. Acad. Sci. U.S.A.* **111**, 51–56 (2014).

17. A. D. Rooney, C. Yang, D. J. Condon, M. Zhu, F. A. Macdonald, U-Pb and Re-Os geochronology tracks stratigraphic condensation in the Sturtian Snowball Earth aftermath. *Geology* **48**, 625–629 (2020).

18. G. M. Cox, V. Isakson, P. F. Hoffman, T. M. Gernon, M. D. Schmitz, S. Shahin, A. S. Collins, W. Preiss, M. L. Blades, R. N. Mitchell, A. Nordsvan, South Australian U-Pb zircon (CA-ID-TIMS) age supports globally synchronous Sturtian deglaciation. *Precambrian Res.* **315**, 257–263 (2018).

19. L. L. Nelson, E. F. Smith, E. B. Hodgin, J. L. Crowley, M. D. Schmitz, F. A. Macdonald, Geochronological constraints on Neoproterozoic rifting and onset of the Marinoan glaciation from the Kingston Peak Formation in Death Valley, California (USA). *Geology* **48**, 1083–1087 (2020).

20. X. Ma, J. Wang, Z. Wang, T. J. Algeo, C. Chen, Y. Cen, Q.-Z. Yin, C. Huang, L. Xu, C. Huang, D. Chen, Geochronological constraints on Cryogenian ice ages: Zircon U-Pb ages from a shelf section in South China. *Glob. Planet. Change.* **222**, 104071 (2023).

21. A. R. Prave, D. J. Condon, K. H. Hoffmann, S. Tapster, A. E. Fallick, Duration and nature of the end-Cryogenian (Marinoan) glaciation. *Geology* **44**, 631–634 (2016).

22. G. Shields, Working towards a new stratigraphic calibration scheme for the Neoproterozoic-Cambrian. *Eclogae Geol. Helv.* **92**, 221–233 (1999).

23. A.-S. C. Ahm, C. J. Bjerrum, P. F. Hoffman, F. A. Macdonald, A. C. Maloof, C. V. Rose, J. V. Strauss, J. A. Higgins, The Ca and Mg isotope record of the Cryogenian Trezona carbon isotope excursion. *Earth Planet. Sci. Lett.* **568**, 117002 (2021).

24. G. Le Hir, Y. Donnadieu, Y. Goddéris, R. T. Pierrehumbert, G. P. Halverson, M. Macouin, A. Nédélec, G. Ramstein, The Snowball Earth aftermath: Exploring the limits of continental weathering processes. *Earth Planet. Sci. Lett.* **277**, 453–463 (2009).

25. Y. Goddéris, G. Le Hir, M. Macouin, Y. Donnadieu, L. Hubert-Théou, G. Dera, M. Aretz, F. Fluteau, Z. X. Li, G. P. Halverson, Paleogeographic forcing of the strontium isotopic cycle in the Neoproterozoic. *Gondw. Res.* **42**, 151–162 (2017).

26. K. R. Moore, T. Bosak, F. Macdonald, K. Du, S. A. Newman, D. J. G. Lahr, S. B. Pruss, Pyritized Cryogenian cyanobacterial fossils from Arctic Alaska. *Palaios.* **32**, 769–778 (2017).

27. L. A. Riedman, P. M. Sadler, Global species richness record and biostratigraphic potential of early to middle Neoproterozoic eukaryote fossils. *Precambrian Res.* **319**, 6–18 (2018).

28. P. A. Cohen, M. Vizcaíno, R. P. Anderson, Oldest fossil ciliates from the Cryogenian glacial interlude reinterpreted as possible red algal spores. *Palaeontology* **63**, 941–950 (2020).

29. G. D. Love, E. Grosjean, C. Stalvies, D. A. Fike, J. P. Grotzinger, A. S. Bradley, A. E. Kelly, M. Bhatia, W. Meredith, C. E. Snape, S. A. Bowring, D. J. Condon, R. E. Summons, Fossil steroids record the appearance of Demospongiae during the Cryogenian period. *Nature* **457**, 718–721 (2009).

30. I. Bobrovskiy, J. M. Hope, B. J. Nettersheim, J. K. Volkman, C. Hallmann, J. J. Brocks, Algal origin of sponge sterane biomarkers negates the oldest evidence for animals in the rock record. *Nat. Ecol. Evol.* **5**, 165–168 (2020).

31. G. Burzynski, T. A. Dececchi, G. M. Narbonne, R. W. Dalrymple, Cryogenian *Aspidella* from northwestern Canada. *Precambrian Res.* **336**, 105507 (2020).

32. M. A. Lechte, M. W. Wallace, Sedimentary and tectonic history of the Holowilena Ironstone, a Neoproterozoic iron formation in South Australia. *Sediment. Geol.* **329**, 211–224 (2015).

33. W. V. Preiss, The Adelaide Geosyncline of South Australia and its significance in Neoproterozoic continental reconstruction. *Precambrian Res.* **100**, 21–63 (2000).

34. C. Hu, M. Zhu, Lithofacies and glacio-tectonic deformation structures of the Tiesi'ao/Dongshanfeng Formation on the Yangtze Block, South China: Implications for Sturtian Glaciation dynamics. *Palaeogeogr. Palaeoclimatol. Palaeoecol.* **538**, 109481 (2020).

35. K. K. Turekian, K. H. Wedepohl, Distribution of the elements in some major units of the Earth's crust. *Geol. Soc. Am. Bull.* **72**, 175–192 (1961).

36. E. Ingall, R. Jahnke, Evidence for enhanced phosphorus regeneration from marine sediments overlain by oxygen depleted waters. *Geochim. Cosmochim. Acta* **58**, 2571–2575 (1994).

37. D. E. Canfield, S. W. Poulton, A. H. Knoll, G. M. Narbonne, G. Ross, T. Goldberg, H. Strauss, Ferruginous conditions dominated later Neoproterozoic deep-water chemistry. *Science* **321**, 949–952 (2008).

38. P. Wang, T. J. Algeo, Q. Zhou, W. Yu, Y. Du, Y. Qin, Y. Xu, L. Yuan, W. Pan, Large accumulations of ^{34}S-enriched pyrite in a low-sulfate marine basin: The Sturtian Nanhua Basin, South China. *Precambrian Res.* **335**, 105504 (2019).

39. S. W. Poulton, D. E. Canfield, Ferruginous conditions: A dominant feature of the ocean through Earth's history. *Elements* **7**, 107–112 (2011).

40. J. Ai, N. Zhong, T. Zhang, Y. Zhang, T. Wang, S. C. George, Oceanic water chemistry evolution and its implications for post-glacial black shale formation: Insights from the Cryogenian Datangpo Formation, South China. *Chem. Geol.* **566**, 120083 (2021).

41. D. S. Hardisty, T. W. Lyons, N. Riedinger, T. T. Isson, J. D. Owens, R. C. Aller, D. M. Rye, N. J. Planavsky, C. T. Reinhard, B. C. Gill, A. L. Masterson, D. Asael, D. T. Johnston, An evaluation of sedimentary molybdenum and iron as proxies for pore fluid paleoredox conditions. *Am. J. Sci.* **318**, 527–556 (2018).

42. E. L. Scheller, A. J. Dickson, D. E. Canfield, C. Korte, K. K. Kristiansen, T. W. Dahl, Ocean redox conditions between the snowballs – Geochemical constraints from Arena Formation, East Greenland. *Precambrian Res.* **319**, 173–186 (2018).

43. P. A. E. Pogge Von Strandmann, E. E. Stüeken, T. Elliott, S. W. Poulton, C. M. Dehler, D. E. Canfield, D. C. Catling, Selenium isotope evidence for progressive oxidation of the Neoproterozoic biosphere. *Nat. Commun.* **6**, 10157 (2015).

44. F. Zhang, X. Zhu, B. Yan, B. Kendall, X. Peng, J. Li, T. J. Algeo, S. Romaniello, Oxygenation of a Cryogenian ocean (Nanhua Basin, South China) revealed by pyrite Fe isotope compositions. *Earth Planet. Sci. Lett.* **429**, 11–19 (2015).

45. T. Tyrrell, The relative influences of nitrogen and phosphorus on oceanic primary production. *Nature* **400**, 525–531 (1999).

46. C. T. Reinhard, N. J. Planavsky, B. C. Gill, K. Ozaki, L. J. Robbins, T. W. Lyons, W. W. Fischer, C. Wang, D. B. Cole, K. O. Konhauser, Evolution of the global phosphorus cycle. *Nature* **541**, 386–389 (2017).

47. R. Guilbaud, S. W. Poulton, J. Thompson, K. F. Husband, M. Zhu, Y. Zhou, G. A. Shields, T. M. Lenton, Phosphorus-limited conditions in the early Neoproterozoic ocean maintained low levels of atmospheric oxygen. *Nat. Geosci.* **13**, 296–301 (2020).

48. L. J. Alcott, B. J. W. Mills, A. Bekker, S. W. Poulton, Earth's great oxidation event facilitated by the rise of sedimentary phosphorus recycling. *Nat. Geosci.* **15**, 210–215 (2022).

49. M. A. Kipp, E. E. Stüeken, Biomass recycling and Earth's early phosphorus cycle. *Sci. Adv.* **3**, eaao4795 (2017).

50. T. A. Laakso, E. A. Sperling, D. T. Johnston, A. H. Knoll, Ediacaran reorganization of the marine phosphorus cycle. *Proc. Natl. Acad. Sci. U.S.A.* **117**, 11961–11967 (2020).

51. E. D. Ingall, R. M. Bustin, P. Van Cappellen, Influence of water column anoxia on the burial and preservation of carbon and phosphorus in marine shales. *Geochim. Cosmochim. Acta* **57**, 303–316 (1993).

52. M. D. Krom, R. A. Berner, The diagenesis of phosphorus in a nearshore marine sediment. *Geochim. Cosmochim. Acta* **45**, 207–216 (1981).

53. N. M. Papadomanolaki, W. K. Lenstra, M. Wolthers, C. P. Slomp, Enhanced phosphorus recycling during past oceanic anoxia amplified by low rates of apatite authigenesis. *Sci. Adv.* **8**, eabn2370 (2022).

54. P. Van Cappellen, E. D. Ingall, Benthic phosphorus regeneration, net primary production and ocean anoxia. *Paleoceanography* **9**, 677–692 (1994).

55. K. C. Ruttenberg, R. A. Berner, Authigenic apatite formation and burial in sediments from non-upwelling, continental margin environments. *Geochim. Cosmochim. Acta* **57**, 991–1007 (1993).

56. Y. Xiong, R. Guilbaud, C. L. Peacock, R. P. Cox, D. E. Canfield, M. D. Krom, S. W. Poulton, Phosphorus cycling in Lake Cadagno, Switzerland: A low sulfate euxinic ocean analogue. *Geochim. Cosmochim. Acta* **251**, 116–135 (2019).

57. C. P. Slomp, S. J. Van Der Gaast, W. Van Raaphorst, Phosphorus binding by poorly crystalline iron oxides in North Sea sediments. *Mar. Chem.* **52**, 55–73 (1996).

58. C. P. Slomp, E. H. G. Epping, W. Helder, W. Van Raaphorst, A key role for iron-bound phosphorus in authigenic apatite formation in North Atlantic continental platform sediments. *J. Mar. Res.* **54**, 1179–1205 (1996).

59. S. Roest-Ellis, J. A. Richardson, B. L. Phillips, A. Mehra, S. M. Webb, P. A. Cohen, J. V. Strauss, N. J. Tosca, Tonian carbonates record phosphate-rich shallow seas. *Geochem. Geophys. Geosystems* **24**, e2023GC010974 (2023).

60. M. Tranter, M. J. Sharp, H. R. Lamb, G. H. Brown, B. P. Hubbard, I. C. Willis, Geochemical weathering at the bed of Haut Glacier d'Arolla, Switzerland—A new model. *Hydrol. Process.* **16**, 959–993 (2002).

61. G. M. Cox, T. W. Lyons, R. N. Mitchell, D. Hasterok, M. Gard, Linking the rise of atmospheric oxygen to growth of the continental phosphorus inventory. *Earth Planet. Sci. Lett.* **489**, 28–36 (2018).

62. R. E. Ernst, D. P. G. Bond, S.-H. Zhang, K. L. Buchan, S. E. Grasby, N. Youbi, H. El Bilali, A. Bekker, L. Doucet, Large igneous province record through time and implications for secular environmental changes and geological time-scale boundaries, in *Large Igneous Provinces: A Driver of Global Environmental and Biotic Changes*, R. E. Ernst, A. J. Dickson, A. Bekker, Eds., Geophysical Monograph 255 (AGU, 2021), pp. 3–26.

63. M. T. Hurtgen, M. A. Arthur, G. P. Halverson, Neoproterozoic sulfur isotope, the evolution of microbial sulfur species, and the burial efficiency of sulfide as sedimentary pyrite. *Geology* **33**, 41–44 (2005).

64. D. E. canfield, the early history of atmospheric oxygen: Homage to Robert M. Garrels. *Annu. Rev. Earth Planet. Sci.* **33**, 1–36 (2005).

65. G. Shields, P. Stille, Stratigraphic trends in cerium anomaly in authigenic marine carbonates and phosphates: Diagenetic alteration or seawater signals? *Mineral. Mag.* **62A**, 1387–1388 (1998).

66. K. V. Lau, F. A. Macdonald, K. Maher, J. L. Payne, Uranium isotope evidence for temporary ocean oxygenation in the aftermath of the Sturtian Snowball Earth. *Earth Planet. Sci. Lett.* **458**, 282–292 (2017).

67. G. A. Shields, M. D. Brasier, P. Stille, D. Dorjnamjaa, Factors contributing to high $\delta^{13}C$ values in Cryogenian limestones of western Mongolia. *Earth Planet. Sci. Lett.* **196**, 99–111 (2002).

68. C. Yang, A. D. Rooney, D. J. Condon, X.-H. Li, D. V. Grazhdankin, F. T. Bowyer, C. Hu, F. Macdonald, M. Zhu, The tempo of Ediacaran evolution. *Sci. Adv.* **7**, eabi9643 (2021).

69. A. J. Kaufman, A. H. Knoll, G. M. Narbonne, Isotopes, ice ages, and terminal Proterozoic earth history. *Proc. Natl. Acad. Sci. U.S.A.* **94**, 6600–6605 (1997).

70. D. M. McKirdy, J. M. Burgess, N. M. Lemon, X. Yu, A. M. Cooper, V. A. Gostin, R. J. F. Jenkins, R. A. Both, A chemostratigraphic overview of the late Cryogenian interglacial sequence in the Adelaide Fold-Thrust Belt, South Australia. *Precambrian Res.* **106**, 149–186 (2001).

71. S. W. Poulton, D. E. Canfield, Development of a sequential extraction procedure for iron: Implications for iron partitioning in continentally derived particulates. *Chem. Geol.* **214**, 209–221 (2005).

72. R. Raiswell, D. E. Canfield, Sources of iron for pyrite formation in marine sediments. *Am. J. Sci.* **298**, 219–245 (1998).

73. S. W. Poulton, P. W. Fralick, D. E. Canfield, The transition to a sulphidic ocean ∼1.84 billion years ago. *Nature* **431**, 173–177 (2004).

74. A. Benkovitz, A. Matthews, N. Teutsch, S. W. Poulton, M. Bar-Matthews, A. Almogi-Labin, Tracing water column euxinia in Eastern Mediterranean Sapropels S5 and S7. *Chem. Geol.* **545**, 119627 (2020).

75. K. C. Ruttenberg, Development of a sequential extraction method for different forms of phosphorus in marine sediments. *Limnol. Oceanogr.* **37**, 1460–1482 (1992).

76. J. Thompson, S. W. Poulton, R. Guilbaud, K. A. Doyle, S. Reid, M. D. Krom, Development of a modified SEDEX phosphorus speciation method for ancient rocks and modern iron-rich sediments. *Chem. Geol.* **524**, 383–393 (2019).

77. A. S. Merdith, S. E. Williams, A. S. Collins, M. G. Tetley, J. A. Mulder, M. L. Blades, A. Young, S. Armistead, J. Cannon, S. Zahirovic, R. D. Müller, Extending full-plate tectonic models into deep time: Linking the Neoproterozoic and Phanerozoic. *Earth-Science Rev.* **214**, 103477 (2021).

78. D. I. Benn, G. Le Hir, H. Bao, Y. Donnadieu, C. Dumas, E. J. Fleming, M. J. Hambrey, E. A. McMillan, M. S. Petronis, G. Ramstein, C. T. E. Stevenson, P. M. Wynn, I. J. Fairchild, Orbitally forced ice sheet fluctuations during the Marinoan Snowball Earth glaciation. *Nat. Geosci.* **8**, 704–707 (2015).

79. K. Grey, C. R. Calver, Correlating the ediacaran of Australia. *Geol. Soc. Spec. Publ.* **286**, 115–135 (2007).

80. J. C. Lloyd, M. L. Blades, J. W. Counts, A. S. Collins, K. J. Amos, B. P. Wade, J. W. Halls, S. Hore, A. L. Ball, S. Shahin, M. Drabsch, Neoproterozoic geochronology and provenance of the Adelaide Superbasin. *Precambrian Res.* **350**, 105849 (2020).

81. H. J. Allen, K. Grey, P. W. Haines, C. J. Edgoose, V. J. Normington, *The Cryogenian Aralka Formation, Amadeus Basin: A Basinwide Biostratigraphic Correlation* (Geological Survey of Western Australia, 2018).

82. K. Grey, H.-J. Allen, A. Hill, P. W. Haines, Neoproterozoic biostratigraphy of the Amadeus Basin, in *Proceedings of the Petroleum Exploration Society of Australia (PESA); Central Australian Basins Symposium III, Alice Springs, Northern Territory, 16–17 July*, G. J. Ambrose, J. Scott, Eds. (PESA: Special Publication, 2012), p. 18.

83. M. W. Wallace, A. V. S. Hood, E. M. S. Woon, J. A. Giddings, T. A. Fromhold, The Cryogenian Balcanoona reef complexes of the Northern Flinders Ranges: Implications for Neoproterozoic ocean chemistry. *Palaeogeogr. Palaeoclimatol. Palaeoecol.* **417**, 320–336 (2015).

84. B. O'Connell, M. W. Wallace, A. V. S. Hood, M. A. Lechte, N. J. Planavsky, Iron-rich carbonate tidal deposits, Angepena Formation, South Australia: A redox-stratified Cryogenian basin. *Precambrian Res.* **342**, 105668 (2020).

85. L. A. Riedman, S. M. Porter, G. P. Halverson, M. T. Hurtgen, C. K. Junium, Organic-walled microfossil assemblages from glacial and interglacial Neoproterozoic units of Australia and Svalbard. *Geology* **42**, 1011–1014 (2014).

86. L. Yin, X. Yuan, Radiation of Meso-Neoproterozoic and Early Cambrian protists inferred from the microfossil record of China. *Palaeogeogr. Palaeoclimatol. Palaeoecol.* **254**, 350–361 (2007).

87. J. Wang, Z. X. Li, History of neoproterozoic rift basins in South China: Implications for Rodinia break-up. *Precambrian Res.* **122**, 141–158 (2003).

88. S. H. Zhang, D. A. D. Evans, H. Y. Li, H. C. Wu, G. Q. Jiang, J. Dong, Q. L. Zhao, T. D. Raub, T. S. Yang, Paleomagnetism of the late Cryogenian Nantuo Formation and paleogeographic implications for the South China Block. *J. Asian Earth Sci.* **72**, 164–177 (2013).

89. A. S. Merdith, A. S. Collins, S. E. Williams, S. Pisarevsky, J. D. Foden, D. B. Archibald, M. L. Blades, B. L. Alessio, S. Armistead, D. Plavsa, C. Clark, R. D. Müller, A full-plate global reconstruction of the Neoproterozoic. *Gondw. Res.* **50**, 84–134 (2017).

90. Z. Lan, X. Li, M. Zhu, Z. Q. Chen, Q. Zhang, Q. Li, D. Lu, Y. Liu, G. Tang, A rapid and synchronous initiation of the wide spread Cryogenian glaciations. *Precambrian Res.* **255**, 401–411 (2014).

91. Z. Lan, X. H. Li, Q. Zhang, Q. L. Li, Global synchronous initiation of the 2nd episode of Sturtian glaciation: SIMS zircon U-Pb and O isotope evidence from the Jiangkou Group, South China. *Precambrian Res.* **267**, 28–38 (2015).

92. W. Wei, R. Frei, R. Klaebe, D. Li, G. Y. Wei, H. F. Ling, Redox condition in the Nanhua Basin during the waning of the Sturtian glaciation: A chromium-isotope perspective. *Precambrian Res.* **319**, 198–210 (2018).

93. L. Feng, J. Huang, D. Lu, Q. Zhang, Major and trace element geochemistry of the Neoproterozoic syn-glacial Fulu iron formation, South China. *Geol. Mag.* **154**, 1371–1380 (2017).

94. C. Zhou, M. H. Huyskens, X. Lang, S. Xiao, Q. Z. Yin, Calibrating the terminations of Cryogenian global glaciations. *Geology* **47**, 251–254 (2019).

95. A. D. Rooney, J. V. Strauss, A. D. Brandon, F. A. Macdonald, A Cryogenian chronology: Two long-lasting synchronous Neoproterozoic glaciations. *Geology* **43**, 459–462 (2015).

96. X. Bao, S. Zhang, G. Jiang, H. Wu, H. Li, X. Wang, Z. An, T. Yang, Cyclostratigraphic constraints on the duration of the Datangpo Formation and the onset age of the Nantuo (Marinoan) glaciation in South China. *Earth Planet. Sci. Lett.* **483**, 52–63 (2018).

97. L. Yin, Microfossils from late Proterozoic manganese ore deposits in western Hunan Province and eastern Guizhou Province, South China. *Sci. China Ser. B.* **8**, 861–866 (1990).

98. D. E. Canfield, R. Raiswell, J. T. Westrich, C. M. Reaves, R. A. Berner, The use of chromium reduction in the analysis of reduced inorganic sulfur in sediments and shales. *Chem. Geol.* **54**, 149–155 (1986).

99. L. J. Alcott, A. J. Krause, E. U. Hammarlund, C. J. Bjerrum, F. Scholz, Y. Xiong, A. J. Hobson, L. Neve, B. W. Mills, C. Marz, B. Schnetger, A. Bekker, S. W. Poulton, Development of iron speciation reference materials for palaeoredox analysis. *Geostand. Geoanalytical Res.* **44**, 581–591 (2020).

100. V. Pasquier, D. A. Fike, S. Révillon, I. Halevy, A global reassessment of the controls on iron speciation in modern sediments and sedimentary rocks: A dominant role for diagenesis. *Geochim. Cosmochim. Acta* **335**, 211–230 (2022).

101. M. O. Clarkson, S. W. Poulton, R. Guilbaud, R. A. Wood, Assessing the utility of Fe/Al and Fe-speciation to record water column redox conditions in carbonate-rich sediments. *Chem. Geol.* **382**, 111–122 (2014).

102. D. E. Canfield, T. W. Lyons, R. Raiswell, A model for iron deposition to euxinic black sea sediments. *Am. J. Sci.* **296**, 818–834 (1996).

103. G.-Y. Wei, T. Chen, S. W. Poulton, Y.-B. Lin, T. He, X. Shi, J. Chen, H. Li, S. Qiao, J. Liu, D. Li, H.-F. Ling, A chemical weathering control on the delivery of particulate iron to the continental shelf. *Geochim. Cosmochim. Acta* **308**, 204–216 (2021).

104. S. W. Poulton, R. Raiswell, The low-temperature geochemical cycle of iron: From continental fluxes to marine sediment deposition. *Am. J. Sci.* **302**, 774–805 (2002).

105. J. L. Kirschvink, Late Proterozoic low-latitude global glaciation: The snowball Earth, in *The Proterozoic Biosphere: A Multidisciplinary Study*, J. W. Schopf, C. Klein, D. Des Maris, Eds. (Cambridge Univ. Press, 1992), pp. 51–52.

106. P. F. Hoffman, A. J. Kaufman, G. P. Halverson, D. P. Schrag, A Neoproterozoic snowball Earth. *Science* **281**, 1342–1346 (1998).

107. J. Ai, N. Zhong, S. C. George, Y. Zhang, L. Yao, T. Wang, Evolution of paleo-weathering during the late Neoproterozoic in South China: Implications for paleoclimatic conditions and organic carbon burial. *Palaeogeogr. Palaeoclimatol. Palaeoecol.* **555**, 109843 (2020).

108. P. Wang, Y. Du, W. Yu, T. J. Algeo, Q. Zhou, Y. Xu, L. Qi, L. Yun, W. Pan, The chemical index of alteration (CIA) as a proxy for climate change during glacial-interglacial transitions in Earth history. *Earth-Science Rev.* **201**, 103032 (2020).

109. G. Zhu, T. Li, Z. Zhang, K. Zhao, H. Song, P. Wang, H. Yan, H. Song, Nitrogen isotope evidence for oxgenated upper ocean during the Cryogenian interglacial period. *Chem. Geol.* **604**, 120929 (2022).

110. R. Raiswell, R. Newton, S. H. Bottrell, P. M. Coburn, D. E. G. Briggs, D. P. G. Bond, S. W. Poulton, Turbidite depositional influences on the diagenesis of Beecher's Trilobite Bed and the Hunsrück Slate; sites of soft tissue pyritization. *Am. J. Sci.* **308**, 105–129 (2008).

111. R. Raiswell, R. Newton, P. B. Wignall, An indicator of water-column anoxia: Resolution of biofacies variations in the Kimmeridge clay (Upper Jurassic, UK). *J. Sediment. Res.* **71**, 286–294 (2001).

112. S. W. Poulton, *The Iron Speciation Paleoredox Proxy* (Cambridge Univ. Press, 2021)

113. R. Raiswell, D. S. Hardisty, T. W. Lyons, D. E. Canfield, J. D. Owens, N. J. Planavsky, S. W. Poulton, C. T. Reinhard, The iron paleoredox proxies: A guide to the pitfalls, problems and proper practice. *Am. J. Sci.* **318**, 491–526 (2018).

114. D. B. Cole, S. Zhang, N. J. Planavsky, A new estimate of detrital redox-sensitive metal concentrations and variability in fluxes to marine sediments. *Geochim. Cosmochim. Acta* **215**, 337–353 (2017).

115. R. L. Rudnick, S. Gao, Composition of the continental crust, in *The crust*, R. L. Rudnick, H. D. Holland, K. K. Turekian, Eds. (Elsevier, 2003), pp. 1–64.

116. N. Tribovillard, T. J. Algeo, T. Lyons, A. Riboulleau, Trace metals as paleoredox and paleoproductivity proxies: An update. *Chem. Geol.* **232**, 12–32 (2006).

117. T. J. Algeo, N. Tribovillard, Environmental analysis of paleoceanographic systems based on molybdenum-uranium covariation. *Chem. Geol.* **268**, 211–225 (2009).

118. M. Kunzmann, G. P. Halverson, C. Scott, W. G. Minarik, B. A. Wing, Geochemistry of Neoproterozoic black shales from Svalbard: Implications for oceanic redox conditions spanning Cryogenian glaciations. *Chem. Geol.* **417**, 383–393 (2015).

119. A. I. Sheen, B. Kendall, C. T. Reinhard, R. A. Creaser, T. W. Lyons, A. Bekker, S. W. Poulton, A. D. Anbar, A model for the oceanic mass balance of rhenium and implications for the extent of Proterozoic ocean anoxia. *Geochim. Cosmochim. Acta* **227**, 75–95 (2018).

120. J. L. Morford, S. Emerson, The geochemistry of redox sensitive trace metals in sediments. *Geochim. Cosmochim. Acta* **63**, 1735–1750 (1999).

121. J. Crusius, S. Calvert, T. Pedersen, D. Sage, Rhenium and molybdenum enrichments in sediments as indicators of oxic, suboxic and sulfidic conditions of deposition. *Earth Planet. Sci. Lett.* **145**, 65–78 (1996).

122. J. L. Morford, S. R. Emerson, E. J. Breckel, S. H. Kim, Diagenesis of oxyanions (V, U, Re, and Mo) in pore waters and sediments from a continental margin. *Geochim. Cosmochim. Acta* **69**, 5021–5032 (2005).

123. C. A. Partin, A. Bekker, N. J. Planavsky, C. T. Scott, B. C. Gill, C. Li, V. Podkovyrov, A. Maslov, K. O. Konhauser, S. V. Lalonde, G. D. Love, S. W. Poulton, T. W. Lyons, Large-scale fluctuations in Precambrian atmospheric and oceanic oxygen levels from the record of U in shales. *Earth Planet. Sci. Lett.* **369–370**, 284–293 (2013).

124. C. T. Reinhard, N. J. Planavsky, L. J. Robbins, C. A. Partin, B. C. Gill, S. V. Lalonde, A. Bekker, K. O. Konhauser, T. W. Lyons, Proterozoic ocean redox and biogeochemical stasis. *Proc. Natl. Acad. Sci. U.S.A.* **110**, 5357–5362 (2013).

125. N. Tribovillard, T. J. Algeo, F. Baudin, A. Riboulleau, Analysis of marine environmental conditions based onmolybdenum-uranium covariation—Applications to Mesozoic paleoceanography. *Chem. Geol.* **324–325**, 46–58 (2012).

126. C. E. Barnes, J. K. Cochran, Uranium removal in oceanic sediments and the oceanic U balance. *Earth Planet. Sci. Lett.* **97**, 94–101 (1990).

127. G. P. Klinkhammer, M. R. Palmer, Uranium in the oceans: Where it goes and why. *Geochim. Cosmochim. Acta* **55**, 1799–1806 (1991).

128. L. Olson, K. A. Quinn, M. G. Siebecker, G. W. Luther, D. Hastings, J. L. Morford, Trace metal diagenesis in sulfidic sediments: Insights from Chesapeake Bay. *Chem. Geol.* **452**, 47–59 (2017).

129. G. R. Helz, C. V. Miller, J. M. Charnock, J. F. W. Mosselmans, R. A. D. Pattrick, C. D. Garner, D. J. Vaughan, Mechanism of molybdenum removal from the sea and its concentration in black shales : EXAFS evidence. *Geochim. Cosmochim. Acta* **60**, 3631–3642 (1996).

130. T. J. Algeo, T. W. Lyons, Mo–total organic carbon covariation in modern anoxic marine environments: Implications for analysis of paleoredox and paleohydrographic conditions. *Paleoceanography* **21**, PA1016 (2006).

131. C. Scott, T. W. Lyons, A. Bekker, Y. Shen, S. W. Poulton, X. Chu, A. D. Anbar, Tracing the stepwise oxygenation of the Proterozoic ocean. *Nature* **452**, 456–459 (2008).

132. J. R. Creveling, D. T. Johnston, S. W. Poulton, B. Kotrc, C. März, D. P. Schrag, A. H. Knoll, Phosphorus sources for phosphatic Cambrian carbonates. *Bull. Geol. Soc. Am.* **126**, 145–163 (2014).

133. F. Horton, Did phosphorus derived from the weathering of large igneous provinces fertilize the Neoproterozoic ocean? *Geochem. Geophys. Geosystems* **16**, 1723–1738 (2015).

134. S. Sharoni, I. Halevy, Geologic controls on phytoplankton elemental composition. *Proc. Natl. Acad. Sci. U.S.A.* **119**, e2113263118 (2022).

135. N. J. Planavsky, The elements of marine life. *Nat. Geosci.* **7**, 855–856 (2014).

136. W. Yu, T. J. Algeo, Y. Du, Q. Zhou, P. Wang, Y. Xu, L. Yuan, W. Pan, Newly discovered Sturtian cap carbonate in the Nanhua Basin, South China. *Precambrian Res.* **293**, 112–130 (2017).

137. C. Zhou, R. Tucker, S. Xiao, Z. Peng, X. Yuan, Z. Chen, New constraints on the ages of Neoproterozoic glaciations in south China. *Geology* **32**, 437–440 (2004).

138. D. M. McKirdy, J. M. Burgess, N. M. Lemon, X. Yu, A. M. Cooper, V. A. Gostin, R. J. F. Jenkins, R. A. Both, A chemostratigraphic overview of the late Cryogenian interglacial sequence in the Adelaide Fold-Thrust Belt, South Australia. *Precambrian Res.* **106**, 149–186 (2001).

139. M. Ader, M. Macouin, R. I. F. Trindade, M.-H. Hadrien, Z. Yang, Z. Sun, J. Besse, A multilayered water column in the Ediacaran Yangtze platform? Insights from carbonate and organic matter paired $\delta^{13}C$. *Earth Planet. Sci. Lett.* **288**, 213–227 (2009).

140. M. Ader, P. Sansjofre, G. P. Halverson, V. Busigny, R. I. F. Trindade, M. Kunzmann, A. C. R. Nogueira, Ocean redox structure across the Late Neoproterozoic Oxygenation Event: A nitrogen isotope perspective. *Earth Planet. Sci. Lett.* **396**, 1–13 (2014).

141. R. P. Anderson, I. J. Fairchild, N. J. Tosca, A. H. Knoll, Microstructures in metasedimentary rocks from the Neoproterozoic Bonahaven Formation, Scotland: Microconcretions, impact spherules, or microfossils? *Precambrian Res.* **233**, 59–72 (2013).

142. M. Bau, P. Dulski, Distribution of yttrium and rare-earth elements in the Penge and Kuruman iron-formations, Transvaal Supergroup, South Africa. *Precambrian Res.* **79**, 37–55 (1986).

143. J. Bertrand-Sarfati, A. Siedlecka, Columnar stromatolites of the terminal precambrian porsanger dolomite and grasdal formation of Finnmark, North Norway. *Norsk Geologisk Tidsskrift* **60**, 1–27 (1980).

144. T. Bosak, D. J. G. Lahr, S. B. Pruss, F. A. Macdonald, L. Dalton, E. Matys, Agglutinated tests in post-Sturtian cap carbonates of Namibia and Mongolia. *Earth Planet. Sci. Lett.* **308**, 29–40 (2011).

145. T. Bosak, F. Macdonald, D. Lahr, E. Matys, Putative cryogenian ciliates from Mongolia. *Geology* **39**, 1123–1126 (2011).

146. T. Bosak, D. J. G. Lahr, S. B. Pruss, F. A. Macdonald, A. J. Gooday, L. Dalton, E. D. Matys, Possible early foraminiferans in post-Sturtian (716-635 Ma) cap carbonates. *Geology* **40**, 67–70 (2012).

147. C. K. B. Brain, A. R. Prave, K. H. Hoffmann, A. E. Fallick, A. Botha, D. A. Herd, C. Sturrock, I. Young, D. J. Condon, S. G. Allison, The first animals: Ca. 760-million-year-old sponge-like fossils from Namibia. *S. Afr. J. Sci.* **108**, 1–8 (2012).

148. M. D. Brasier, G. Shields, V. N. Kuleshov, E. A. Zhegallo, Integrated chemo- and biostratigraphic calibration of early animal evolution: Neoproterozoic-early Cambrian of southwest Mongolia. *Geol. Mag.* **133**, 445–485 (1996).

149. M. D. Brasier, G. Shields, Neoproterozoic chemostratigraphy and correlation of the Port Askaig glaciation, Dalradian Supergroup of Scotland. *J. Geol. Soc. London* **157**, 909–914 (2000).

150. C. Calver, Isotope stratigraphy of the Neoproterozoic Togari group, Tasmania. *Aust. J. Earth Sci.* **45**, 865–874 (1998).

151. X. Chen, S. Romaniello, A. D. Hermann, D. S. Hardisty, B. C. Gill, A. D. Anbar, Diagenetic effects on uranium isotope fractionation in carbonate sediments from the Bahamas. *Geochim. Cosmochim. Acta* **237**, 294–311 (2018).

152. M. Cheng, C. Li, X. Chen, L. Zhou, T. J. Algeo, H.-F. Ling, L.-J. Feng, C.-S. Jin, Delayed neoproterozoic ocean oxygenation: Evidence from mo isotopes of the cryogenian datangpo formation. *Precambrian Res.* **319**, 187–197 (2018).

153. P. A. Cohen, F. A. Macdonald, S. Pruss, E. Matys, T. Bosak, Fossils of putative marine algae from the Cryogenian glacial interlude of Mongolia. *Palaios* **30**, 238–247 (2015).

154. L. A. Dalton, T. Bosak, F. A. Macdonald, D. J. G. Lahr, S. B. Pruss, Preservational and morphological variability of assemblages of agglutinated eukaryotes in Cryogenian cap carbonates of northern Namibia. *Palaios* **28**, 67–79 (2013).

155. H. J. W. de Baar, M. P. Bacon, P. G. Brewer, K. W. Bruland, Rare earth elements in the Pacific and Atlantic Oceans. *Geochim. Cosmochim. Acta* **49**, 1943–1959 (1985).

156. I. J. Fairchild, P. Bonnard, T. Davies, E. J. Fleming, N. Grassineau, G. P. Halverson, M. J. Hambrey, E. M. McMillan, E. McKay, I. J. Parkinson, C. T. E. Stevenson, The late cryogenian warm interval, NE Svalbard: Chemostratigraphy and genesis. *Precambrian Res.* **281**, 128–154 (2016).

157. L.-J. Feng, X.-L. Chu, J. Huang, Q.-R. Zhang, H.-J. Chang, Reconstruction of paleo-redox conditions and early sulfur cycling during deposition of the Cryogenian Datangpo Formation in South China. *Gondw. Res.* **18**, 632–637 (2010).

158. G. P. Halverson, P. F. Hoffman, D. P. Schrag, A major perturbation of the carbon cycle before the Ghaub glaciation (Neoproterozoic) in Namibia: Prelude to snowball Earth? *Geochem. Geophys. Geosystems* **3**, 1–24 (2002).

159. P. F. Hoffman, D. P. Schrag, The Snowball Earth hypothesis: Testing the limits of global change. *Terra Nova* **14**, 129–155 (2002).

160. P. F. Hoffman, G. P. Halverson, E. W. Domack, A. C. Maloof, N. L. Swanson-Hysell, G. M. Cox, Cryogenian glaciations on the southern tropical paleomargin of Laurentia (NE Svalbard and East Greenland), and a primary origin for the upper Russoya (Islay) carbon isotope excursion. *Precambrian Res.* **206-207**, 137–158 (2012).

161. H. J. Hofmann, G. M. Narbonne, J. D. Aitken, Ediacaran remains from intertillite beds in northwestern Canada. *Geology* **18**, 1199–1202 (1990).

162. A. v. S. Hood, M. W. Wallace, Extreme ocean anoxia during the Late Cryogenian recorded in reefal carbonates of Southern Australia. *Precambrian Res.* **261**, 96–111 (2015).

163. M. T. Hurtgen, M. A. Arthur, N. S. Suits, A. J. Kaufman, The sulfur isotopic composition of Neoproterozoic seawater sulfate: Implications for a snowball Earth? *Earth Planet. Sci. Lett.* **203**, 413–429 (2002).

164. D. T. Johnston, F. A. Macdonald, B. C. Gill, P. F. Hoffman, D. P. Schrag, Uncovering the Neoproterozoic carbon cycle. *Nature* **483**, 320–324 (2012).

165. M. Kunzmann, T. H. Bui, P. W. Crockford, G. P. Halverson, C. Scott, T. W. Lyons, B. A. Wing, Bacterial sulfur disproportionation constrains timing of Neoproterozoic oxygenation. *Geology* **45**, 207–210 (2017).

166. M. G. Lawrence, A. Greig, K. D. Collerson, B. S. Kamber, Rare earth element and yttrium variability in South East Queensland waterways. *Aquat. Geochem.* **12**, 39–72 (2006).

167. C. Li, G. D. Love, T. W. Lyons, C. T. Scott, L. Feng, J. Huang, H. Chang, Q. Zhang, X. Chu, Evidence for a redox stratified Cryogenian marine basin, Datangpo Formation, South China. *Earth Planet. Sci. Lett.* **331-332**, 246–256 (2012).

168. H. F. Ling, X. Chen, D. Li, D. Wang, G. A. Shields-Zhou, M. Zhu, Cerium anomaly variations in Ediacaran-earliest Cambrian carbonates from the Yangtze Gorges area, South China: Implications for oxygenation of coeval shallow seawater. *Precambrian Res.* **225**, 110–127 (2013).

169. F. A. Macdonald, W. C. McClelland, D. P. Schrag, W. P. Macdonald, Neoproterozoic glaciation on a carbonate platform margin in Arctic Alaska and the origin of the North Slope subterrane. *GSA Bull.* **121**, 448–473 (2009).

170. F. A. Macdonald, J. V. Strauss, E. A. Sperling, G. P. Halverson, G. M. Narbonne, D. T. Johnston, M. Kunzmann, D. P. Schrag, J. A. Higgins, The stratigraphic relationship between the Shuram carbon isotope excursion, the oxygenation of Neoproterozoic oceans, and the first appearance of the Ediacara biota and bilaterian trace fossils in northwestern Canada. *Chem. Geol.* **362**, 250–272 (2013).

171. A. C. Maloof, C. V. Rose, R. Beach, B. M. Samuels, C. C. Calmet, D. H. Erwin, G. R. Poirier, N. Yao, F. J. Simons, Possible animal-body fossils in pre-Marinoan limestones from South Australia. *Nat. Geosci.* **3**, 653–659 (2010).

172. S. B. McLennan, Rare earth elements in sedimentary rocks. Influence of provenance and sedimentary processes, in *Geochemistry and Mineralogy of the Rare Earth Elements*, B. R. Lipin, G. A. McKay, Eds., (Mineralogical Society of America, 1989), Special Paper, pp. 169–200.

173. K. R. Moore, T. Bosak, F. A. Macdonald, D. J. G. Lahr, S. Newman, C. Settens, S. B. Pruss, Biologically agglutinated eukaryotic microfossil from Cryogenian cap carbonates. *Geobiology* **15**, 499–515 (2017).

174. A. R. Prave, A. E. Fallick, C. W. Thomas, C. M. Graham, A composite C-isotope profile for the Neoproterozoic Dalradian Supergroup of Scotland and Ireland. *J. Geol. Soc. London* **166**, 845–857 (2009).

175. M. E. Raaben, V. V. Lyubtsov, A. A. Predovsky, Correlation of stromatolitic formations of northern Norway (Finnmark) and northwestern Russian (Kildin Island and Kanin Peninsula). *Norges Geologiske Undersøkelse Special Publication* **7**, 233–246 (1995).

176. A. S. Rodler, R. Frei, C. Gaucher, C. Korte, S. A. Rosing, G. J. B. Germs, Multiproxy isotope constraints on ocean compositional changes across the late Neoproterozoic Ghaub glaciation, Otavi Group, Namibia. *Precambrian Res.* **298**, 306–324 (2017).

177. E. A. Sperling, C. J. Wolock, A. S. Morgan, B. C. Gill, M. Kunzmann, G. P. Halverson, F. A. Macdonald, A. H. Knoll, D. T. Johnston, Statistical analysis of iron geochemical data suggests limited late Proterozoic oxygenation. *Nature* **523**, 451–454 (2015).

178. E. A. Sperling, C. Carbone, J. V. Strauss, D. T. Johnston, G. M. Narbonne, F. A. Macdonald, Oxygen, facies, and secular controls on the appearance of Cryogenian and Ediacaran body and trace fossils in the Mackenzie Mountains of northwestern Canada. *GSA Bull.* **128**, 558–575 (2016).

179. N. L. Swanson-Hysell, C. V. Rose, C. C. Calmet, G. P. Halverson, M. T. Hurtgen, A. C. Maloof, Cryogenian glaciation and the onset of carbon-isotope decoupling. *Science* **328**, 608–611 (2010).

180. J. E. Tesdal, E. D. Galbraith, M. Kienast, Nitrogen isotopes in bulk marine sediment: Linking seafloor observations with subseafloor records. *Biogeosciences* **10**, 101–118 (2013).

181. G. Vidal, Late Precambrian acritarchs from the Eleonore Bay Group and Tillite Group in East Greenland: A preliminary report. *Grønlands Geologiske Undersøgelse Rapport* **78**, 1–19 (1976).

182. G. Vidal, Acritarchs from the Upper Proterozoic and Lower Cambrian of East Greenland. *Grønlands Geologiske Undersøgelse Bulletin* **134**, 1–40 (1979).

183. G. Vidal, Micropalaeontology and biostratigraphy of the Upper Proterozoic and Lower Cambrian sequence in East Finnmark, Northern Norway. *Norges Geologiske Undersøkelse* **362**, 1–53 (1981).

184. M. W. Wallace, A. v. S. Hood, A. Shuster, A. Greig, N. J. Planavsky, C. P. Reed, Oxygenation history of the Neoproterozoic to early Phanerozoic and the rise of land plants. *Earth Planet. Sci. Lett.* **466**, 12–19 (2017).

185. W. Wei, D. Wang, D. Li, H. Ling, X. Chen, G. Wei, F. Zhang, X. Zhu, B. Yan, The marine redox change and nitrogen cycle in the early cryogenian interglacial time: Evidence from nitrogen isotopes and mo contents of the basal datangpo formation, Northeastern Guizhou, South China. *J. Earth Sci.* **7**, 233–241 (2016).

186. G.-Y. Wei, W. Wei, D. Wang, T. Li, X. Yang, G. A. Shields, F. Zhang, G. Li, T. Chen, T. Yang, H.-F. Ling, Enhanced chemical weathering triggered an expansion of euxinic seawater in the aftermath of the Sturtian glaciation. *Earth Planet. Sci. Lett.* **539**, 116244 (2020).

187. C. Wu, T. Yang, G. A. Shields, X. Bian, B. Gao, H. Ye, W. Li, Termination of Cryogenian ironstone deposition by deep ocean euxinia. *Geochem. Perspect. Lett.* **15**, 1–5 (2020).

188. Q. Ye, J. Tong, S. Xiao, S. Zhu, Z. An, L. Tian, J. Hu, The survival of benthic macroscopic phototrophs on a Neoproterozoic snowball Earth. *Geology* **43**, 507–510 (2015).

189. Y. Ye, H. Wang, L. Zhai, X. Wang, C. Wu, S. Zhang, Contrasting Mo-U enrichments of the basal Datangpo Formation in South China: Implications for the Cryogenian interglacial ocean redox. *Precambrian Res.* **315**, 66–74 (2018).

190. H. Yoshioka, Y. Asahara, B. Tojo, S.-I. Kawakami, Systematic variations in C, O, and Sr isotopes and elemental concentrations in Neoproterozoic carbonates in Namibia: Implications for a glacial to interglacial transition. *Precambrian Res.* **124**, 69–85 (2003).

191. W. Yu, T. J. Algeo, Y. Du, B. Maynard, H. Guo, Q. Zhou, T. Peng, P. Wang, L. Yuan, Genesis of Cryogenian Datangpo manganese deposit: Hydrothermal influence and episodic post-glacial ventilation of Nanhua Basin, South China. *Palaeogeograph. Palaeoclimatolog. Palaeoecolog.* **459**, 321–337 (2016).
